# Supplementary material for: Direct-to-biology, automated, nano-scale synthesis, and phenotypic screening-enabled E3 ligase modulator discovery
Source: Nat Commun. 2023 Dec 19;14:8437. doi: 10.1038/s41467-023-43614-3 (PMC10730884; doi:10.1038/s41467-023-43614-3)
Supplement: Supplementary file 1 — Supplementary Information [file 41467_2023_43614_MOESM1_ESM.pdf]

Direct-to-Biology, Automated, Nano-Scale Synthesis, and Phenotypic Screening-Enabled E3 Ligase Modulator Discovery

Zefeng Wang†[1], Shabnam Shaabani†[1], Xiang Gao†[2], Yuen Lam Dora Ng[3], Valeriia Sapozhnikova[3,4], Philipp Mertins[5], Jan Krönke[3,4]* and Alexander Dömling[1,6]*

[1] University of Groningen, Department of Drug Design, A. Deusinglaan 1, 9713 AV Groningen, The Netherlands

[2] Department of Internal Medicine III, University Hospital Ulm, 89081 Ulm, Germany

[3] Department of Hematology, Oncology and Cancer Immunology, Charité - Universitätsmedizin Berlin, corporate member of Freie Universität Berlin and Humboldt-Universität zu Berlin, Berlin, Germany

[4] German Cancer Consortium (DKTK) partner site Berlin and German Cancer Research Center (DKFZ), Heidelberg, Germany

[5] Max Delbrück Center for Molecular Medicine, Berlin, Germany + Berlin Institute of Health, Berlin, German

[6] Institute of Molecular and Translational Medicine, Faculty of Medicine and Dentistry and Czech Advanced Technology and Research Institute, Palackȳ University in Olomouc, Olomouc, Czech Republic

†The authors contributed equally

*Correspondence: jan.kroenke@charite.de; alexander.domling@upol.cz;

Table of contents

1. General information 3
1.1 Materials and methods 3
2. Optimization of the reaction conditions for isocyanide synthesis 4
3. Nano-scale automated chemistry 5
3.1 General materials 5
3.2 Instrumentation 5
3.3 Stock solution preparation 5
3.4 Nano-scale synthesis 5
3.5 Quality Control (QC) 21
3.6 Automated analysis of mass spectrometry data 21
4. Heat plots 39
5. Statistical reaction analysis 40
6. Phenotypic screening of the destination plate 44
7. CRBN KO RPMI/8226 cell viability assay 45
8. General experimental data for the mmol scale synthesis 45
8.1 General procedure for isocyanide synthesis 45
8.2 General mmol synthesis procedure for Multiple Component Reaction 47
^{1}H, ^{13}C NMR spectra of mmol scale reaction 56
References 77

1. General information

1.1 Materials and methods

Chemistry:

Nuclear magnetic resonance spectra (NMR) were recorded on a Bruker Avance 500 spectrometer (^{1}H NMR (500 MHz), ^{13}C NMR (126 MHz)). Chemical shifts for ^{1}H NMR were reported relative to TMS (δ 0 ppm) or internal solvent peak ($CDCl_3$ δ 7.26 ppm, DMSO-d_6 δ 2.50 ppm, CD_3OD δ 3.31 ppm or Acetone-d_6 δ 2.05 ppm) and coupling constants were in hertz (Hz). The following abbreviations were used for spin multiplicity: s = singlet, d = doublet, t = triplet, dd = double doublet, m = multiplet, brs = broad singlet. Chemical shifts for ^{13}C NMR reported in ppm relative to the solvent peak ($CDCl_3$ δ 77.16 ppm, DMSO-d_6 δ 39.50 ppm, CD_3OD δ 49.00 ppm, Acetone-d_6 δ 29.84 ppm and 206.26 ppm). Thin layer chromatography was performed on precoated silica gel 60 F_{254} plates (Merck, Darmstadt). Reagents were available from commercial suppliers and used without any purification unless otherwise noted. Electrospray ionization mass spectra (ESI-MS) were recorded on a Waters Investigator Semi-prep 15 SFC-MS instrument. High-resolution mass spectra were recorded using a QTOF Bruker Maxis Plus, mass range 100-1500 m/z, spectra rate 2.00 Hz. All the starting materials used in the chemistry synthesis were purchased from (A2B chemical, Fluorochem Ltd, Sigma-Aldrich company, TCI chemicals).

Cell culture:

MM.1S, NCI-H929, RPMI/8226, HEL, K562, Nalm-6 cells were obtained from the American Type Culture Collection (ATCC) or the Deutsche Sammlung von Mikroorganismen and Zellkulturen (DSMZ). Cells were cultured in RPMI1640 medium (Gibco) or DMEM (Gibco) supplemented with 10% fetal bovine serum (FBS) (Merck Millipore), 1% Penicillin/streptomycin (Gibco), and 1% L-Glutamine (Pan Biotech). NCI-H929 cells were cultured in the presence of 0,05mM 2-Mercapto-ethanol (Milipore). Cells were grown at 37 ℃ with 5% CO_2 in humidified atmosphere. All cells were cultured in proper density and split every 2-3 days.

Antibodies:

Primary antibodies used for Western blotting from Cell Signaling (Danvers, USA) include IKZF3 (clone D1C1E, #15103, RRID:AB_2744524, 1:1000), IKZF1 (clone D6N9Y, #14859, RRID:AB_2744523, 1:1000), IRF4 (clone D43H10, #4299, RRID:AB_10547141, 1:1000), c-Myc (clone D84C12, #5605, RRID:AB_1903938, 1:1000), eRF3(#14980,RRID:AB_2798677,1:1000), Cas9(clone 7A9-3A3, #14697, AB_2750916,1:1000); antibodies from Sigma-Aldrich (St. Louis, USA) include anti-alpha-Tubulin (#T5168, RRID: AB_477579, 1:7000), CRBN(# HPA045910,RRID:AB_10960409,1:1000. Secondary antibodies used for Western blotting from Cell Signaling include anti-rabbit IgG HRP-linked antibody (#7074, 1:5000), anti-mouse IgG HRP-linked antibody (#7076, 1:5000).

2. Optimization of the reaction conditions for isocyanide synthesis

In order to optimize the isocyanide synthesis reaction condition, various solvents (3 mL) and reaction temperatures were screened (**Table S1**). The desired isocyanide was formed in highest yields in anhydrous THF as solvent and by dropwise $POCl_3$ (2.0 mmol) addition at -78 °C (**Table S1**, entries **3** and **4**). After $POCl_3$ dropwise addition at -78 °C (15 min), immediately increasing reaction temperature increase to -20 °C gave the desired isocyanide in 40% yield after 5 hours (**Table S1**, entry **3**). However, slowly increasing reaction temperature to -20 °C over 5 hours resulted in higher isolated yields of 65% (**Table S1**, entry **4**).

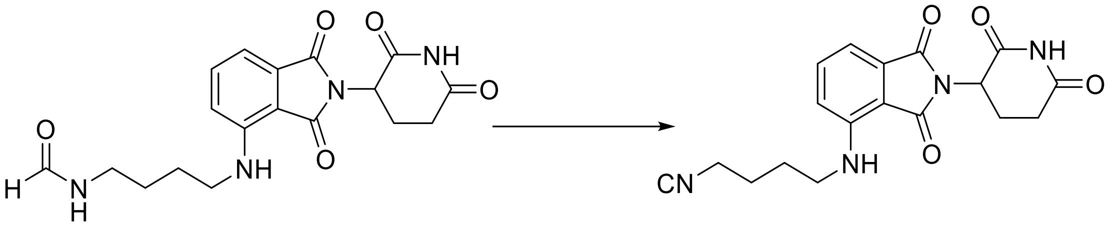

Table 1: Optimization of the 2-(2,6-dioxopiperidin-3-yl)-4-((4-isocyanobutyl)amino)isoindoline-1,3-dione synthesis[a]

Entry	Solvent	Temperature	Time	Yield [b]
**1**	DCM	0 °C[c] to r.t.[d]	overnight	10 %
**2**	THF (anhydrous)	0 °C[c]to r.t.[d]	overnight	15 %
**3**	THF (anhydrous)	-78 °C[c] to - 20 °C[d]	5 hours	40 %
**4**	THF (anhydrous)	-78 °C[c] to -20 °C[d] over 5 hours	5 hours	65 %

[a] Reaction conditions: Formamide (1 mmol), $POCl_3$ (2 mmol), Et_3N (5 mmol), solvent (3 mL).

[b] Isolated yield

[c] Reaction temperature while $POCl_3$ dropwise addition

[d] Reaction temperature increase after $POCl_3$ addition

3. Nano-scale automated chemistry

3.1 General materials

Stock solutions were prepared in glass flat bottom vials (Screening devices, Catalog#: 9920-812FBT, 2.0 mL (Topas) Plate), and they were stored at -20 °C.
Nanomole-scale chemistry was performed using 96-well Dispendix I.DOT PURE polypropylene plate, as source plate (60 μm orifice wells). Greiner bio-one, 384-well PCR polypropylene plate (LOT#: E19043P9, REF#: 785290) were used as destination plate.
384-well destination plates were sealed by a sealing tape (Thermo Scientific, Catalog#: 232701, polyolefin acrylate) and were stored at -20 °C.

3.2 Instrumentation

The I.DOT One liquid handler (Dispendix) was used in order to transfer nL droplets of starting materials from the 96-well source plate to the 384-well destination plate, according to the producers handling manual.

3.3 Stock solution preparation

The stock solutions of the aldehyde/ketone (including formaldehyde 37% w/w aq solution), amine and carboxylic building blocks were prepared as 0.5 M TFE, the isocyanide as 0.25 M TFE and the $TMSN_3$ as 0.6 M TFE.

3.4 Nano-scale synthesis

The stock solutions were dispensed to a 96-well source plate using Eppendorf multi-channel pipettes.
The I.DOT was used as a dispensing instrument to transfer the four components of starting materials into the corresponding well in the destination plate. I.DOT Assay Studio software with the pick list as a csv file was used.
In order to generate a random library of products (N=384), a modified version of our previously reported program RandReactor was used.[1] The smiles files of the starting materials with the corresponding location in the source plate and mrv file of reaction were the input of the RandReactor program. The smiles file of the randomly generated products with their corresponding locations in the source and destination plate were the output of the RandReactor program. The smiles file was converted to a csv file which was the required format for I.DOT Assay Studio software.
Once the starting materials transfer was completed (~20 min), the destination plate was covered with the sealing tape and was then placed for 24 h on an orbital shaker at room

temperature. The sealed plates were stored at -20 °C for further processing. The structures of the products are shown in **Table S2**.

Table 2. Heat plots with product structures, green ☐ for major product formation, yellow ☐ for medium product formation and blue ☐ for no product formation.

	A	B	C	D
**1**	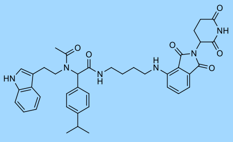	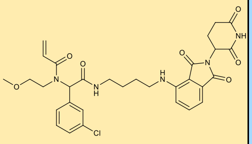	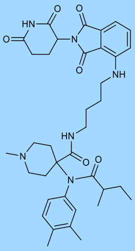	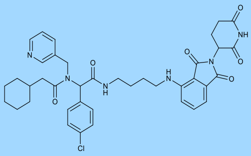
**2**	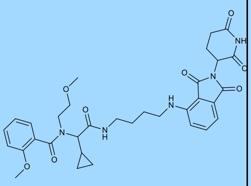	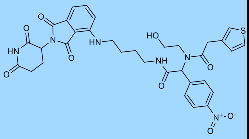	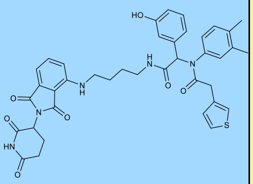	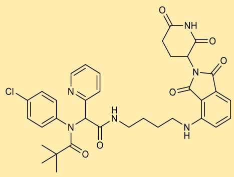
**3**	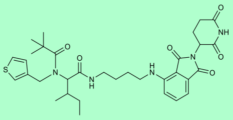	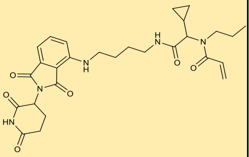	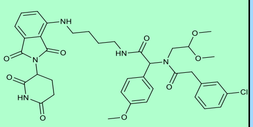	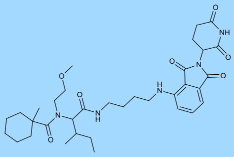
**4**	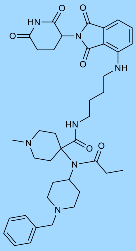	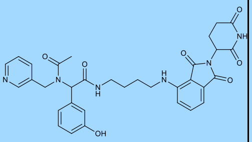	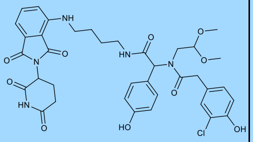	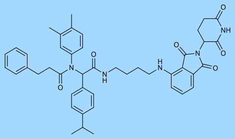
**5**	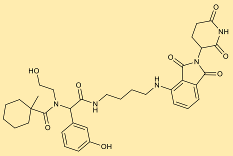	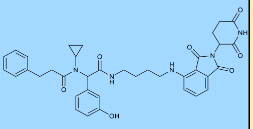	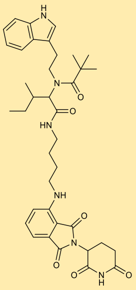	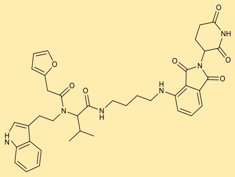

**6**	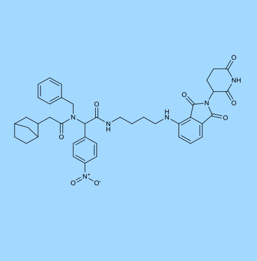	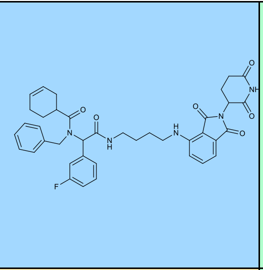	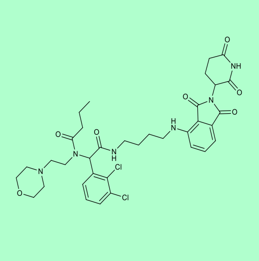	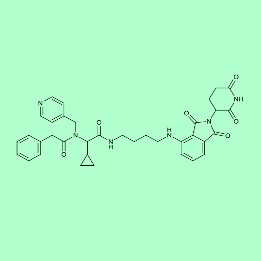
**7**	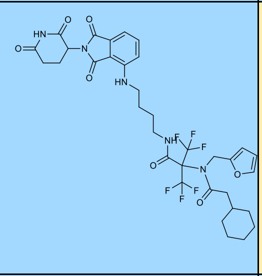	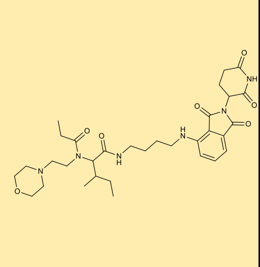	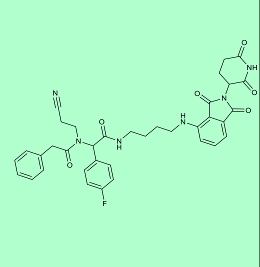	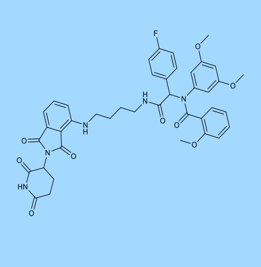
**8**	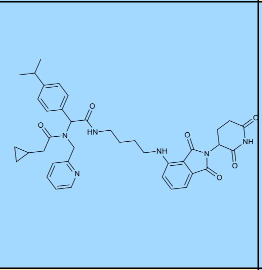	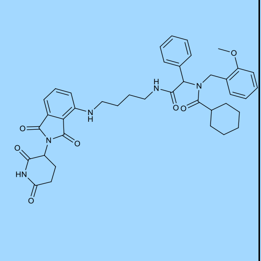	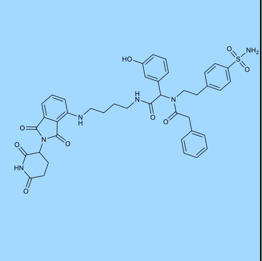	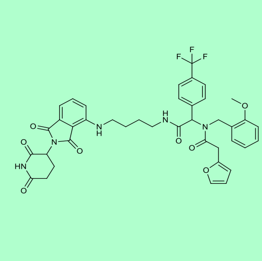
**9**	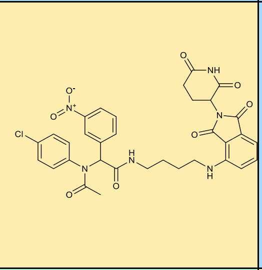	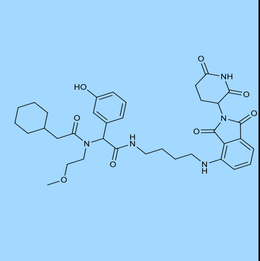	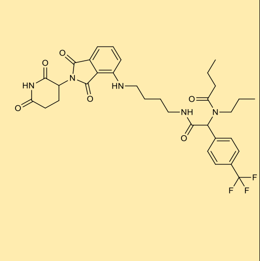	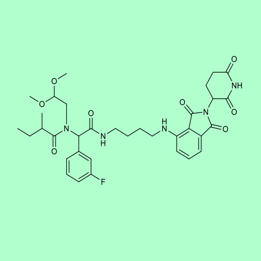
**10**	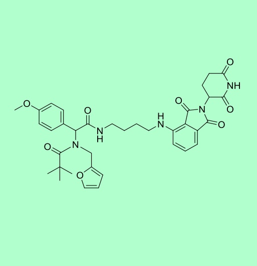	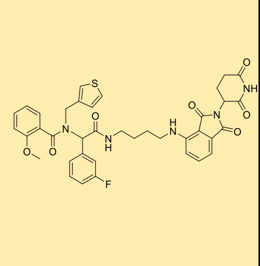	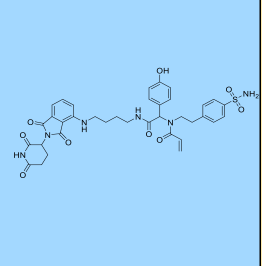	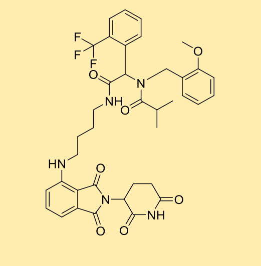
**11**	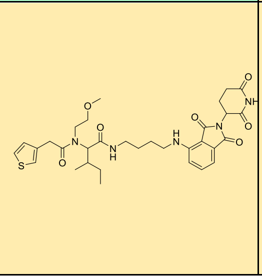	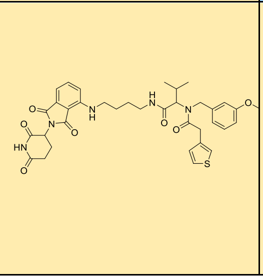	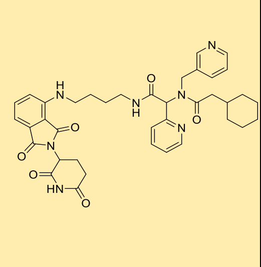	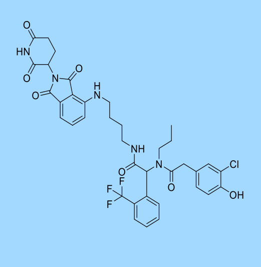

**12**	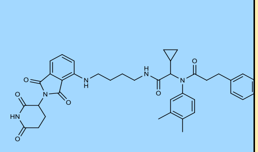	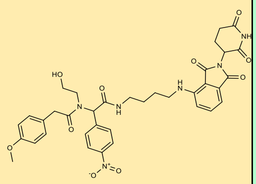	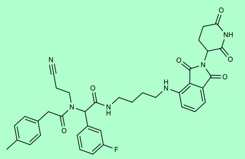	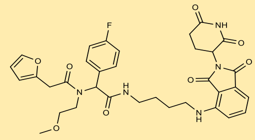
**13**	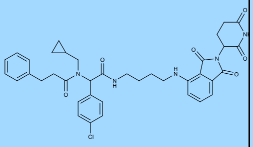	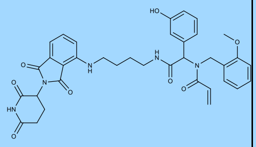	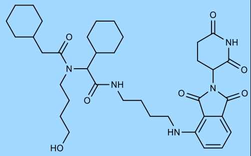	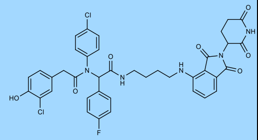
**14**	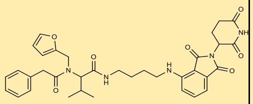	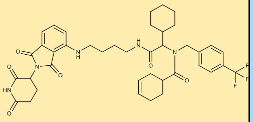	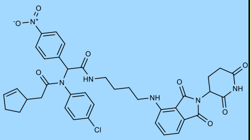	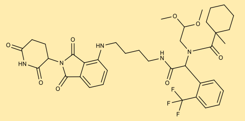
**15**	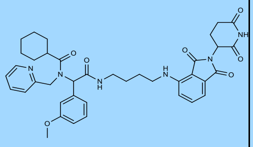	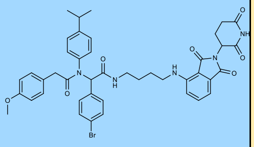	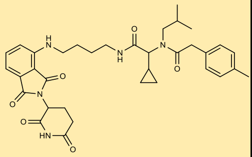	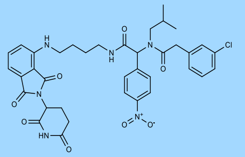
**16**	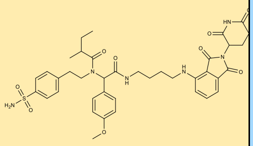	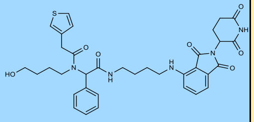	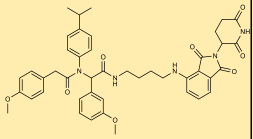	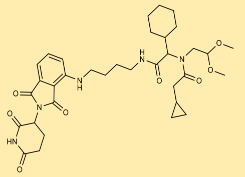
**17**	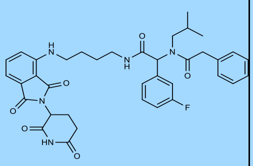	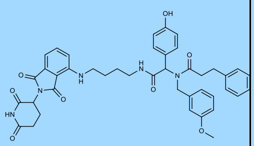	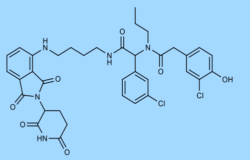	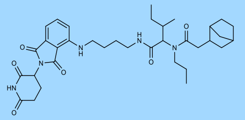

**18**	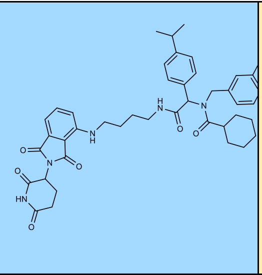	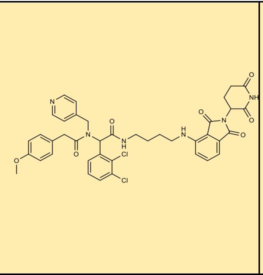	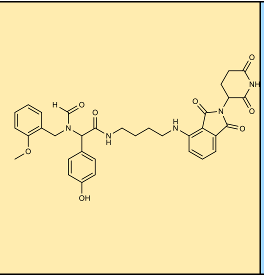	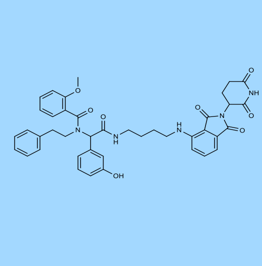
**19**	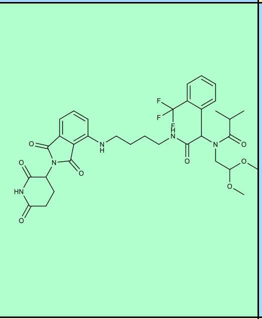	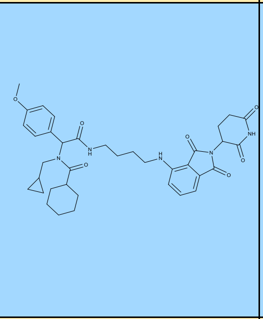	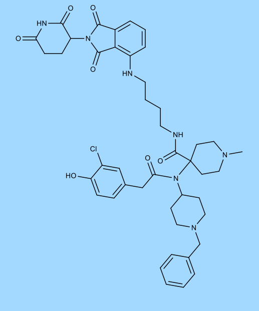	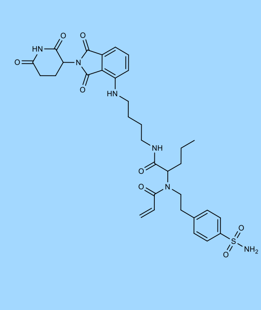
**20**	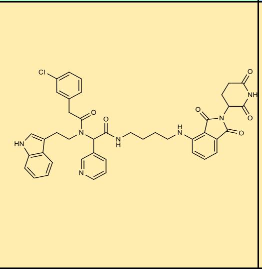	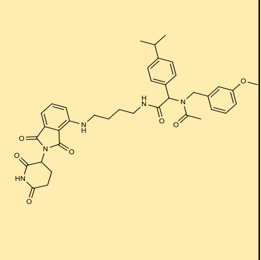	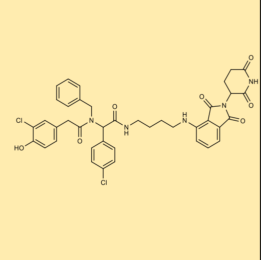	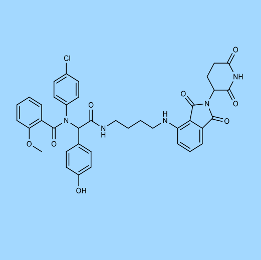
**21**	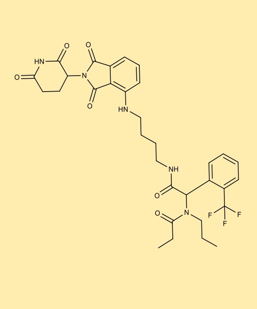	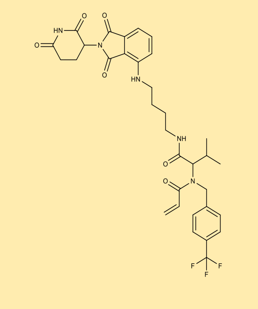	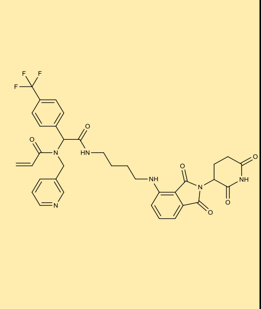	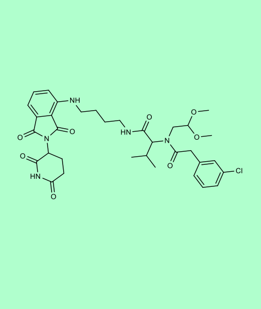
**22**	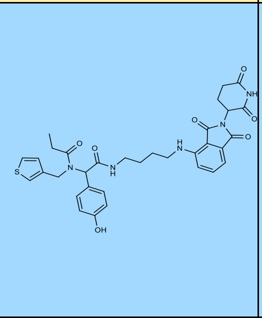	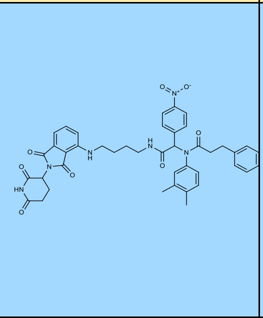	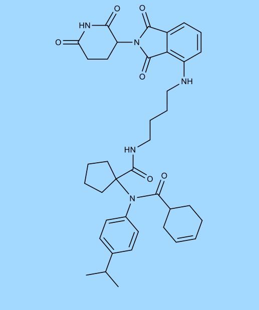	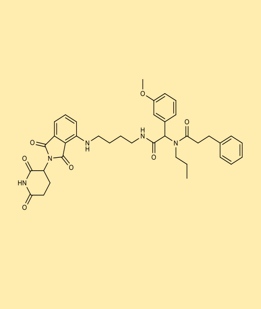
**23**	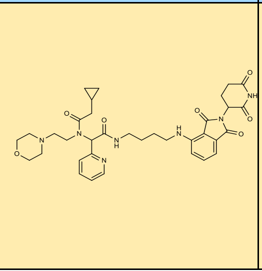	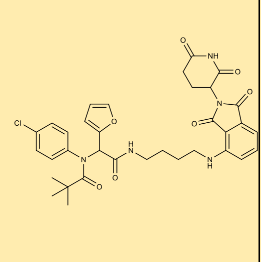	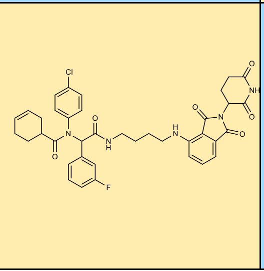	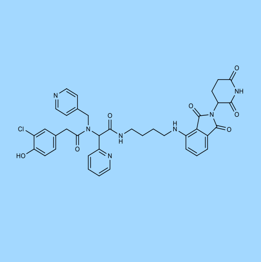

**24**	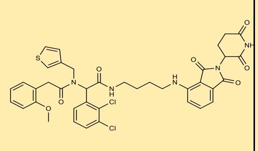	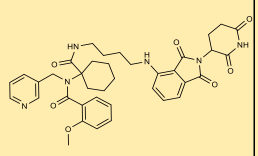	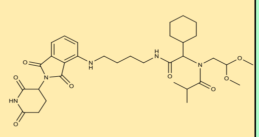	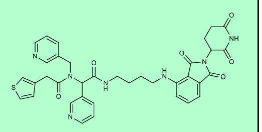

	E	F	G	H
**1**	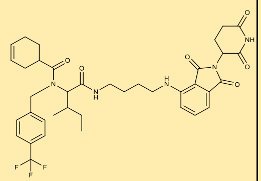	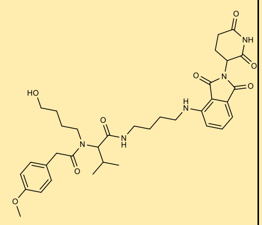	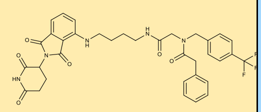	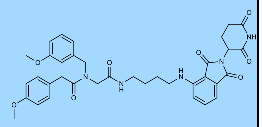
**2**	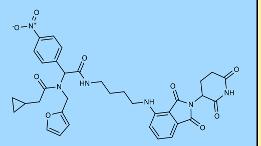	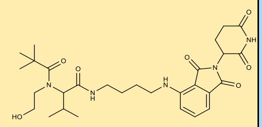	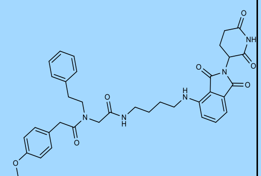	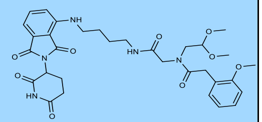
**3**	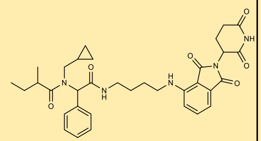	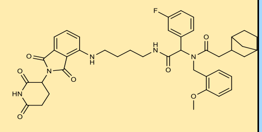	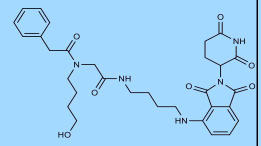	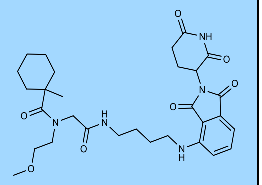
**4**	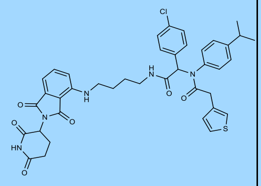	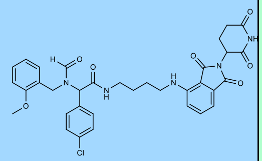	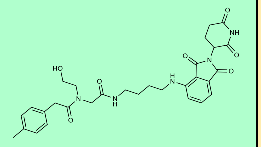	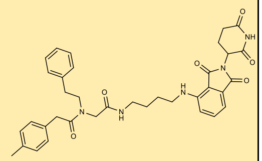
**5**	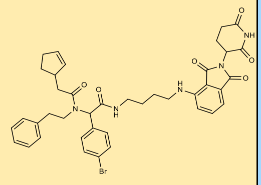	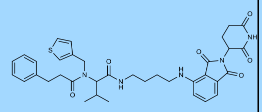	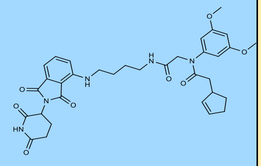	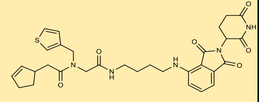
**6**	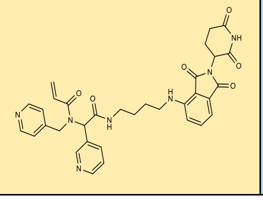	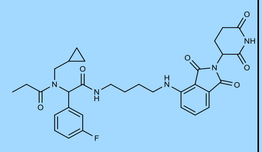	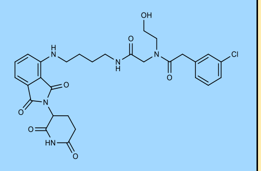	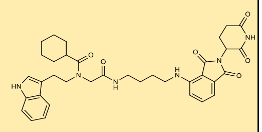

**7**	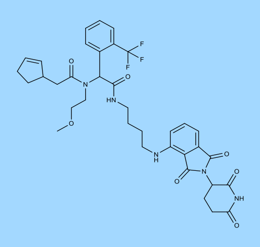	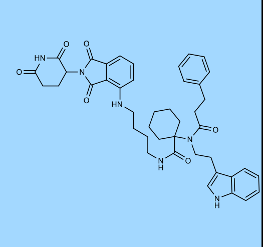	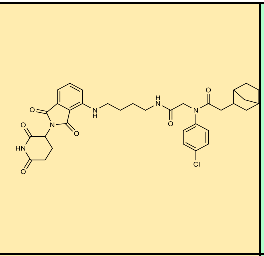	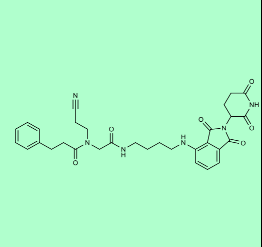
**8**	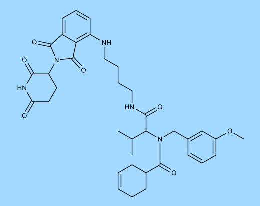	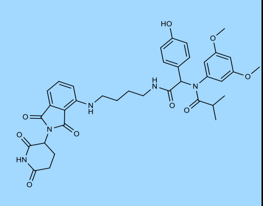	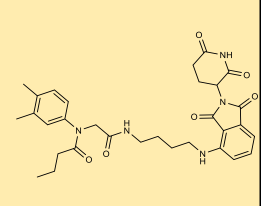	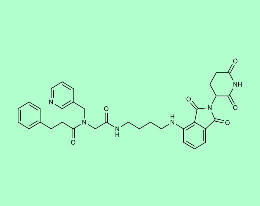
**9**	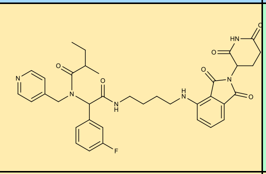	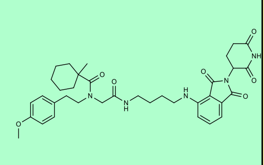	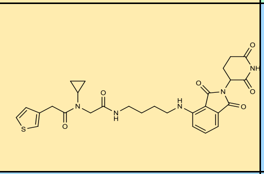	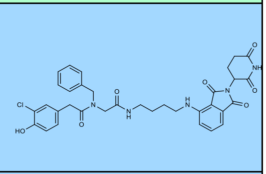
**10**	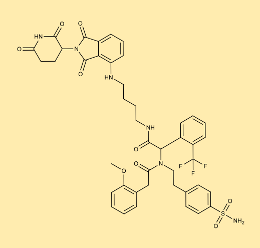	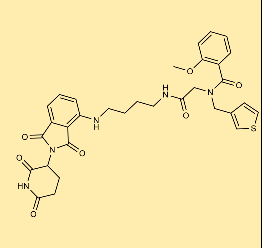	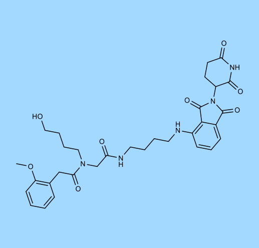	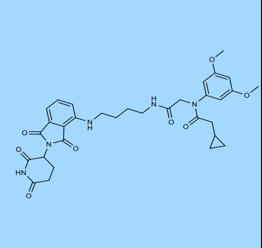
**11**	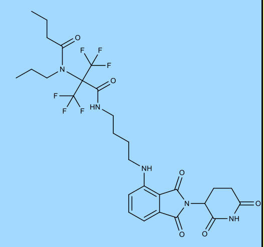	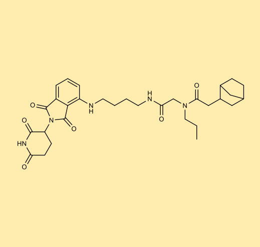	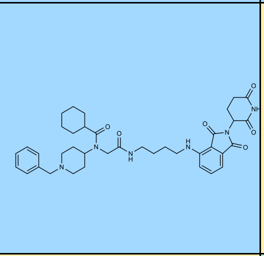	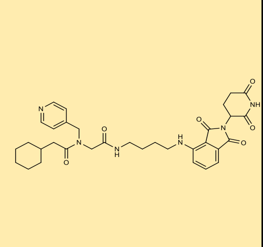
**12**	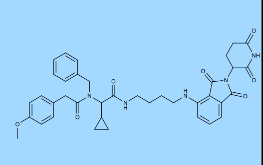	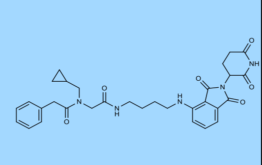	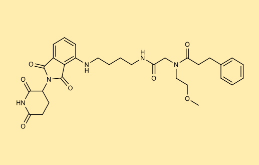	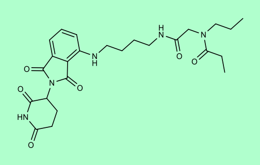
**13**	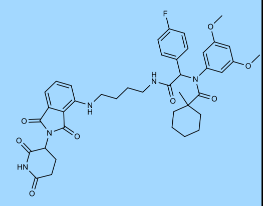	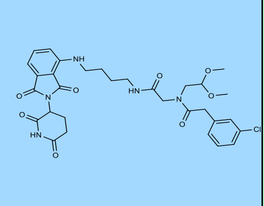	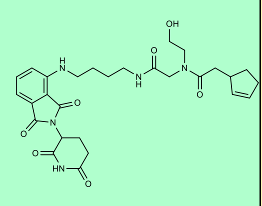	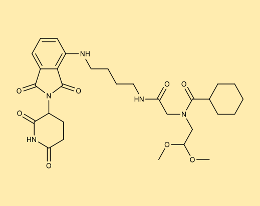
**14**	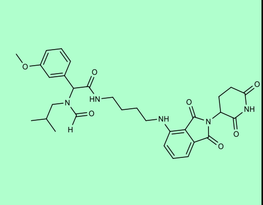	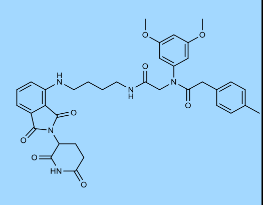	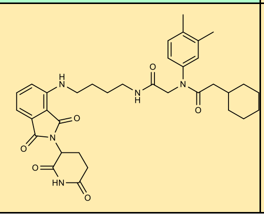	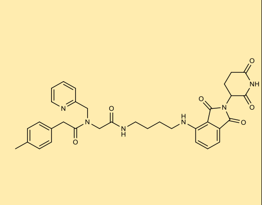

**15**	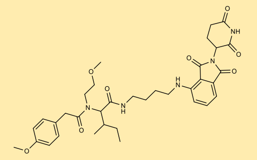	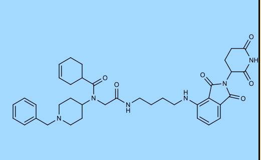	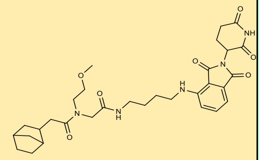	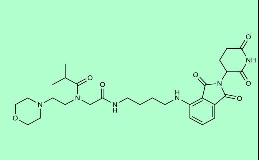
**16**	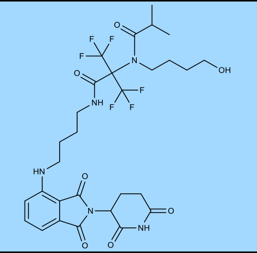	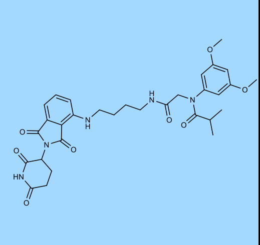	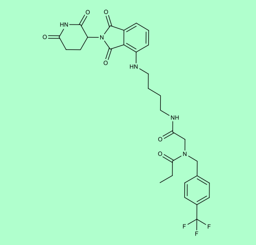	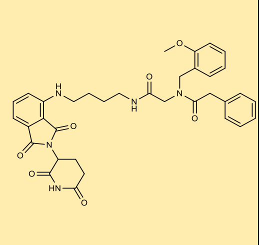
**17**	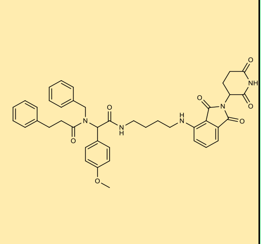	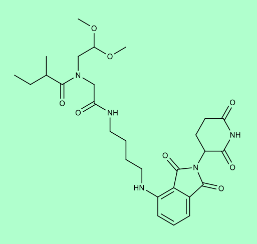	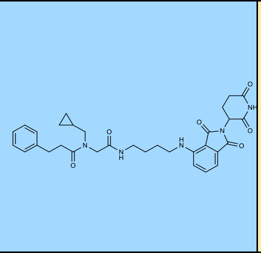	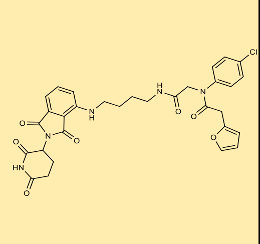
**18**	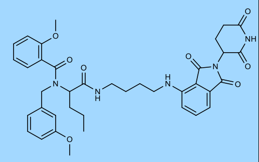	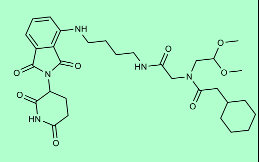	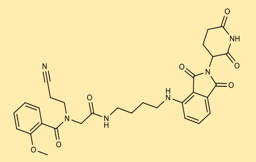	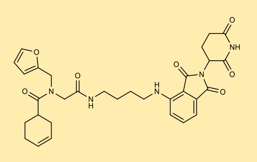
**19**	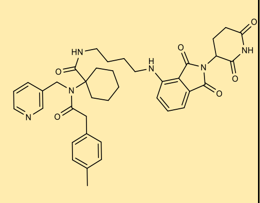	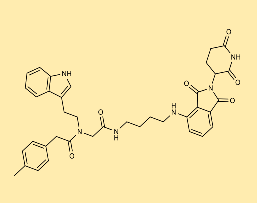	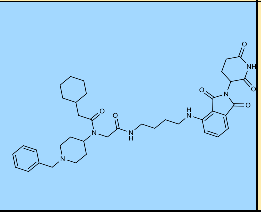	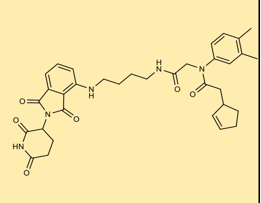
**20**	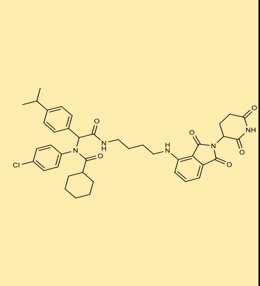	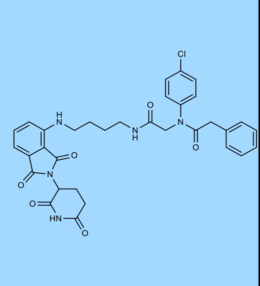	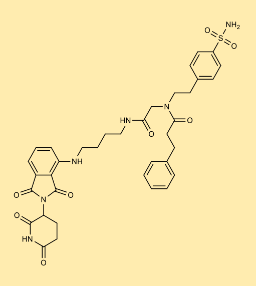	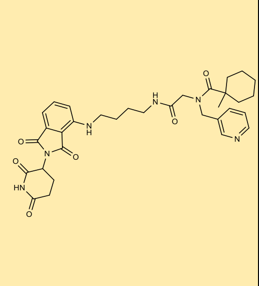
**21**	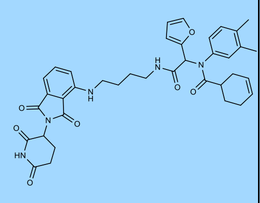	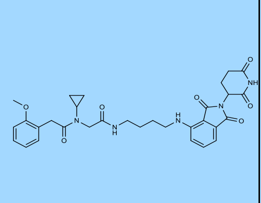	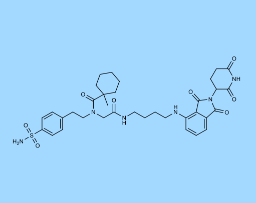	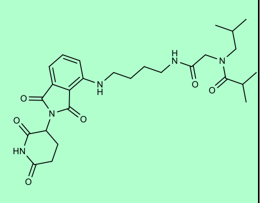
**22**	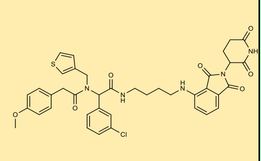	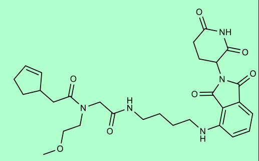	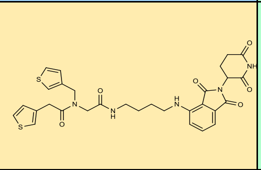	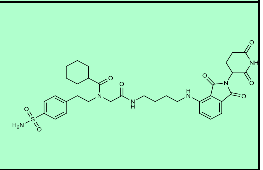

**23**	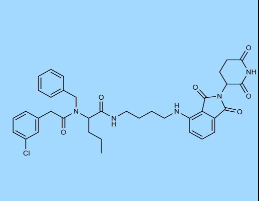	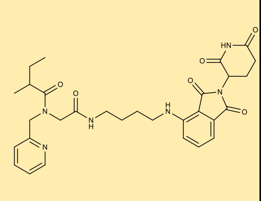	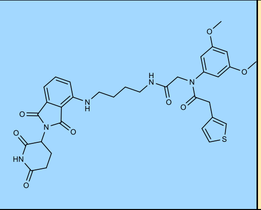	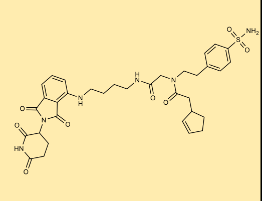
**24**	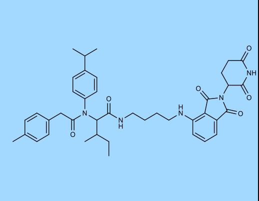	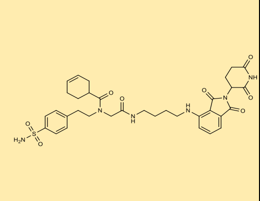	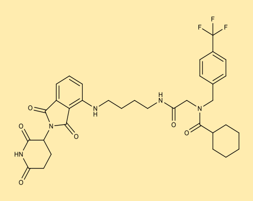	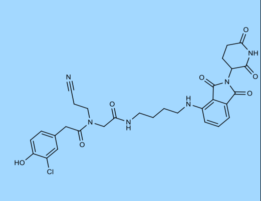

	I	J	K	L
**1**	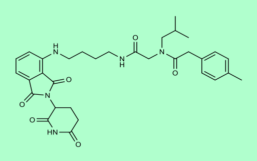	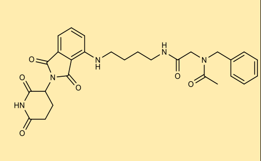	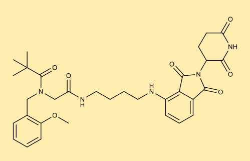	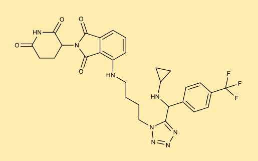
**2**	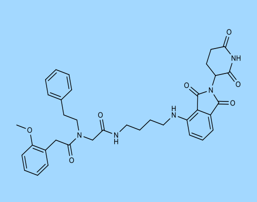	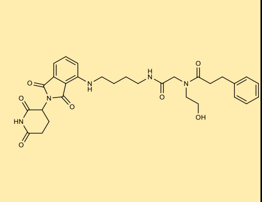	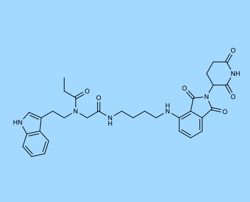	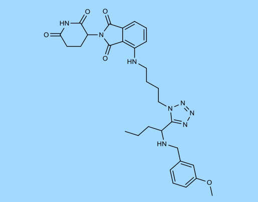
**3**	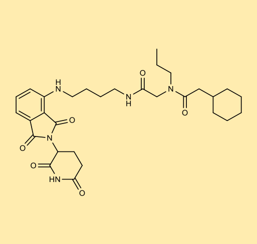	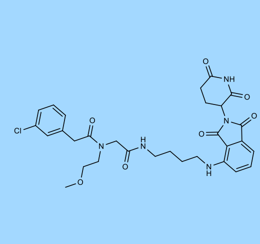	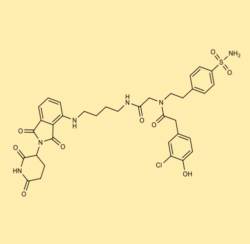	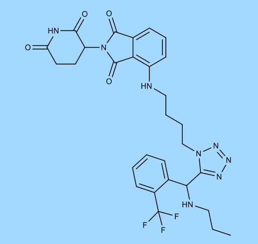
**4**	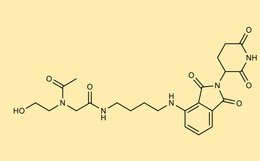	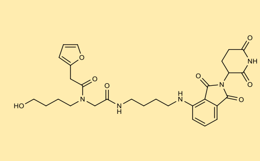	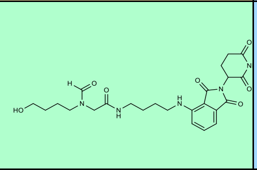	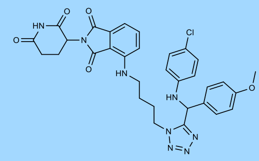
**5**	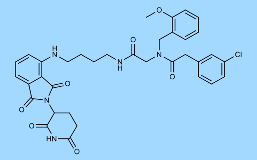	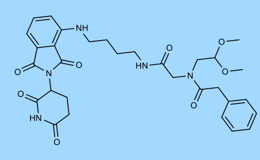	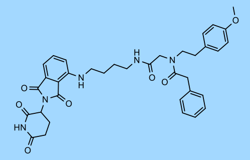	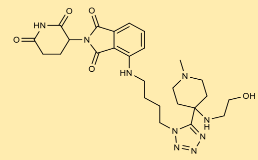
**6**	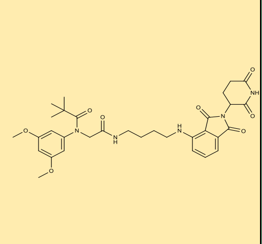	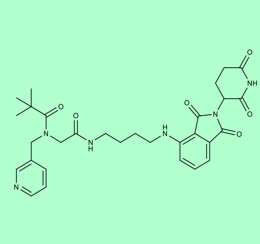	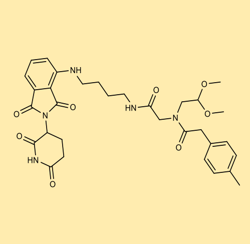	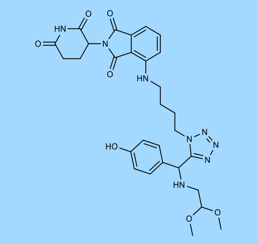

**7**	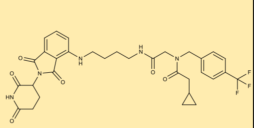	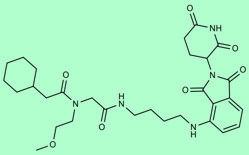	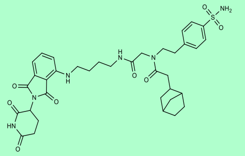	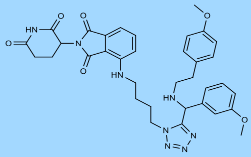
**8**	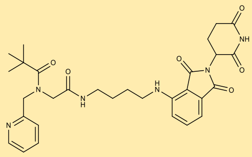	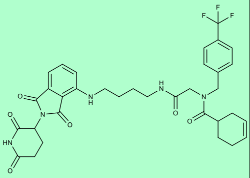	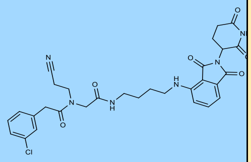	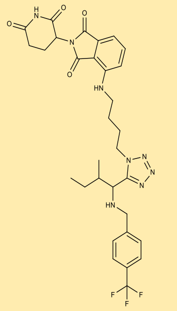
**9**	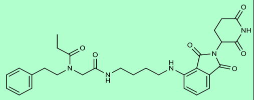	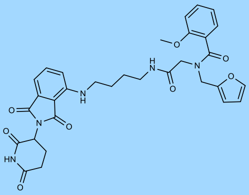	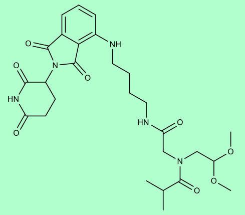	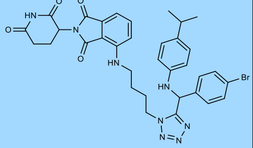
**10**	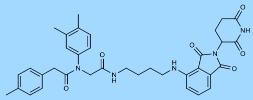	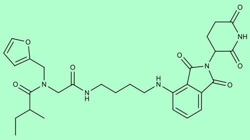	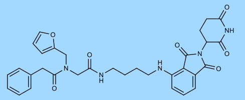	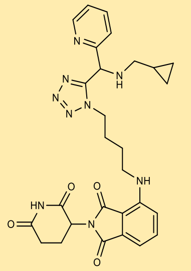
**11**	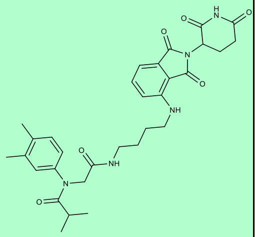	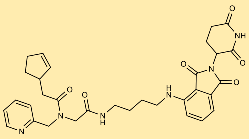	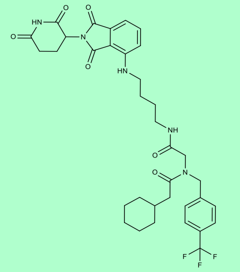	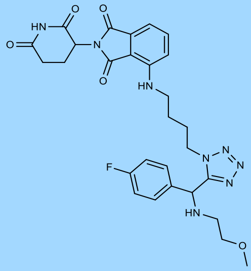
**12**	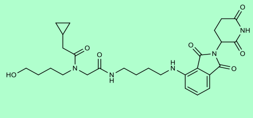	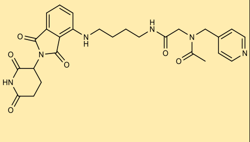	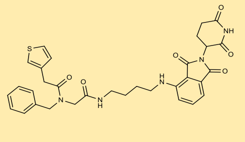	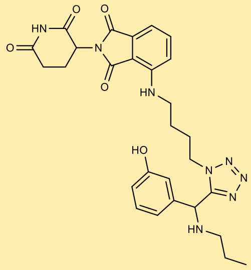

**13**	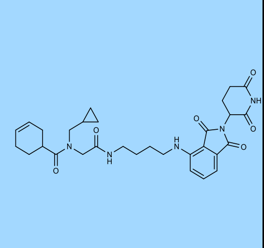	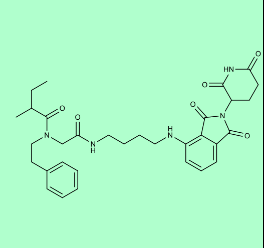	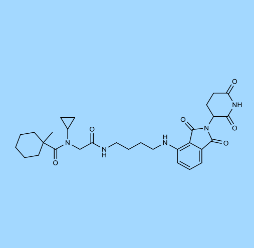	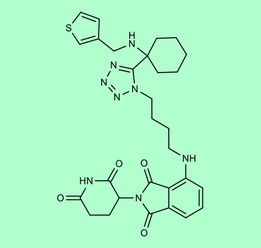
**14**	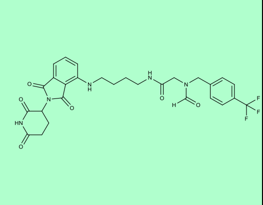	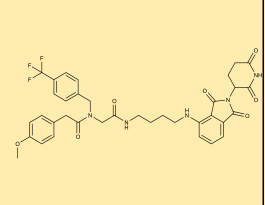	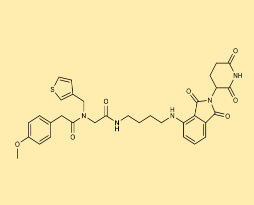	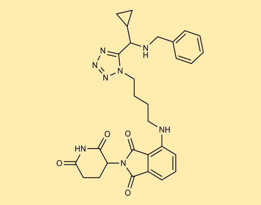
**15**	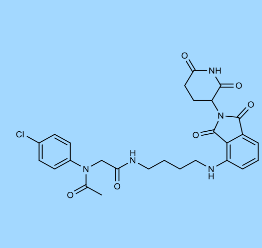	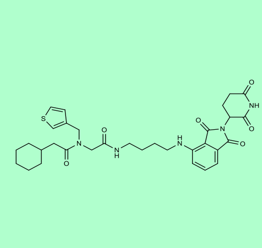	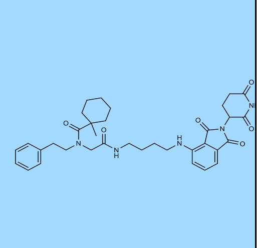	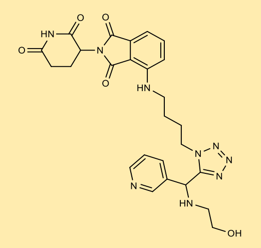
**16**	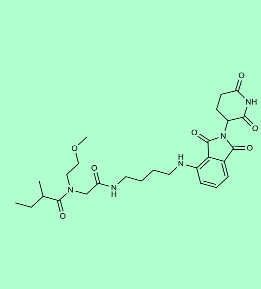	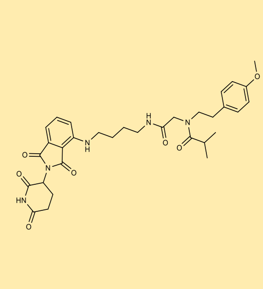	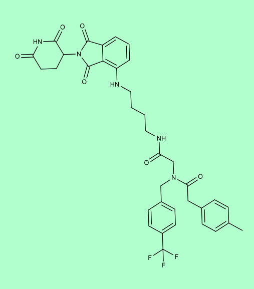	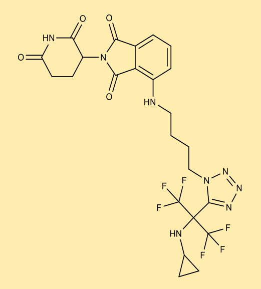
**17**	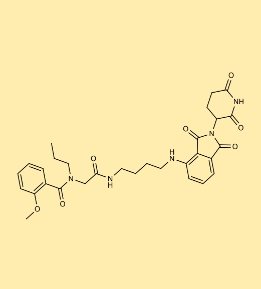	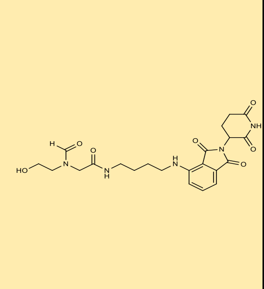	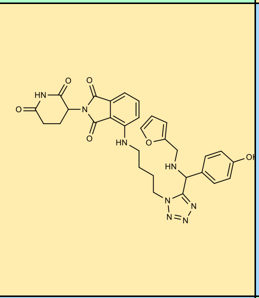	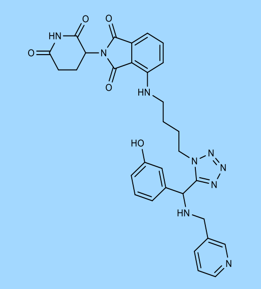
**18**	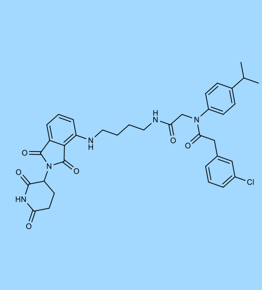	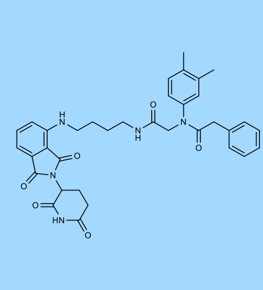	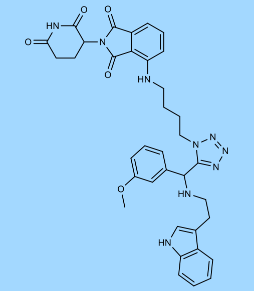	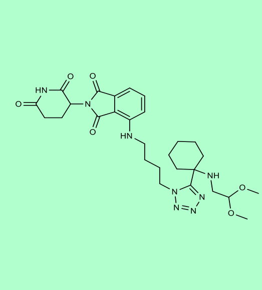
**19**	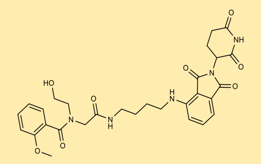	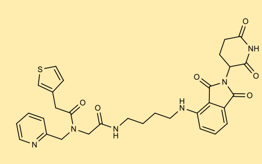	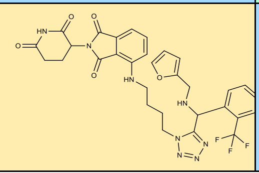	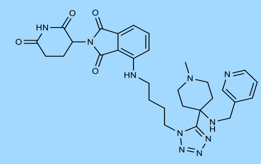

**20**	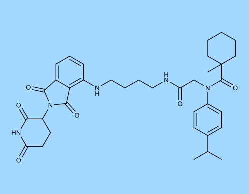	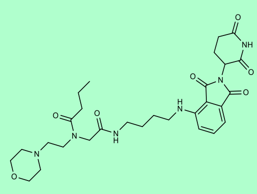	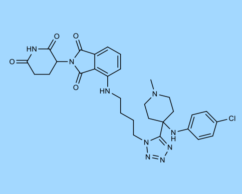	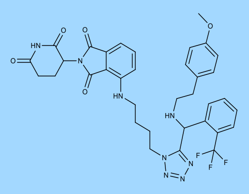
**21**	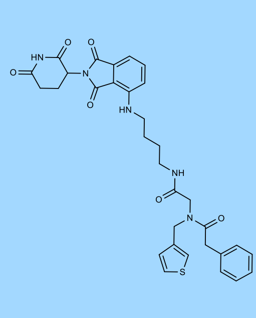	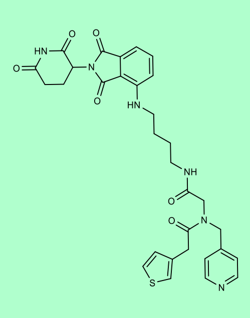	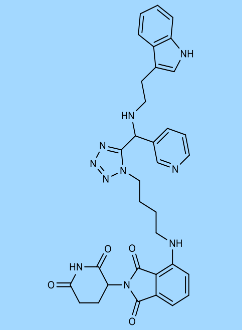	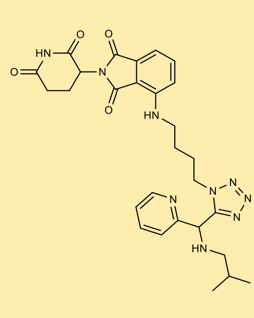
**22**	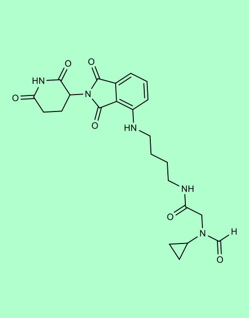	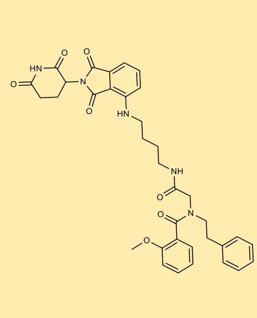	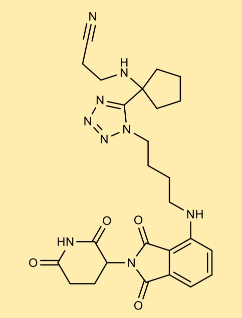	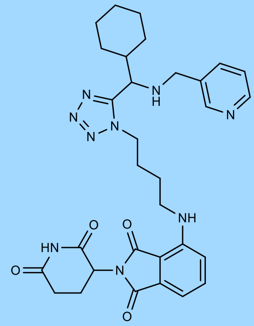
**23**	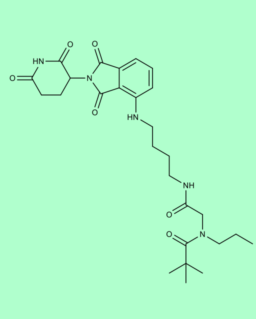	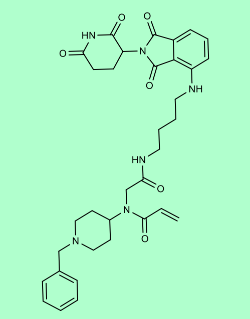	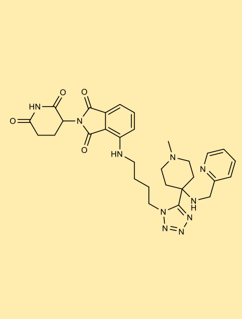	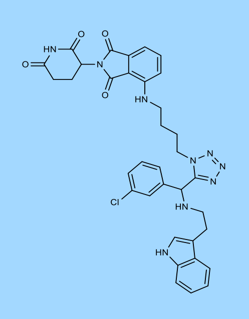
**24**	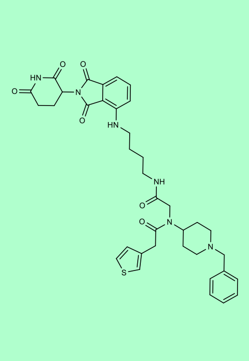	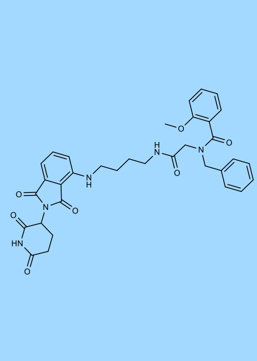	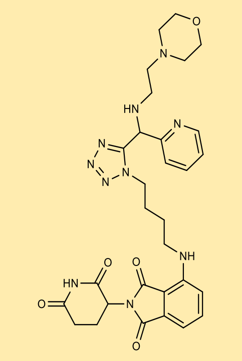	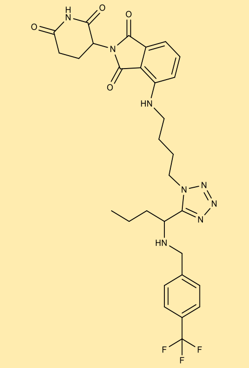
	**M**	**N**	**O**	**P**

**1**	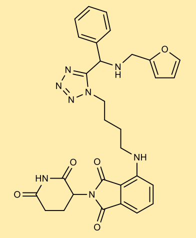	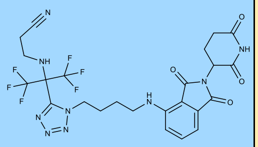	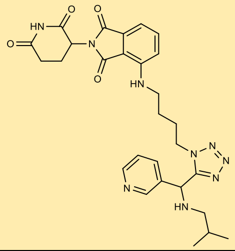	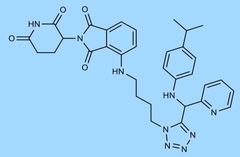
**2**	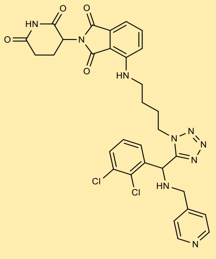	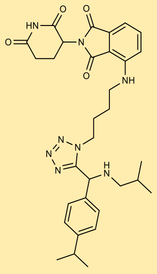	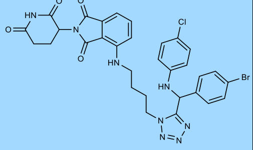	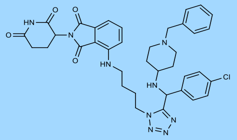
**3**	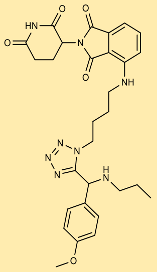	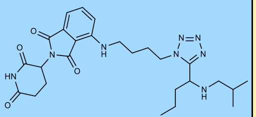	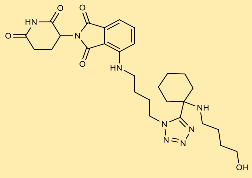	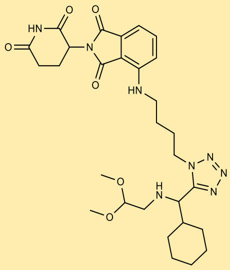
**4**	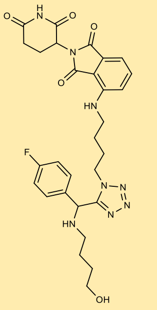	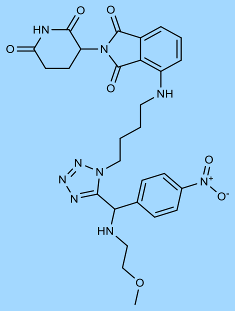	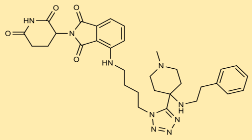	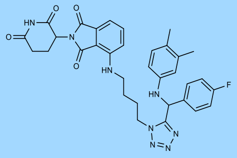
**5**	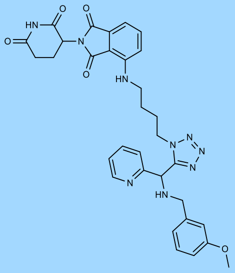	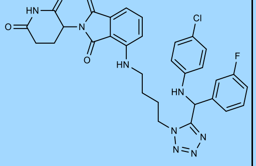	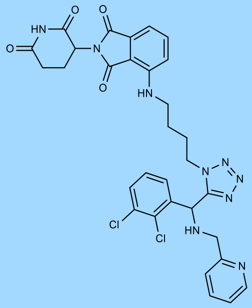	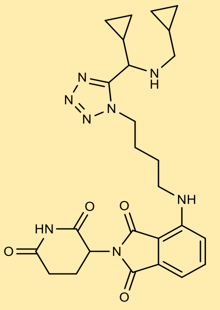
**6**	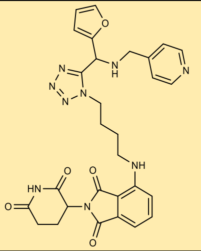	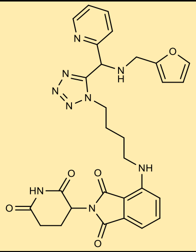	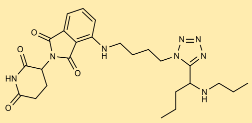	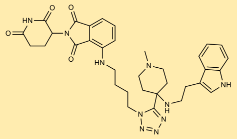

**7**	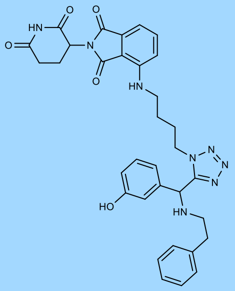	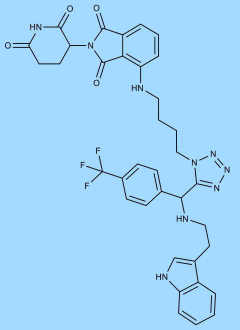	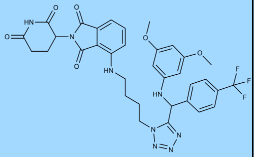	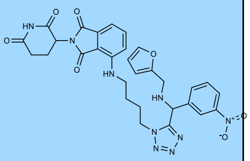
**8**	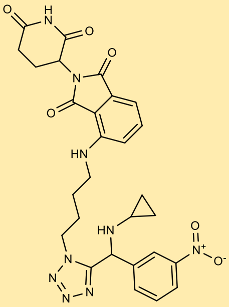	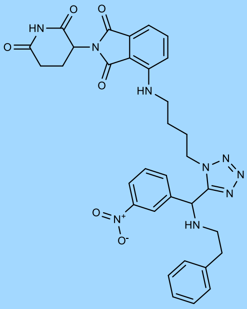	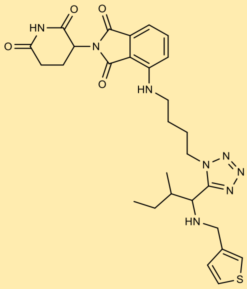	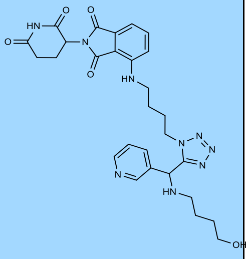
**9**	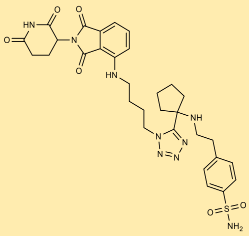	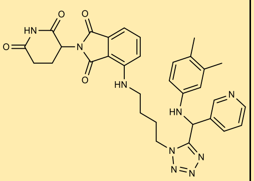	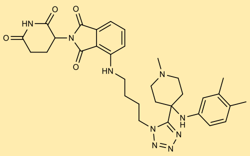	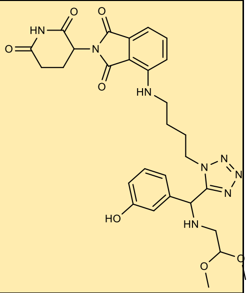
**10**	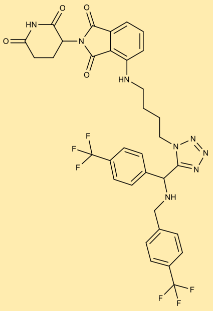	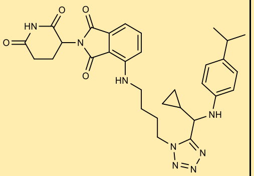	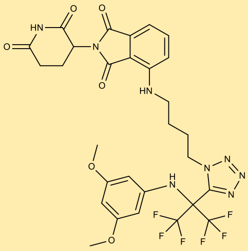	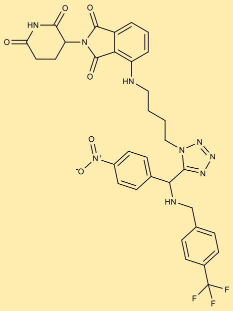
**11**	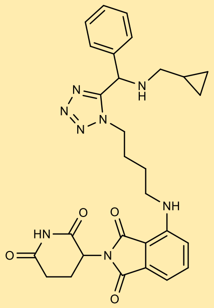	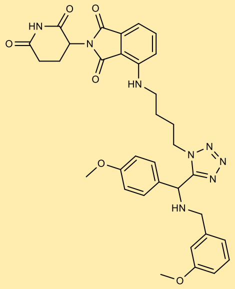	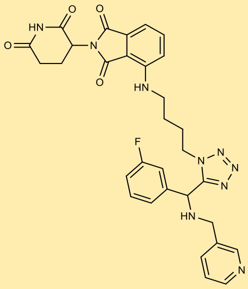	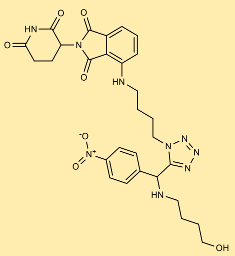

**12**	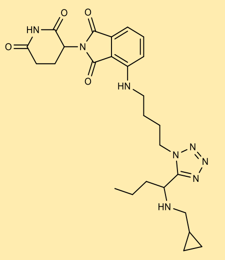	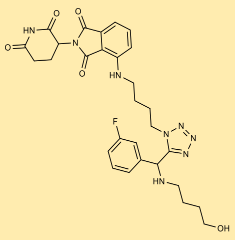	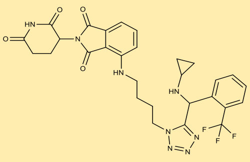	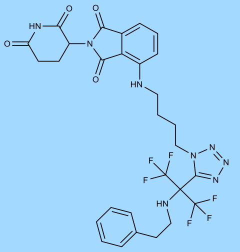
**13**	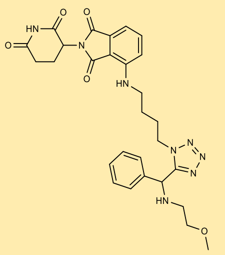	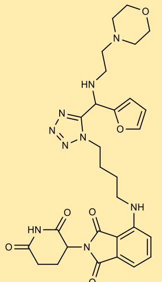	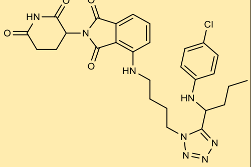	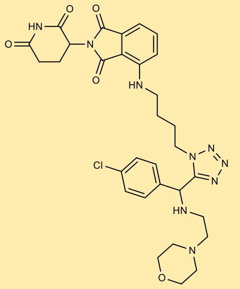
**14**	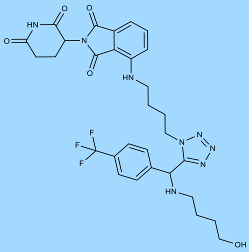	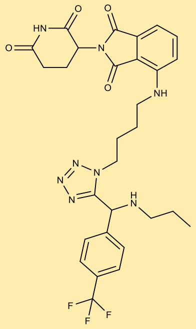	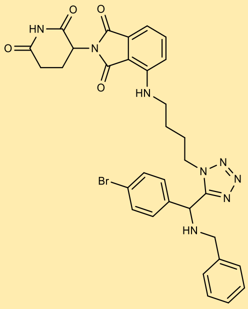	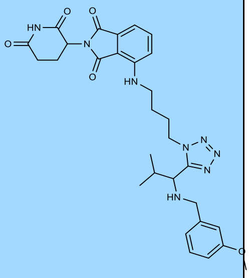
**15**	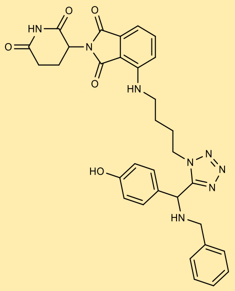	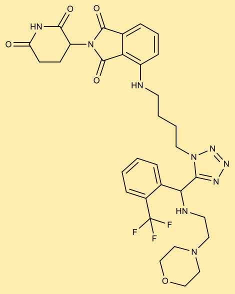	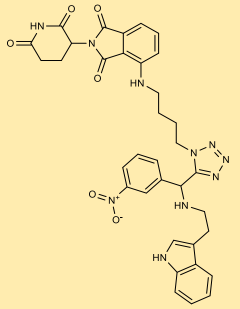	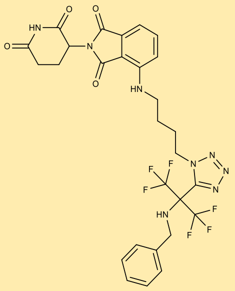
**16**	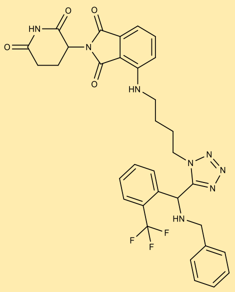	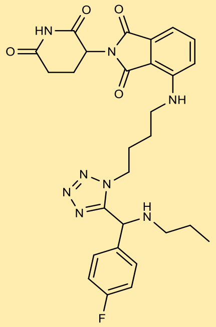	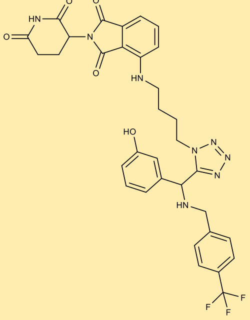	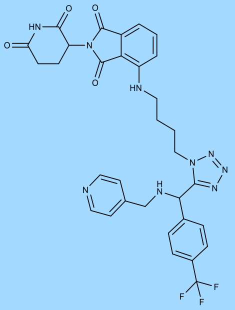

**17**	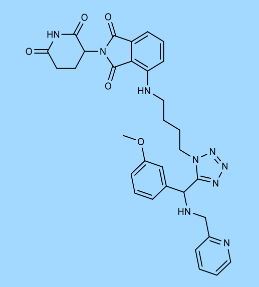	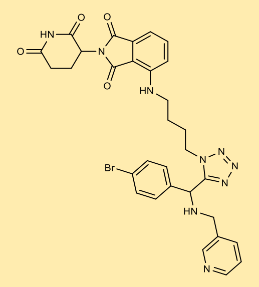	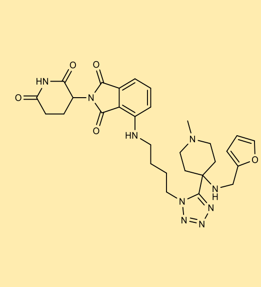	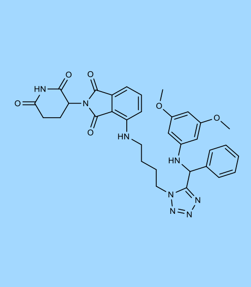
**18**	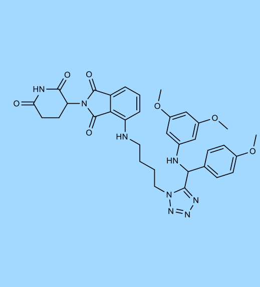	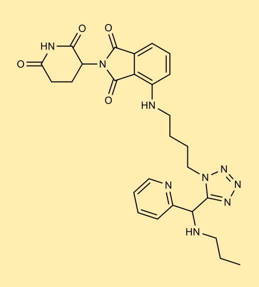	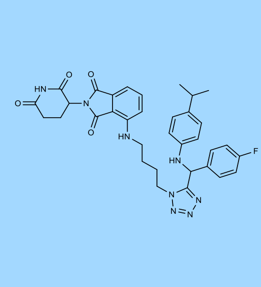	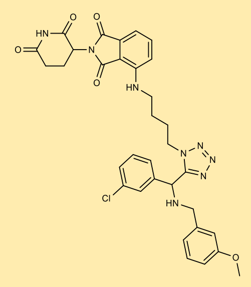
**19**	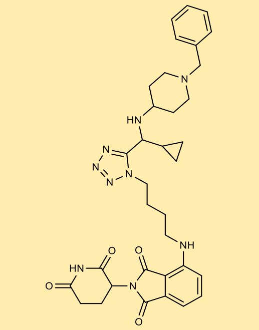	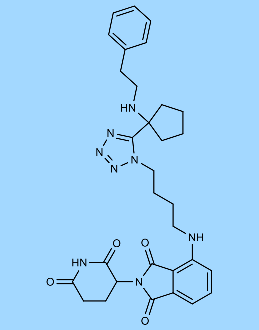	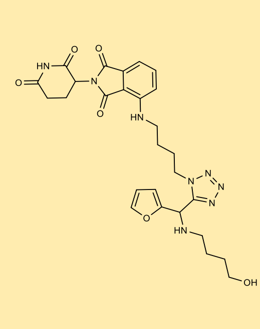	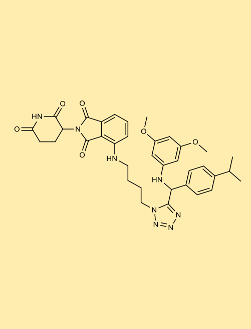
**20**	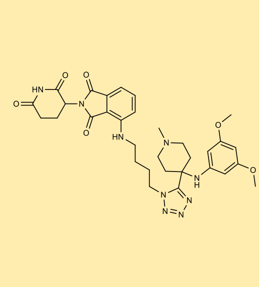	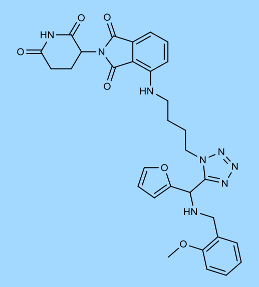	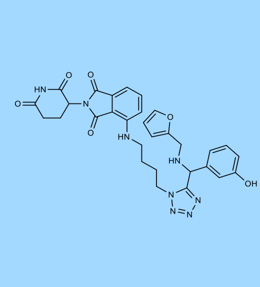	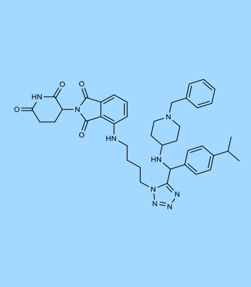
**21**	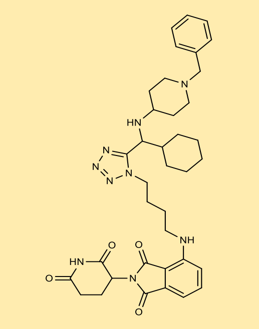	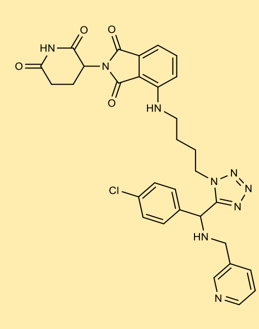	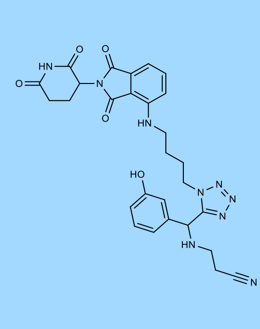	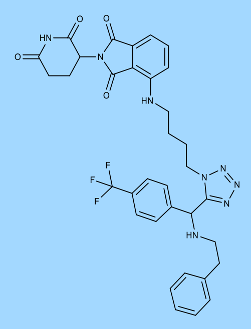
**22**	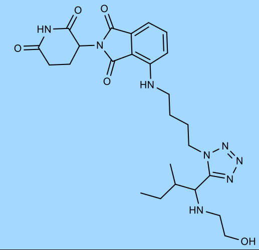	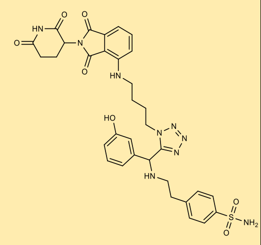	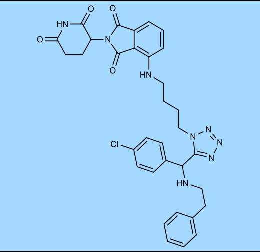	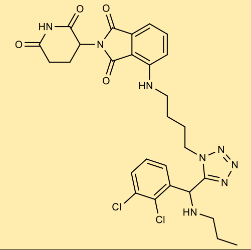

**23**	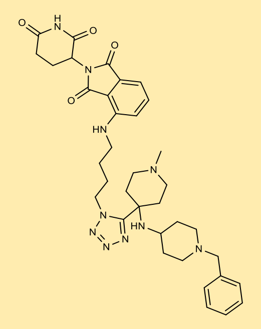	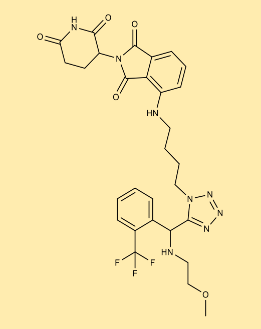	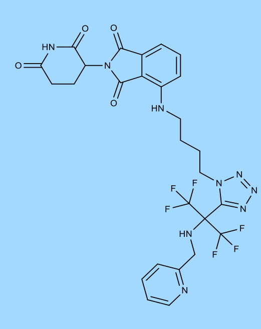	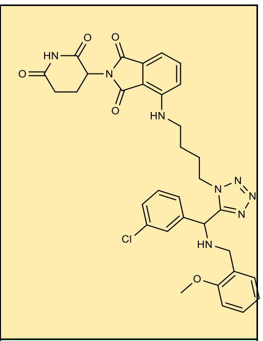
**24**	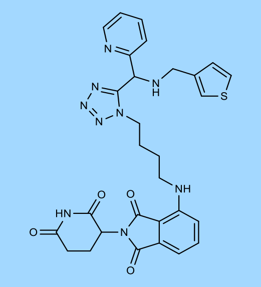	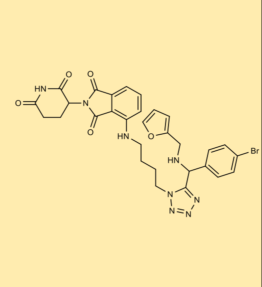	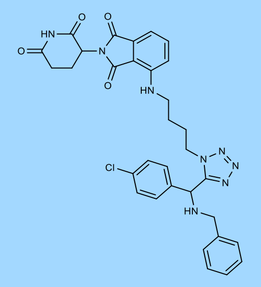	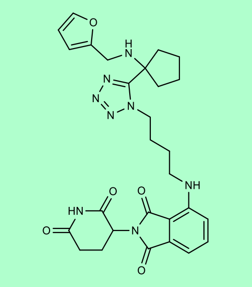

3.5 Quality Control (QC)

The analytics of all wells were performed by SFC-UV-MS. Mass spectra were measured on a Waters Investigator Supercritical Fluid Chromatograph with a 3100 MS Detector (ESI+) via flow injection analysis (FIA) and MassLynx software.

Conditions: eluent composition: MeOH, 2% H_2O, 0.1% formic acid; run time: 2 min; flow rate: 1 mL/min.

Each well of the destination plate was diluted with 30 µL ethylene glycol and then the chromatographic analysis was done by SFC-MS using an autosampler.

The SFC analytic of one well took ~2 min, resulting in an overall measuring time for the 384 wells of around 13 h.

3.6 Automated analysis of mass spectrometry data

3.6.1 Preparation

Mass spectrometry (MS) data were automatically analyzed using in-house written Python software. This software makes use of the mzXML file format that first needs to be created. For this purpose, mzXML files were converted from Waters RAW using the MSConvert tool (version 3.0) from the ProteoWizard project using the default settings. These resulting files were consecutively used in the in-house written program, which is documented in an online repository (https://bitbucket.org/ca_warmerdam/auto-ms-analysis). In addition to the mass spectrometry files, a txt file with the smiles and the location of the expected product per well on the plate were entered. These locations are used both to create an output matrix and as an identifier for matching the expected product to an mzXML file. The expected time range for the bulk of the peaks was set to 0.1-1 min. The expected M/Z range that was specified as 350-

900 corresponds to the range the mass spectrometer was set to detect (only E10 well was specified as 350-1000). For running the software Python 3.6.6 was used with the additional packages which were in concordance with the requirements specified within the repository.

3.6.2 Processing mzXML files into spectra

Within the python software, mzXML files are first parsed into a queryable data structure using the Python XML parser module. Next, the MS data, comprised of scans that together represent the spectrum, are filtered in order to remove uninformative scans that are labelled with an msLevel of 0. In addition, scans that are outside of the specified time range are discarded. The contents of the scans are subsequently decoded as these are Base64 encoded by default, and the resulting values are thereafter decompressed using the decompression functionality within the zlib Python module. This results in a regular collection of pairs consisting of a mass-to-charge ratio with corresponding intensities. Intensity values were considered erroneous, and are thus removed, if they expand further than 5000 times the inter quantile range of the intensities within the specific well. Afterwards, the mass-to-charge ratios are collected in bins of size 1 around an integer value. Within this process, the intensity values are summed for every bin.

3.6.3 Prediction procedure

To assign a prediction of abundance for a product, from the peaks corresponding to all user specified adduct masses (M+H, M+Na, M+K peaks) (± 0.5), the highest peak within a well is isolated. The intensity of this peak relative to the highest peak represents the initial prediction for a product. After having run the software, predictions with values more than 0.9 were classified as green, predictions with values less than 0.1 were classified as blue, and predictions with values equal and between these thresholds were classified as yellow.

3.6.4 Python code

Please refer to our previous publication.[2]

Examples of SFC-MS analytics directly out of the 384-well plate

G7 (Yellow)

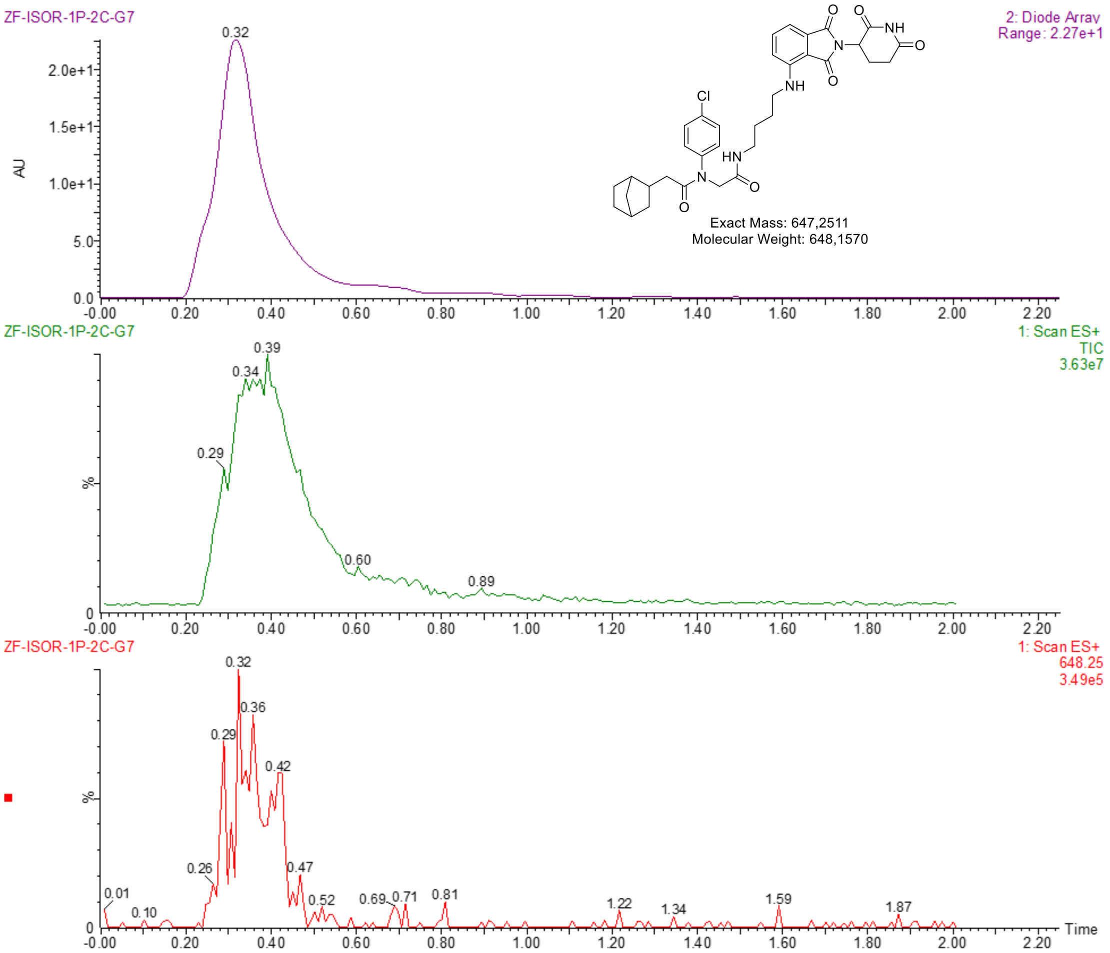

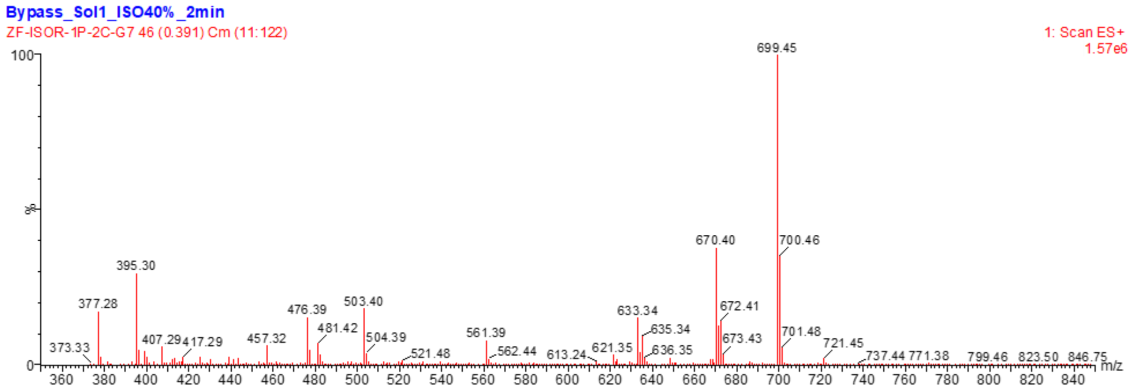

H7 (Green)

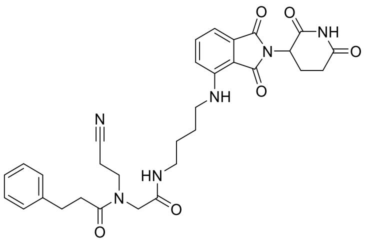

Exact Mass: 586,2540
Molecular Weight: 586,6490

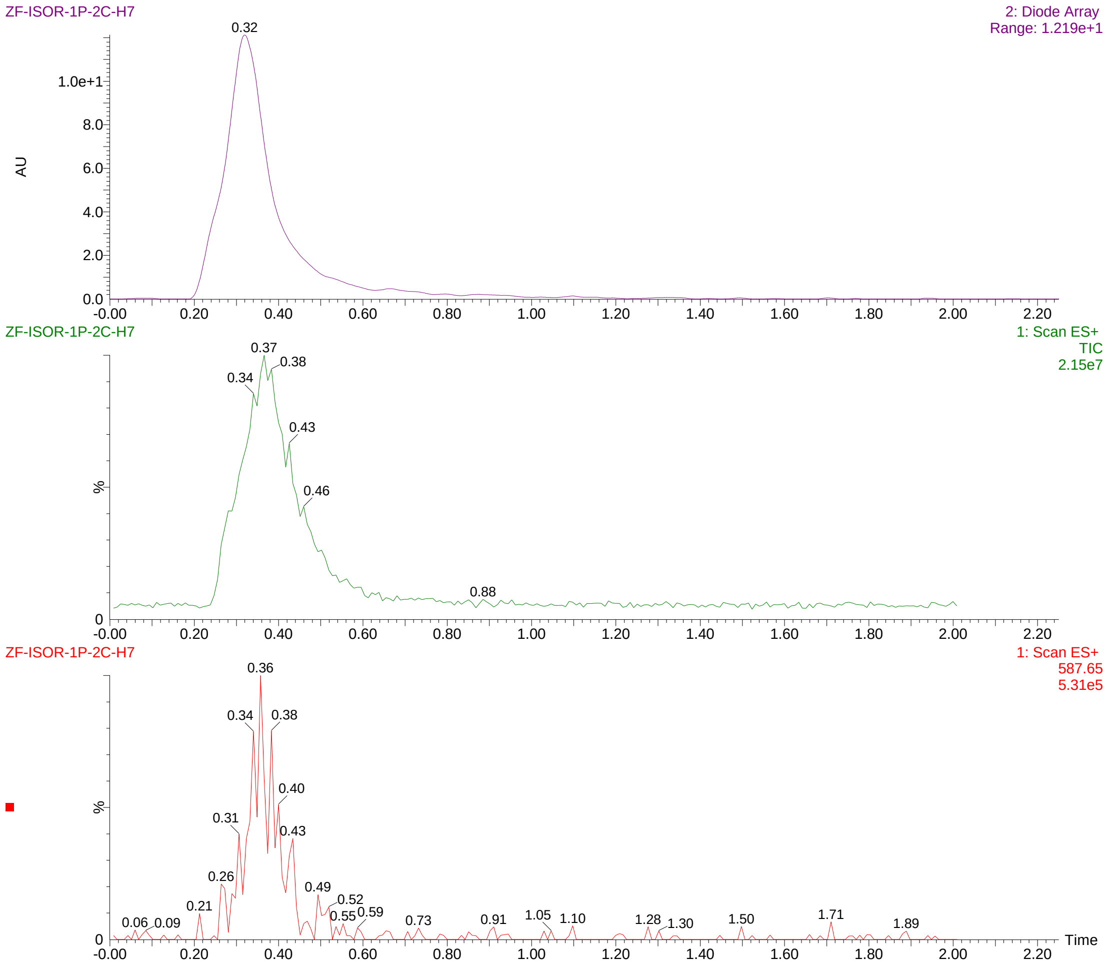

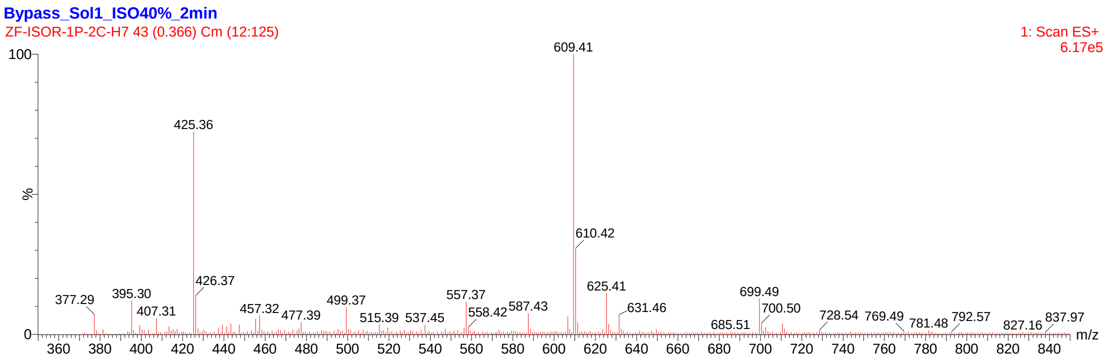

J7 (Green)

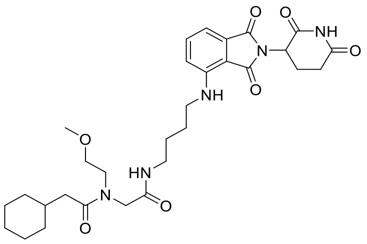

Exact Mass: 583,3006
Molecular Weight: 583,6860

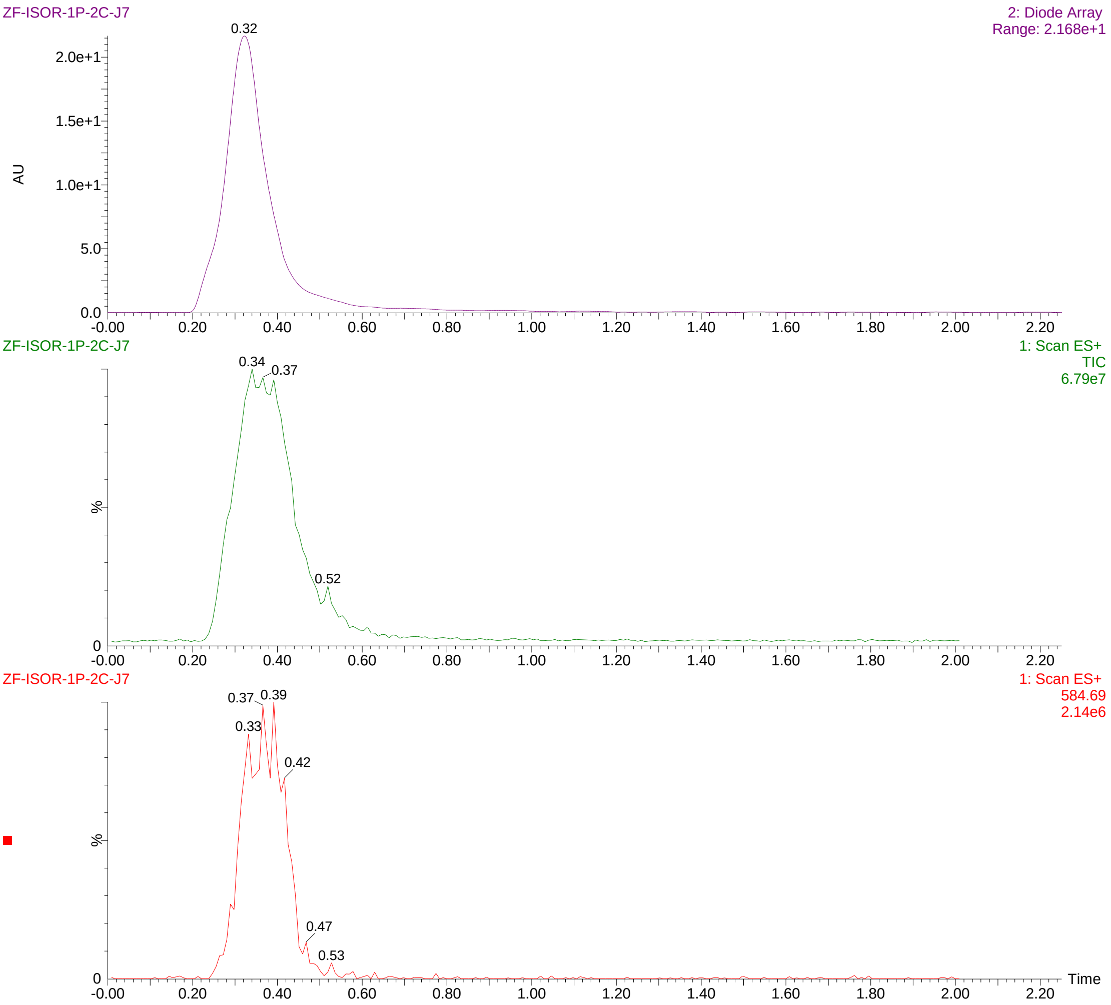

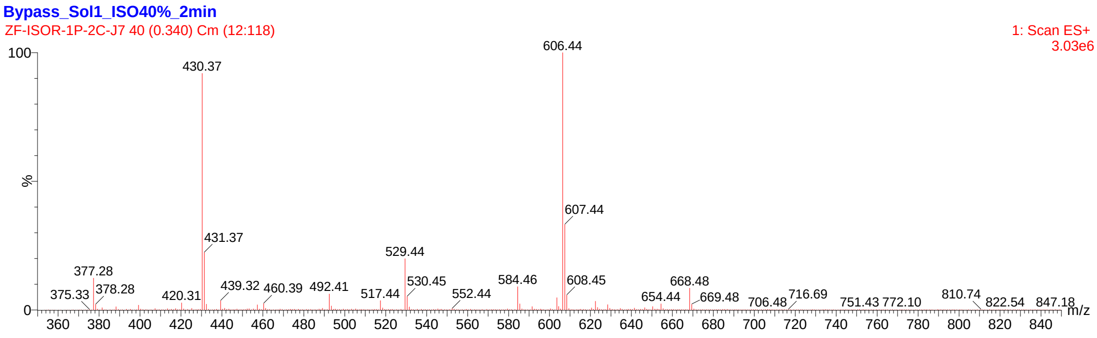

K7 (Green)

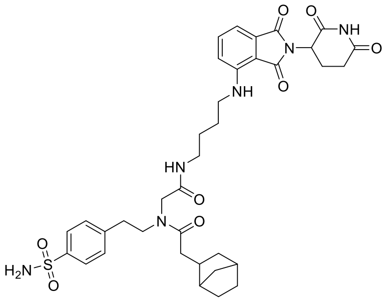

Exact Mass: 720,2941
Molecular Weight: 720,8420

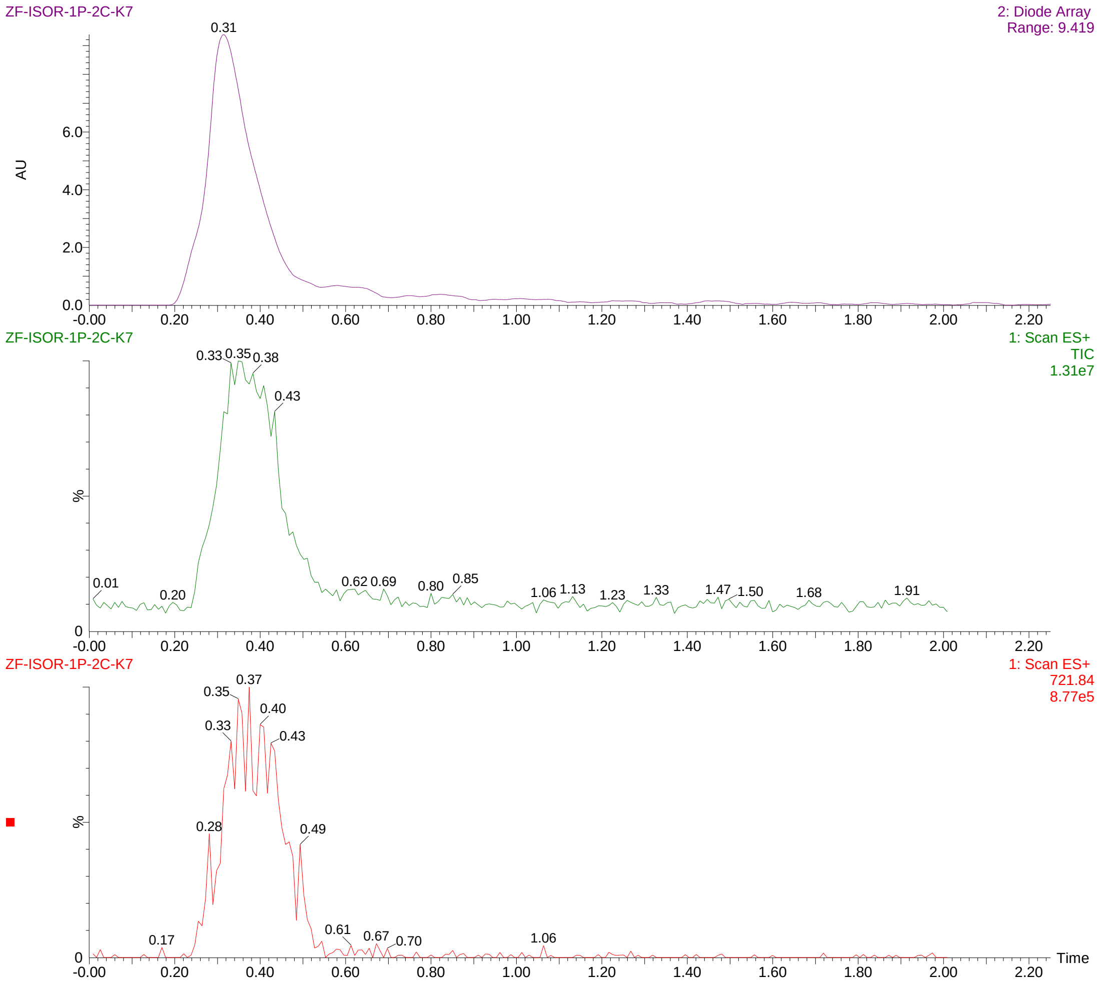

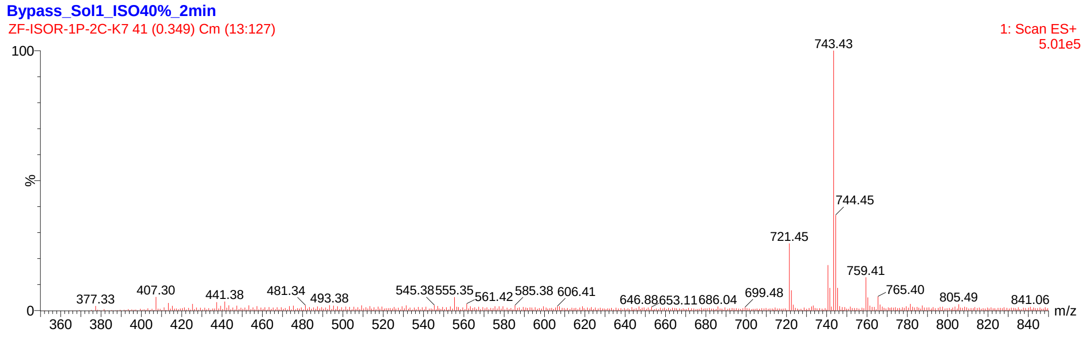

C9 (Yellow)

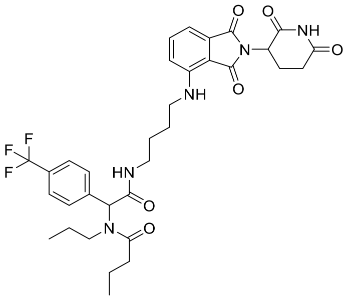

Exact Mass: 657,2774
Molecular Weight: 657,6912

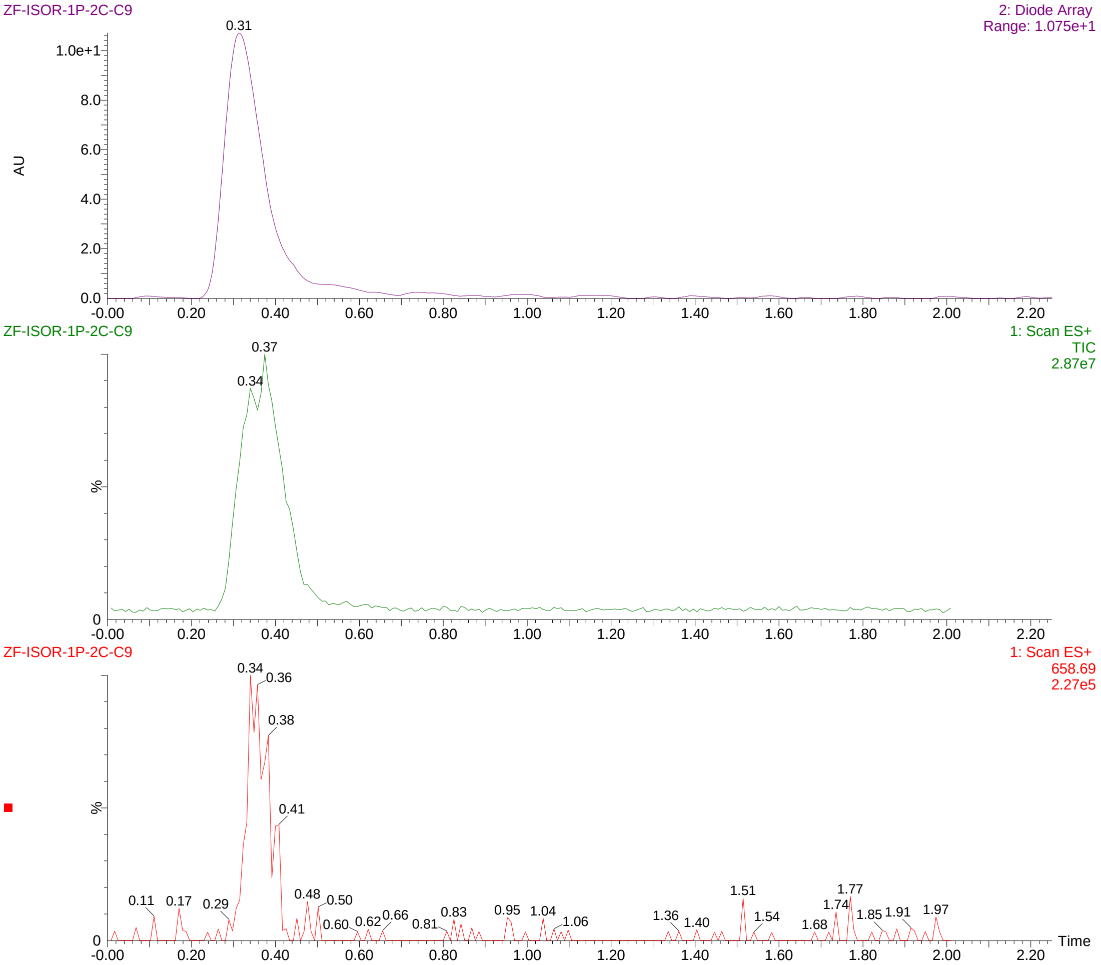

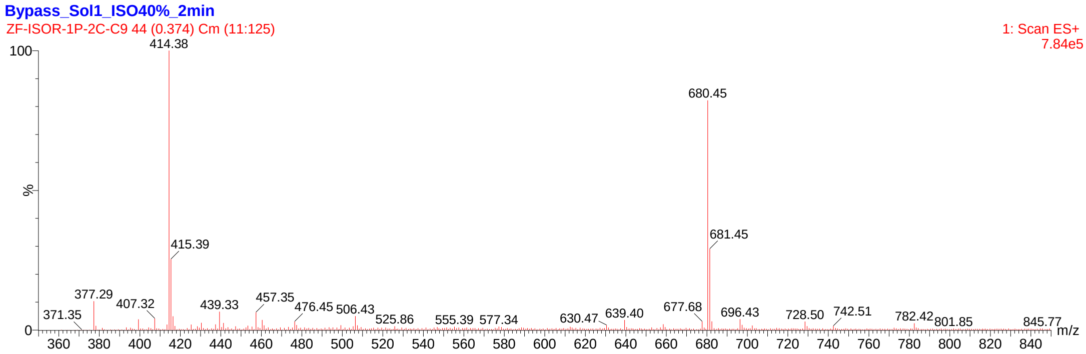

F9 (Green)

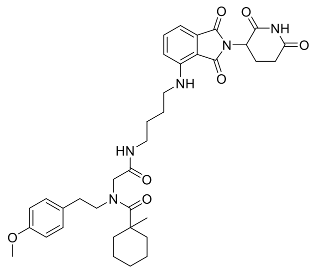

Exact Mass: 659,3319
Molecular Weight: 659,7840

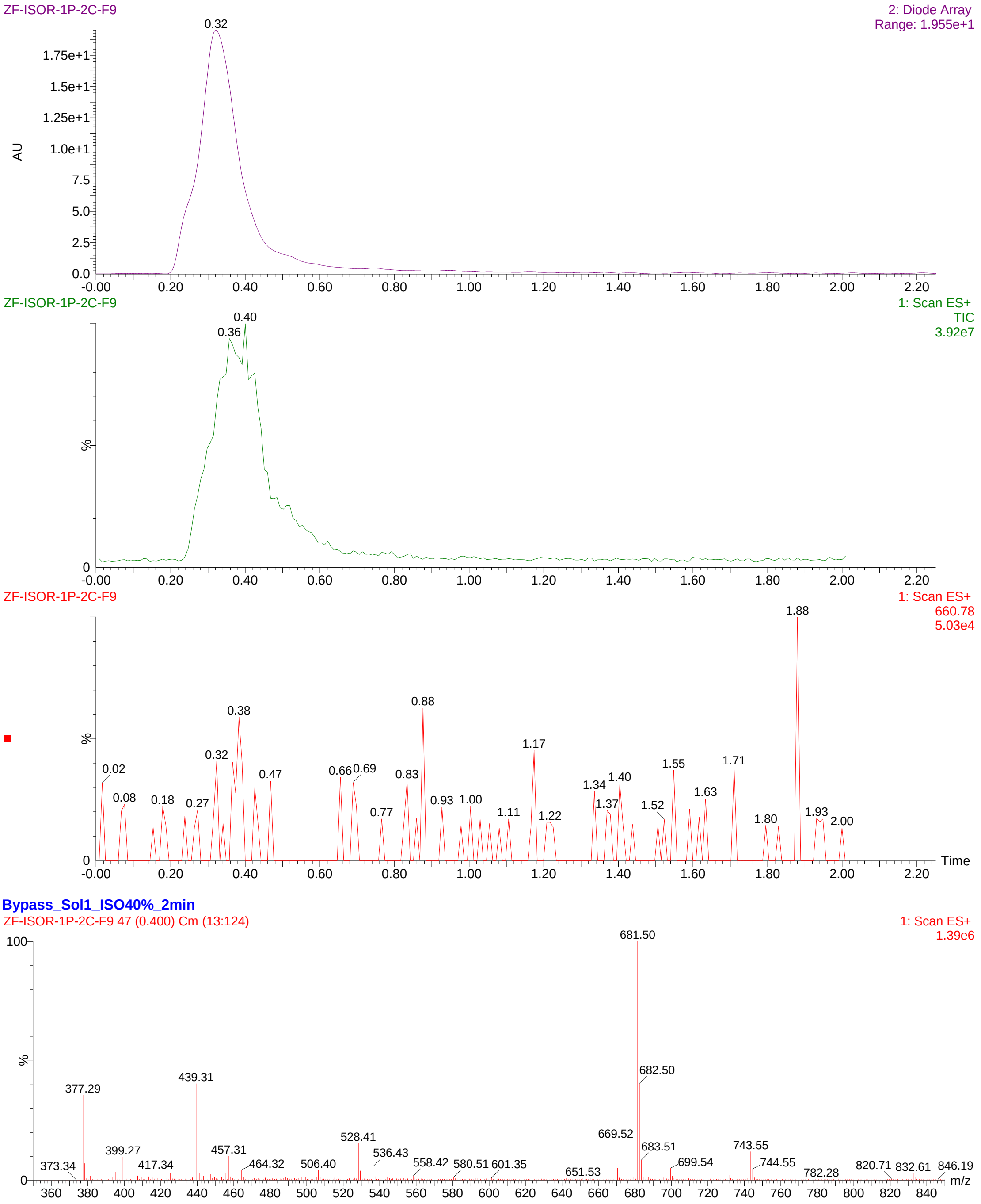

F11 (Yellow)

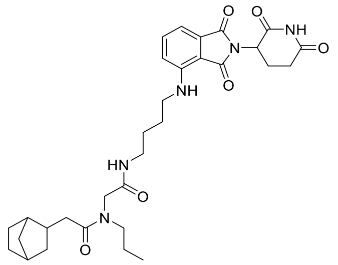

Exact Mass: 579,3057
Molecular Weight: 579,6980

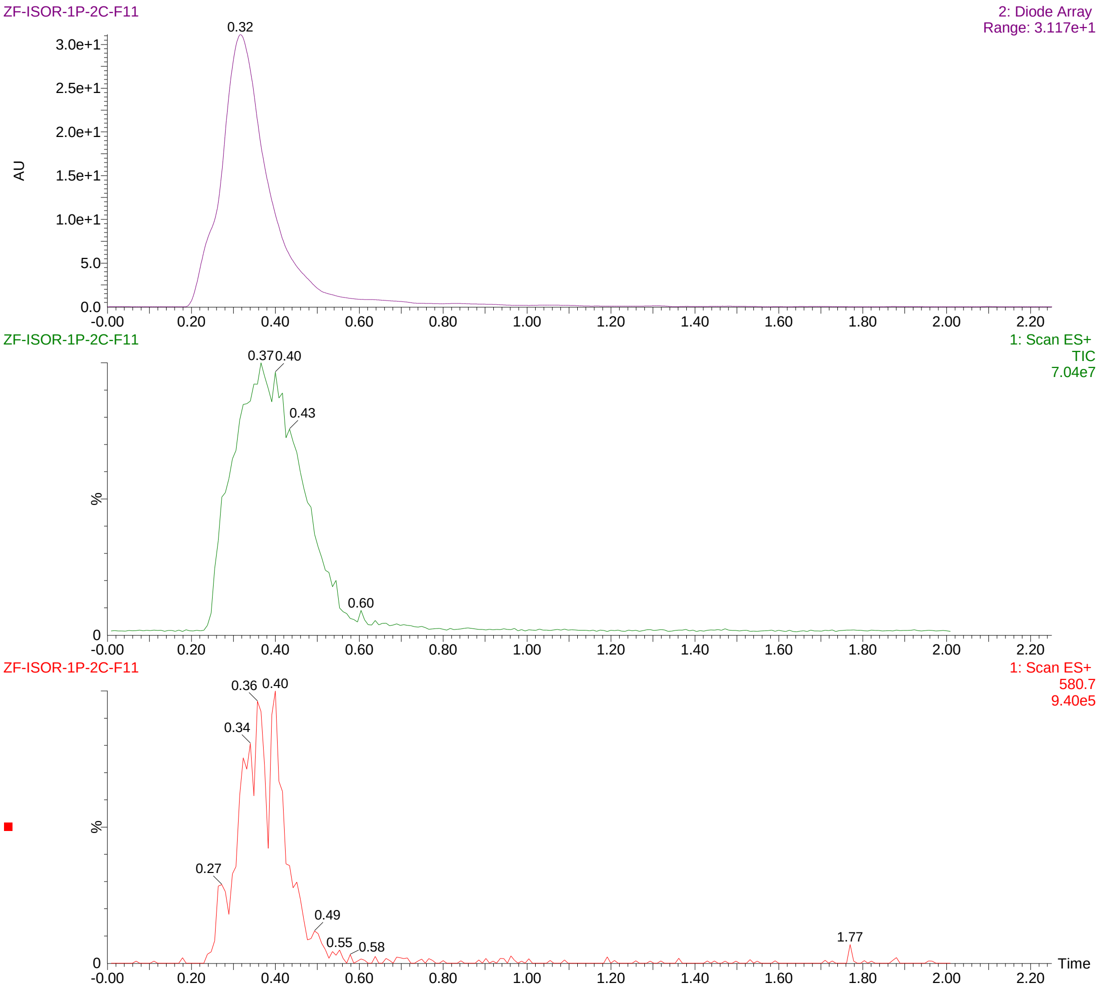

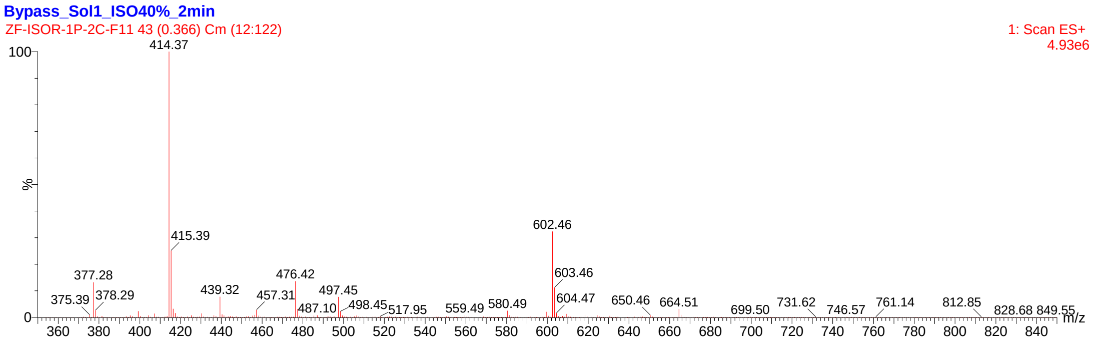

K11 (Green)

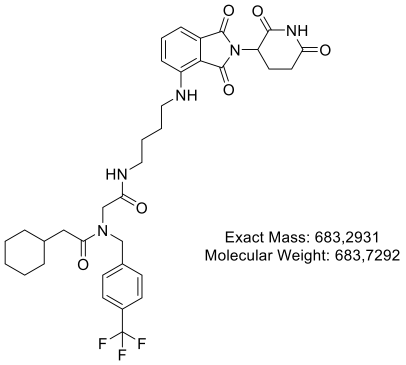

Exact Mass: 683,2931
Molecular Weight: 683,7292

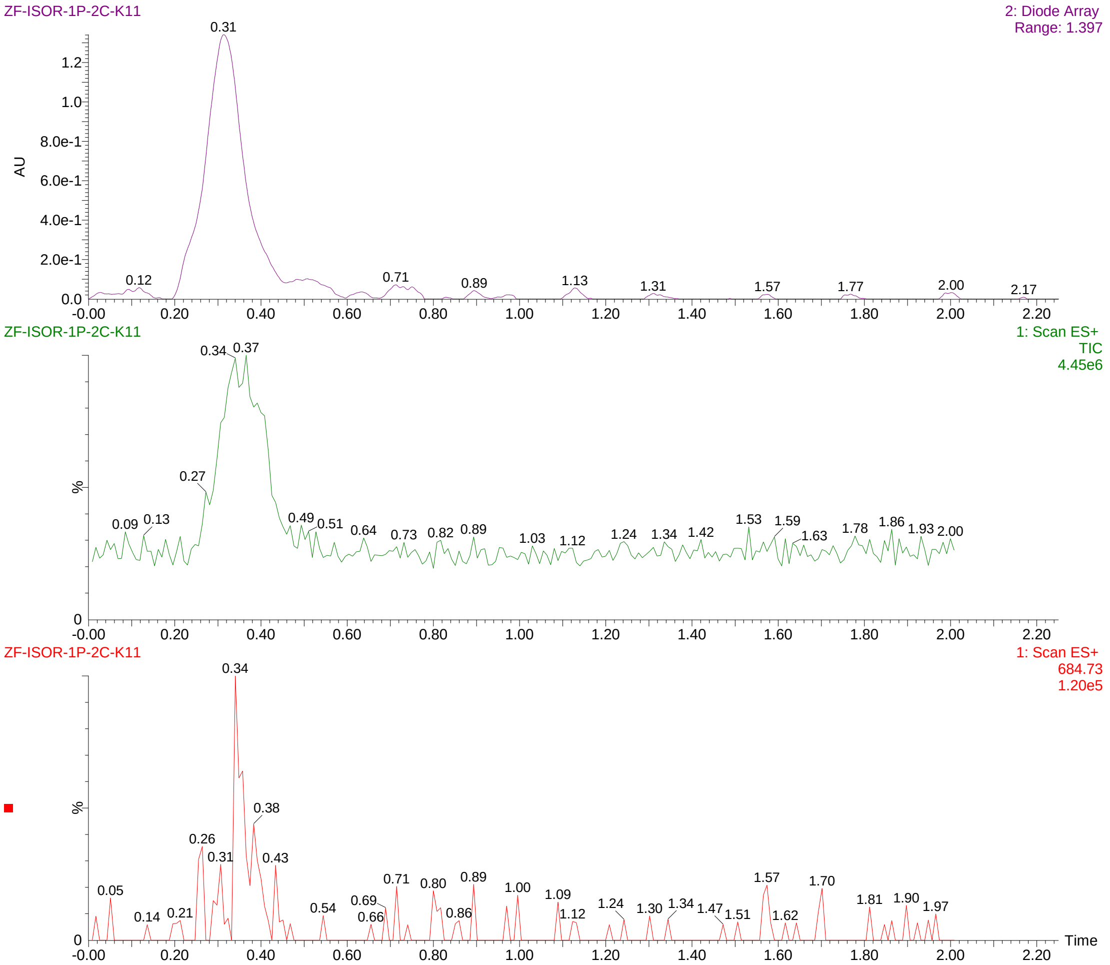

ZF-ISOR-1P-2C-K11
2: Diode Array
Range: 1.397
AU
ZF-ISOR-1P-2C-K11
1: Scan ES+
TIC
4.45e6
ZF-ISOR-1P-2C-K11
1: Scan ES+
684.73
1.20e5
Time

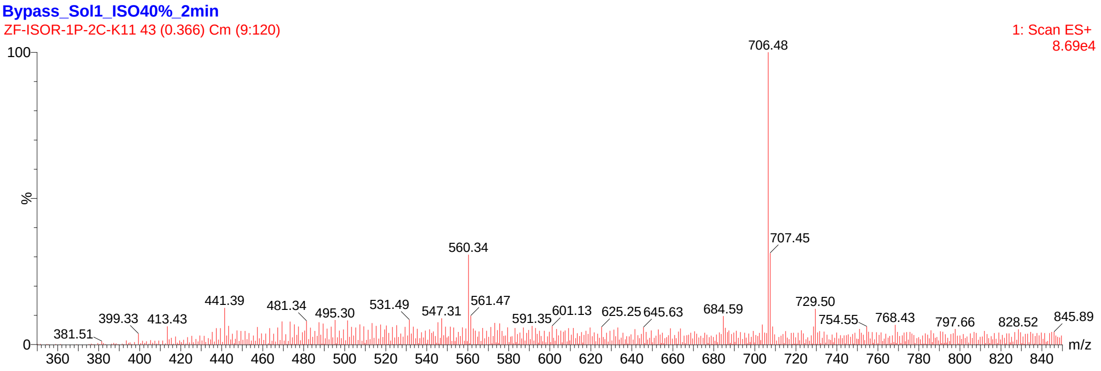

Bypass_Sol1_ISO40%_2min
ZF-ISOR-1P-2C-K11 43 (0.366) Cm (9:120)
1: Scan ES+
8.69e4
706.48
707.45
560.34
m/z

H12 (Green)

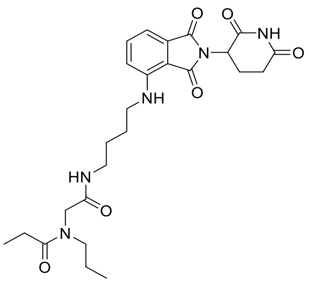

Exact Mass: 499,2431
Molecular Weight: 499,5680

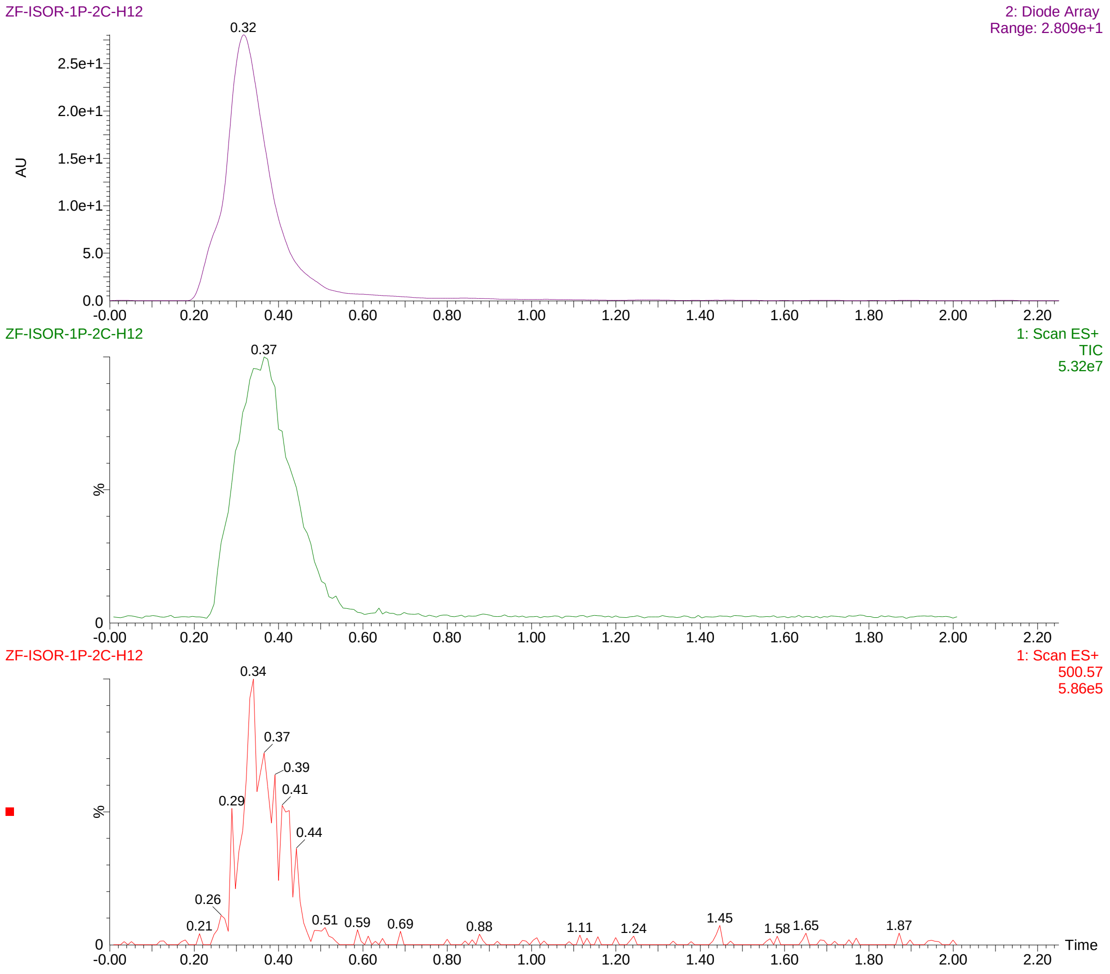

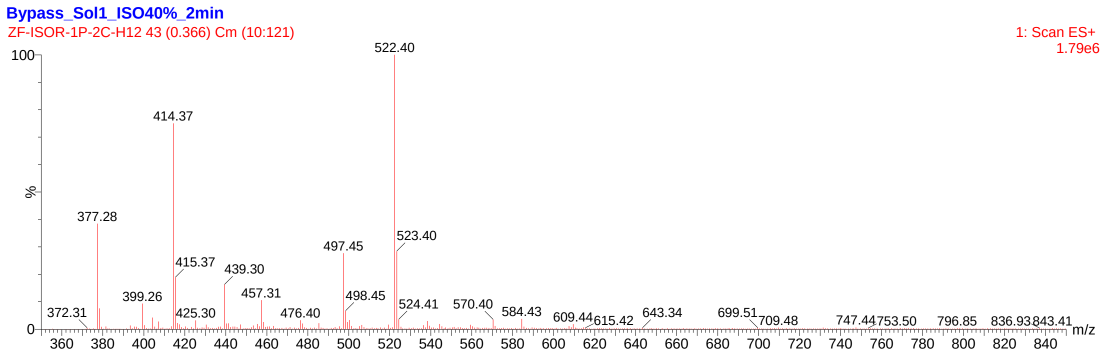

G13 (Green)

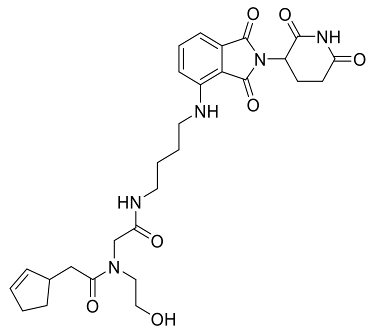

Exact Mass: 553,2536
Molecular Weight: 553,6160

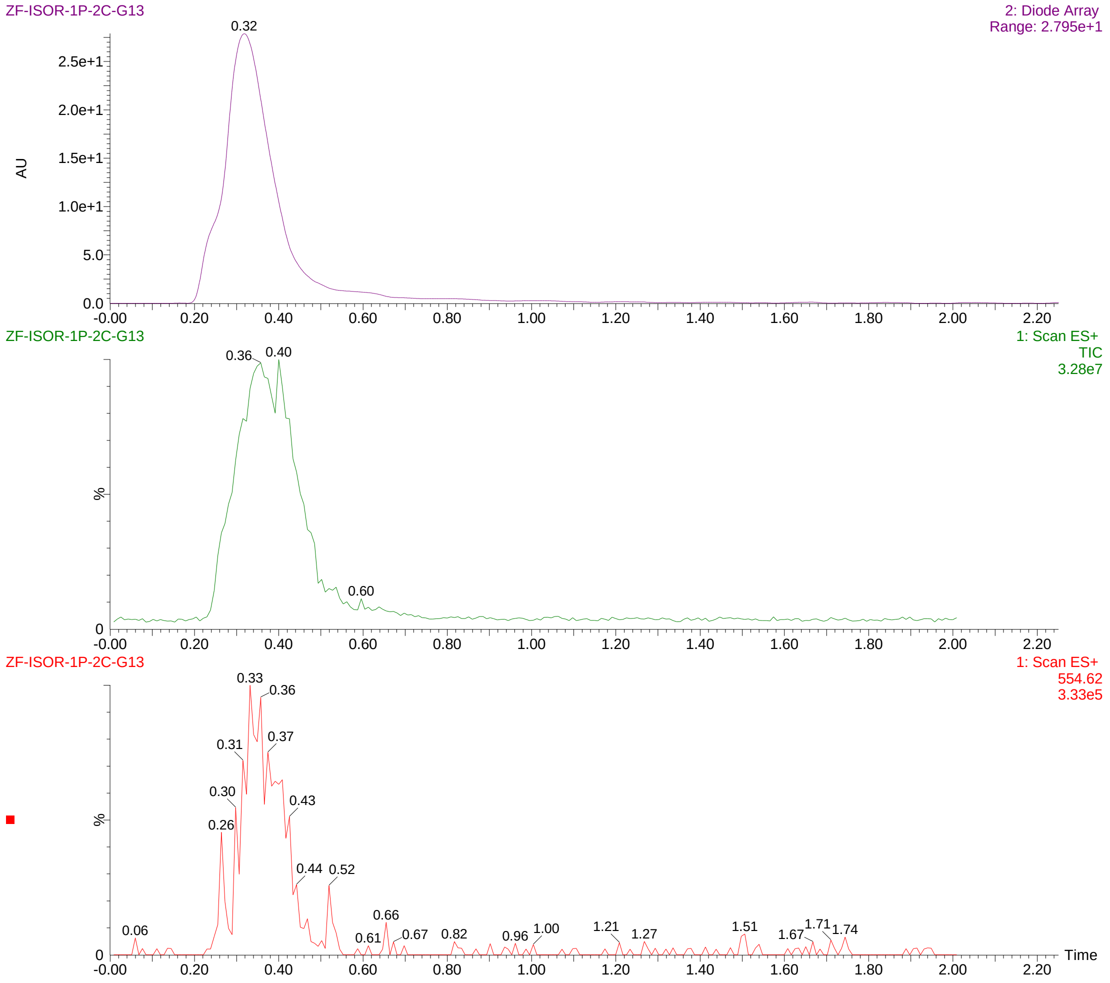

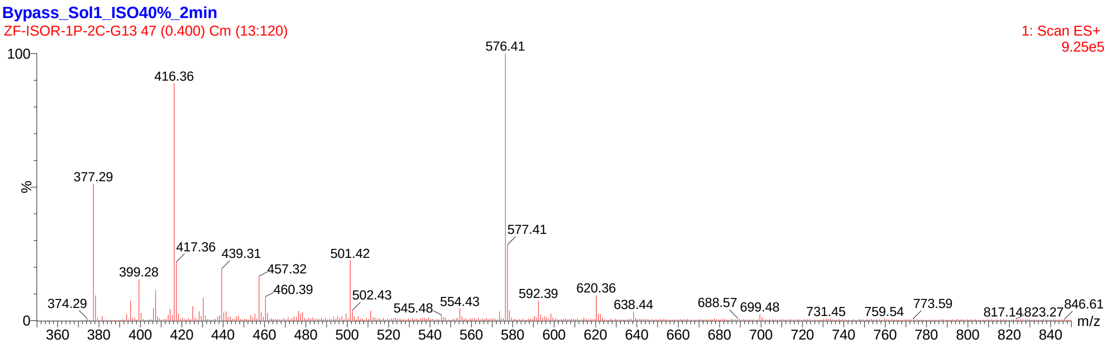

L13 (Green)

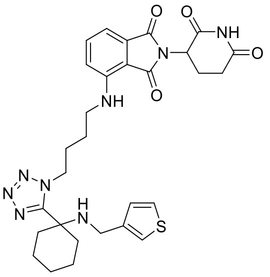

Exact Mass: 590,2424
Molecular Weight: 590,7030

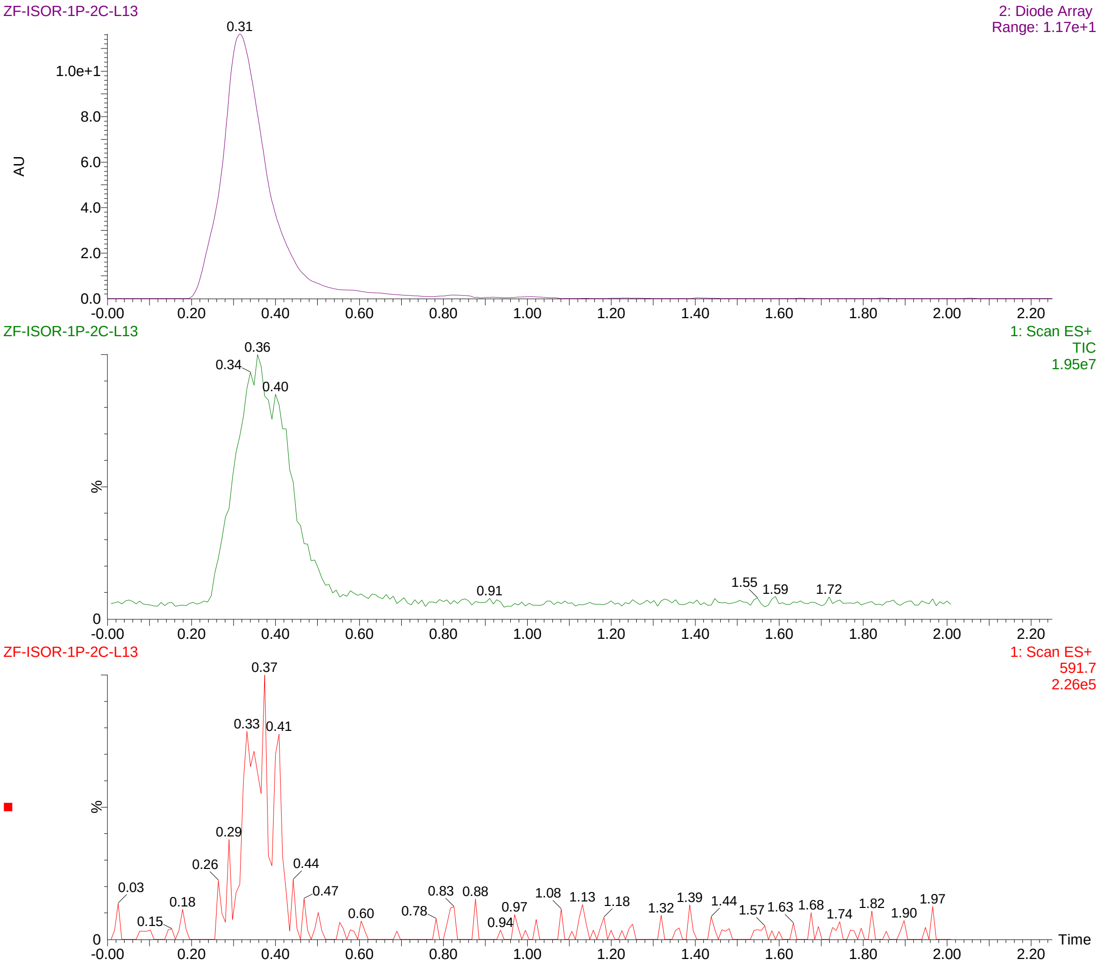

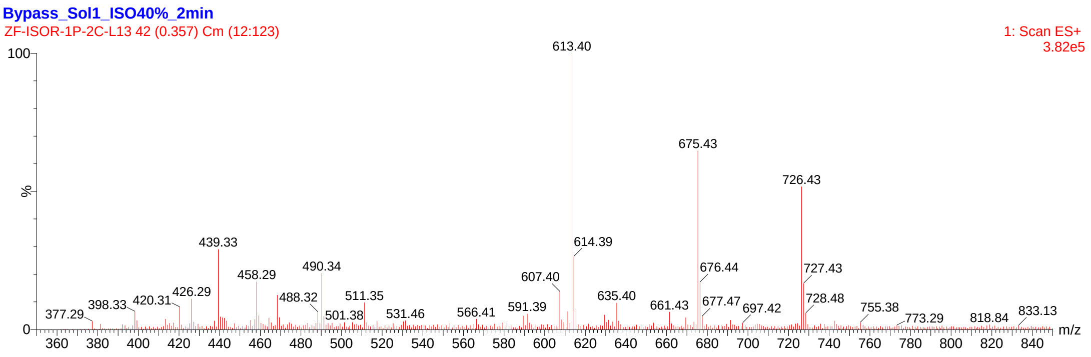

E14 (Green)

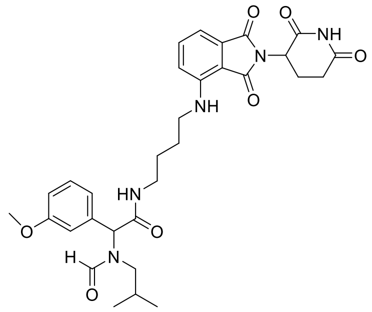

Exact Mass: 591,2693
Molecular Weight: 591,6650

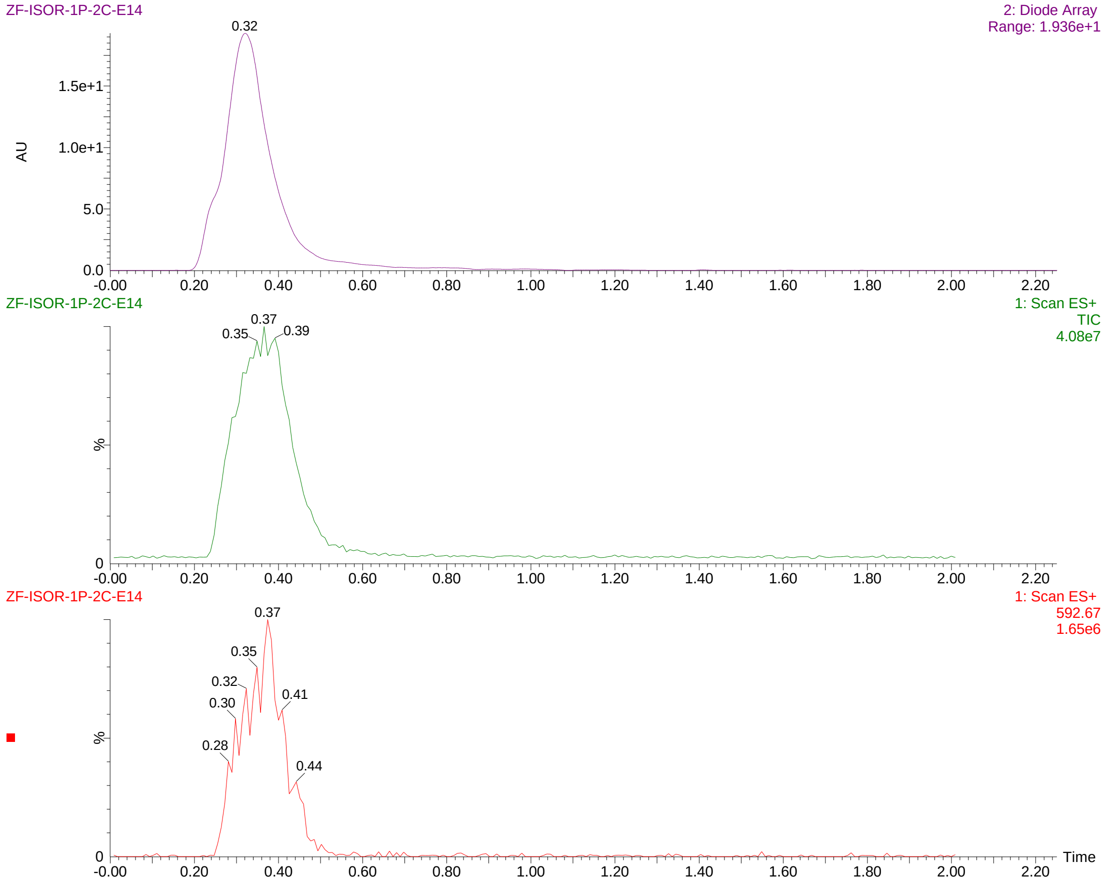

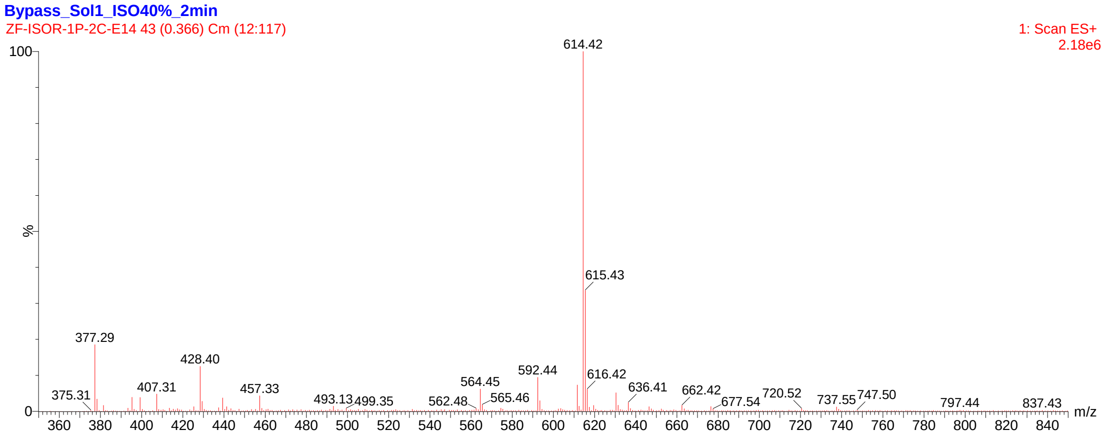

L18 (Green)

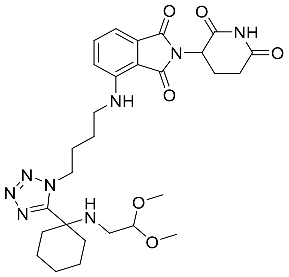

Exact Mass: 582,2914
Molecular Weight: 582,6620

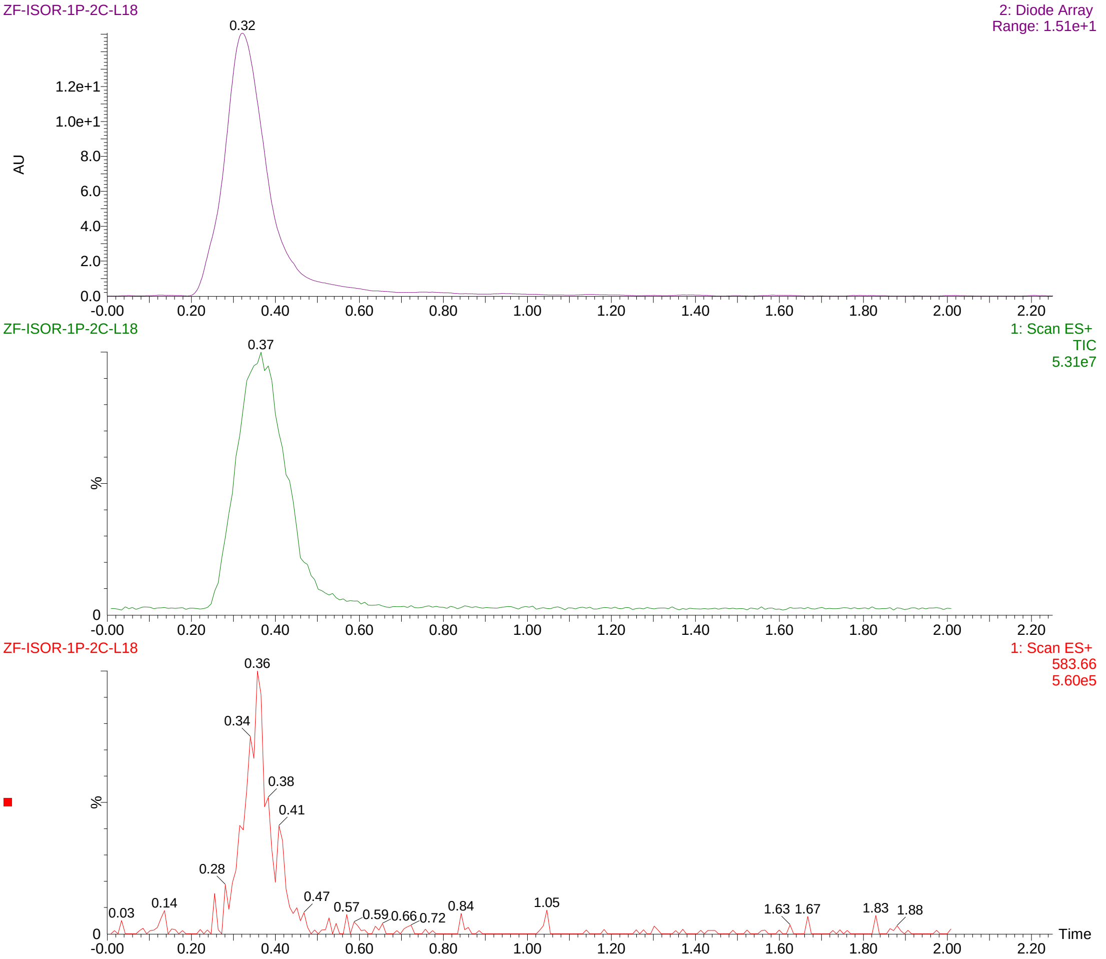

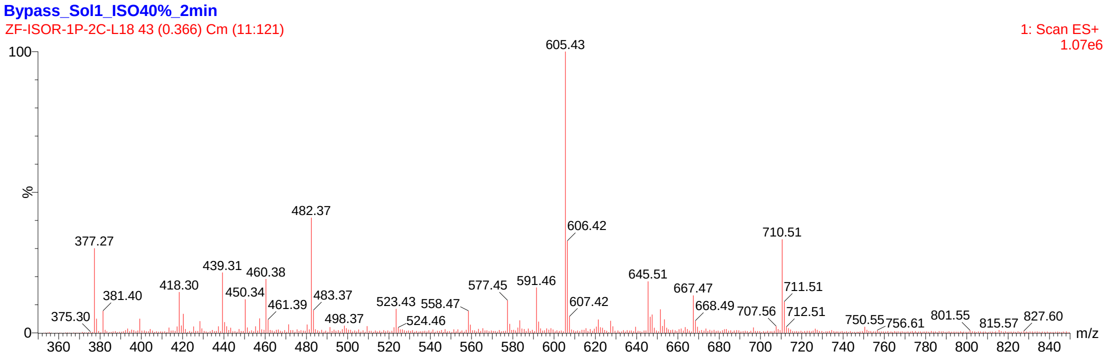

I19 (Yellow)

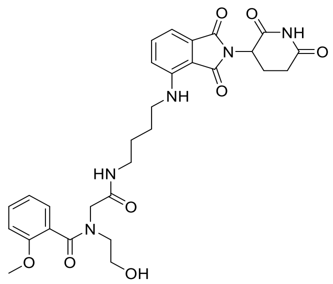

Exact Mass: 579,2329
Molecular Weight: 579,6100

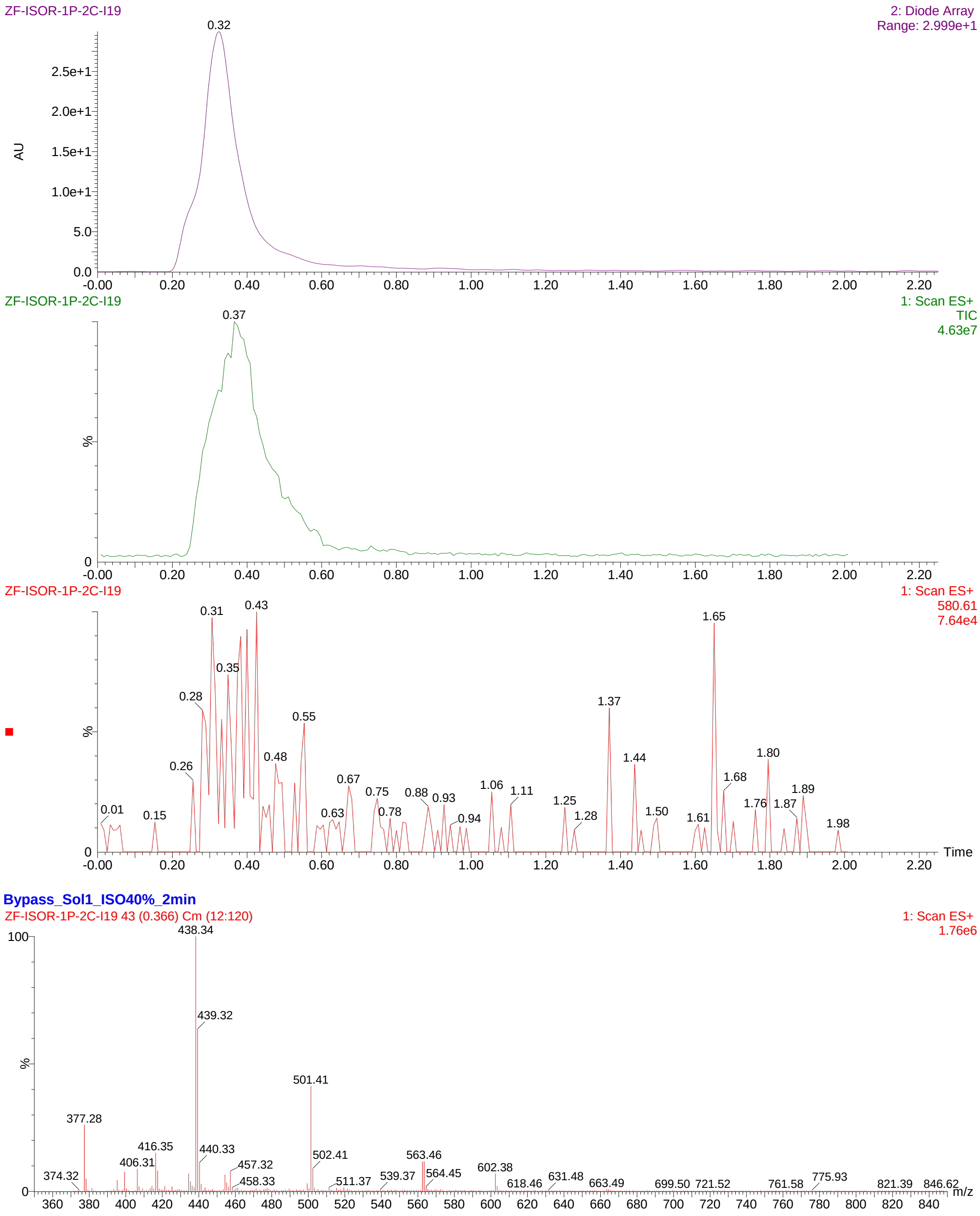

H23 (Yellow)

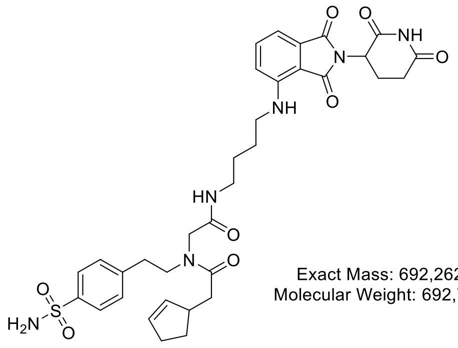

Exact Mass: 692,2628
Molecular Weight: 692,7880

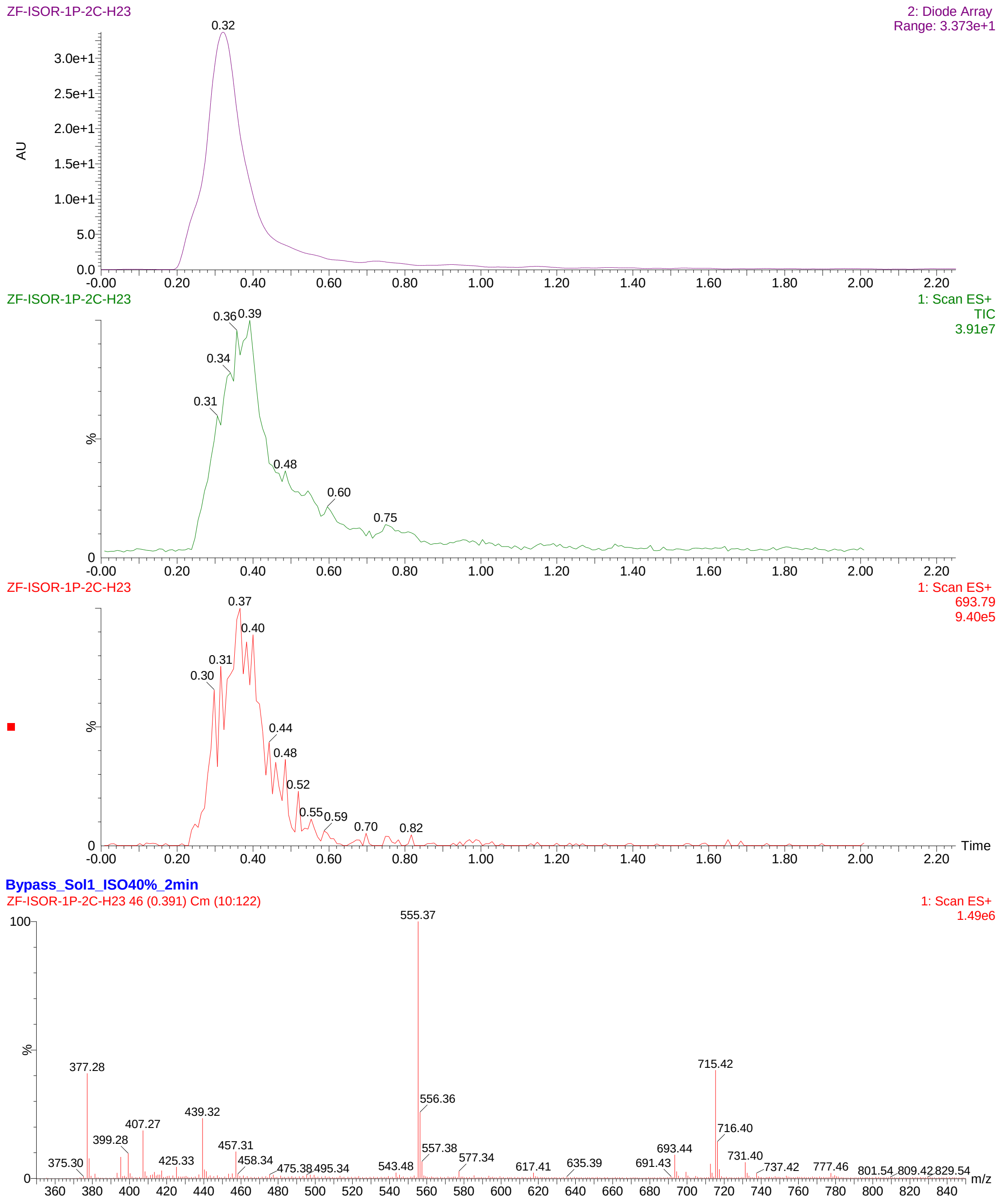

ZF-ISOR-1P-2C-H23
2: Diode Array
Range: 3.373e+1
0.32
AU
ZF-ISOR-1P-2C-H23
1: Scan ES+
TIC
3.91e7
0.36
0.39
0.34
0.31
0.48
0.60
0.75
ZF-ISOR-1P-2C-H23
1: Scan ES+
693.79
9.40e5
0.37
0.40
0.31
0.30
0.44
0.48
0.52
0.55
0.59
0.70
0.82
Time
Bypass_Sol1_ISO40%_2min
ZF-ISOR-1P-2C-H23 46 (0.391) Cm (10:122)
1: Scan ES+
1.49e6
555.37
377.28
715.42
556.36
439.32
407.27
716.40
399.28
457.31
557.38
693.44
731.40
375.30
425.33
458.34
577.34
691.43
475.38
495.34
543.48
617.41
635.39
737.42
777.46
801.54
809.42
829.54
m/z

P24 (Green)

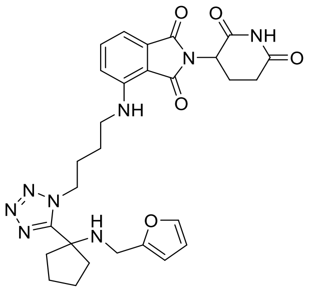

Exact Mass: 560,2496
Molecular Weight: 560,6150

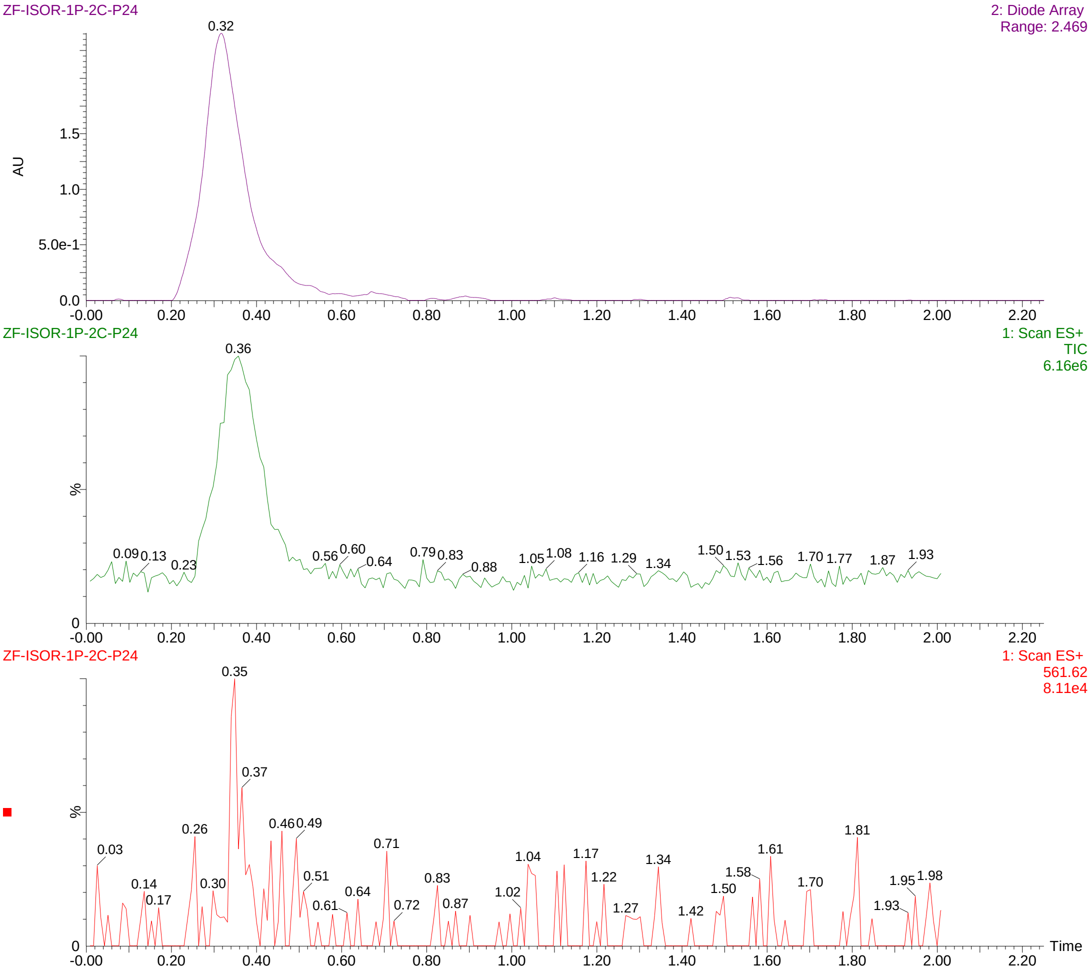

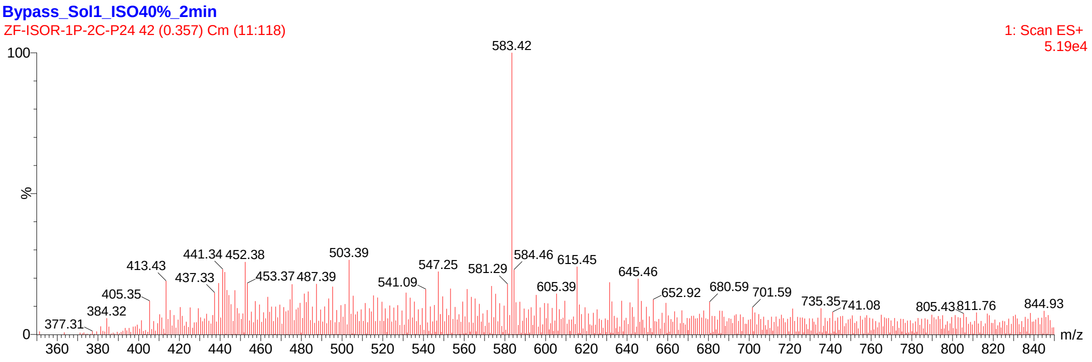

4. Heat plots

(A)

	1	2	3	4	5	6	7	8	9	10	11	12	13	14	15	16	17	18	19	20	21	22	23	24
A	0	0	1	0	0.2	0	0	0	0.2	1	0.1	0	0	0.1	0	0.3	0	0	1	0.7	0.1	0	0.5	0.3
B	0.8	0	0.1	0	0	0	0.1	0	0	0.3	0.2	0.1	0	0.3	0	0	0	0.1	0	0.6	0.1	0	0.4	0.3
C	0	0	1	0	0.2	1	1	0	0.8	0	0.7	1	0	0	0.1	0.2	0	0.1	0	0.3	0.4	0	0.6	0.4
D	0	0.6	0	0	0.3	1	0	1	1	0.6	0	0.1	0	0.3	0	0.1	0	0	0	0	1	0.2	0	1
E	0.2	0	0.1	0	0.2	0.3	0	0	0.7	0.1	0	0	0	1	0.2	0	0.2	0	0.3	0.4	0	0.1	0	0
F	0.1	0.1	0.1	0	0	0	0	0																

(B)

	1	2	3	4	5	6	7	8	9	10	11	12	13	14	15	16	17	18	19	20	21	22	23	24
F									1	0.1	0.3	0	0	0	0	0	1	1	0.2	0	0	1	0.5	0.2
G	0.3	0	0	1	0	0	0.4	0.3	0.1	0	0	0.3	1	0.2	0.5	1	0	0.3	0	0.2	0	0.6	0	0.4
H	0	0	0	0.3	0.5	0.1	1	1	0	0	0.6	1	0.6	0.1	1	0.1	0.1	0.5	0.2	0.2	1	1	0.4	0
I	1	0	0.5	0.4	0	0.1	0.4	0.3	1	0	1	1	0	1	0	1	0.1	0	0.1	0	0	1	1	1
J	0.2	0.5	0	0.8	0	1	1	1	0	1	0.1	0.3	1	0.1	1	0.2	0.6	0	0.3	1	1	0.1	1	0
K	0.7	0	0.1	1	0	0.7	1	0	1	0	1	0.5	0	0.4	0	1								

(C)

	1	2	3	4	5	6	7	8	9	10	11	12	13	14	15	16	17	18	19	20	21	22	23	24
K																	0.6	0	0.5	0	0	0.1	0.3	0.3
L	0.1	0	0	0	0.2	0	0	0.2	0	0.1	0	0.1	1	0.1	0.1	0.1	0	1	0	0	0.1	0	0	0.1
M	0.1	0.3	0.3	0.6	0	0.2	0	0.3	0.1	0.2	0.4	0.5	0.2	0	0.5	0.5	0	0	0.1	0.1	0.1	0	0.3	0
N	0	0.1	0	0	0	0.2	0	0	0.4	0.5	0.2	0.5	0.3	0.3	0.3	0.4	0.2	0.5	0	0	0.4	0.2	0.4	0.5
O	0.3	0	0.4	0.3	0	0.5	0	0.6	0.3	0.2	0.4	0.5	0.3	0.4	0.3	0.1	0.9	0	0.2	0	0	0	0	0
P	0	0	0.2	0	0.1	0.2	0	0	0.2	0.2	0.4	0	0.1	0	0.4	0	0	0.3	0.2	0	0	0.4	0.2	1

(D)

	1	2	3	4	5	6	7	8	9	10	11	12	13	14	15	16	17	18	19	20	21	22	23	24
A	0	0	1	0	0,2	0	0	0	0,2	1	0,1	0	0	0,1	0	0,3	0	0	1	0,7	0,1	0	0,5	0,3
B	0,8	0	0,1	0	0	0	0,1	0	0	0,3	0,2	0,1	0	0,3	0	0	0	0,1	0	0,6	0,1	0	0,4	0,3
C	0	0	1	0	0,2	1	1	0	0,8	0	0,7	1	0	0	0,1	0,2	0	0,1	0	0,3	0,4	0	0,6	0,4
D	0	0,6	0	0	0,3	1	0	1	1	0,6	0	0,1	0	0,3	0	0,1	0	0	0	0	1	0,2	0	1
E	0,2	0	0,1	0	0,2	0,3	0	0	0,7	0,1	0	0	0	1	0,2	0	0,2	0	0,3	0,4	0	0,1	0	0
F	0,1	0,1	0,1	0	0	0	0	0	1	0,1	0,3	0	0	0	0	0	1	1	0,2	0	0	1	0,5	0,2
G	0,3	0	0	1	0	0	0,4	0,3	0,1	0	0	0,3	1	0,2	0,5	1	0	0,3	0	0,2	0	0,6	0	0,4
H	0	0	0	0,3	0,5	0,1	1	1	0	0	0,6	1	0,6	0,1	1	0,1	0,1	0,5	0,2	0,2	1	1	0,4	0
I	1	0	0,5	0,4	0	0,1	0,4	0,3	1	0	1	1	0	1	0	1	0,1	0	0,1	0	0	1	1	1
J	0,2	0,5	0	0,8	0	1	1	1	0	1	0,1	0,3	1	0,1	1	0,2	0,6	0	0,3	1	1	0,1	1	0
K	0,7	0	0,1	1	0	0,7	1	0	1	0	1	0,5	0	0,4	0	1	0,6	0	0,5	0	0	0,1	0,3	0,3
L	0,1	0	0	0	0,2	0	0	0,2	0	0,1	0	0,1	1	0,1	0,1	0,1	0	1	0	0	0,1	0	0	0,1
M	0,1	0,3	0,3	0,6	0	0,2	0	0,3	0,1	0,2	0,4	0,5	0,2	0	0,5	0,5	0	0	0,1	0,1	0,1	0	0,3	0
N	0	0,1	0	0	0	0,2	0	0	0,4	0,5	0,2	0,5	0,3	0,3	0,3	0,4	0,2	0,5	0	0	0,4	0,2	0,4	0,5
O	0,3	0	0,4	0,3	0	0,5	0	0,6	0,3	0,2	0,4	0,5	0,3	0,4	0,3	0,1	0,9	0	0,2	0	0	0	0	0
P	0	0	0,2	0	0,1	0,2	0	0	0,2	0,2	0,4	0	0,1	0	0,4	0	0	0,3	0,2	0	0	0,4	0,2	1

Fig 1: Heat plots from the in-house program and mass analysis. **Green**: major product formation; **Yellow**: medium product formation; **Blue**: no product formation; □ resynthesized compound. (**A**) heat plot of the Ugi-reaction; (**B**) heat plot of the Ugi-formaldehyde reaction; (**C**) heat plot of the Ugi-tetrazole reaction; (**D**) heat plot of the whole plate.

5. Statistical reaction analysis

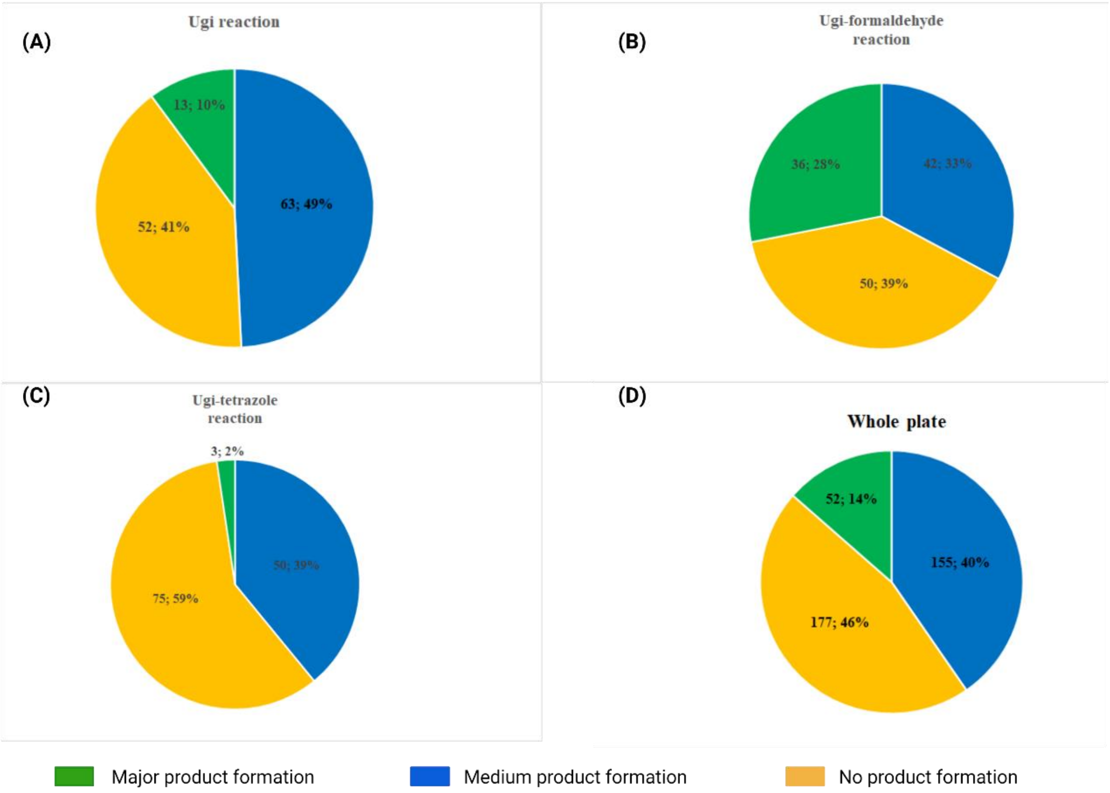

Fig 2: Pie chart of statistical reaction analysis**. (A)** pie chart of the Ugi-reaction. **(B)** pie chart of the Ugi-formaldehyde reaction. **(C)** pie chart of the Ugi-tetrazole reaction. **(D)** pie chart of the whole plate.

Ugi reaction:

Aldehyde/Ketone performance:

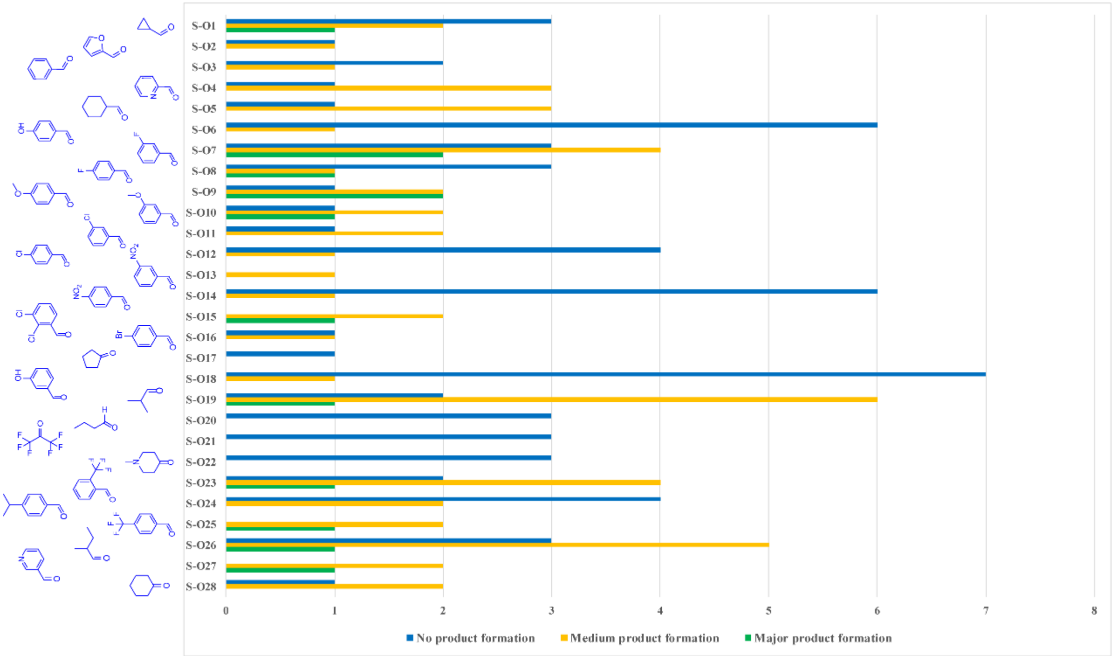

Fig 3: Performance of the aldehyde/ketone components in the Ugi reaction.

Amine performance:

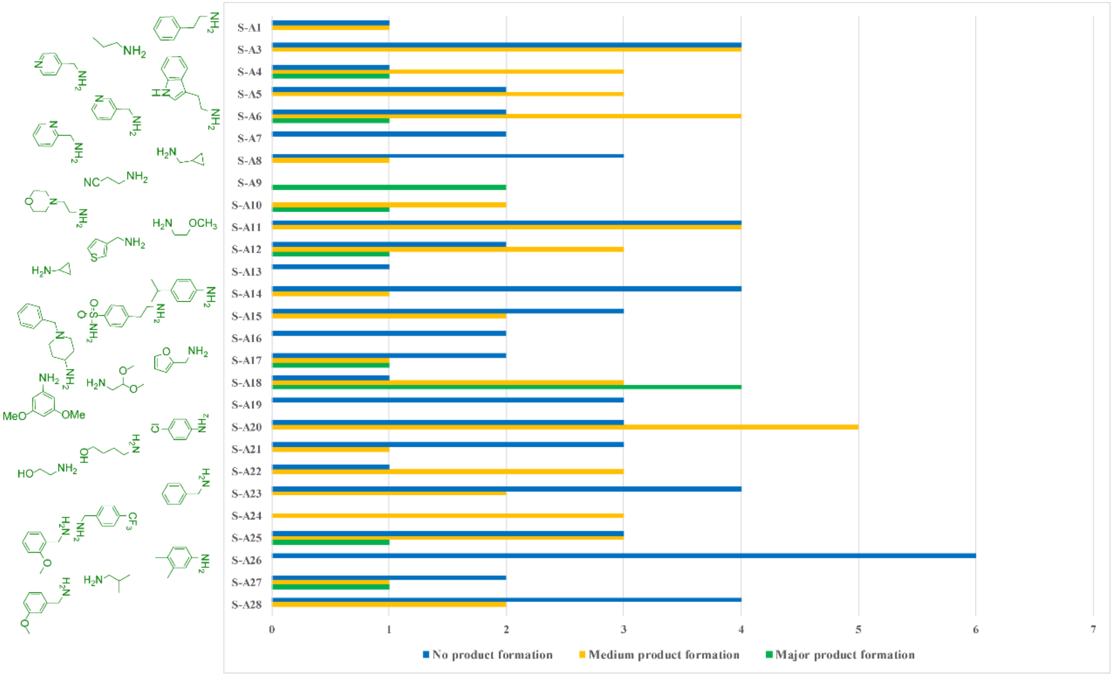

Fig 4: Performance of the amine components in the Ugi reaction.

Acid performance:

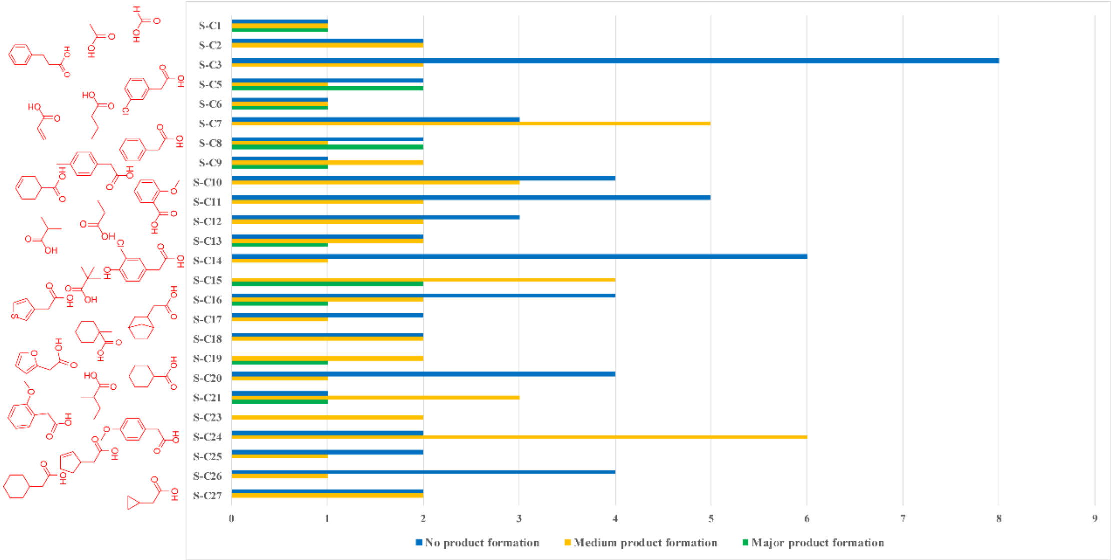

Fig 5: Performance of the acid components in the Ugi reaction.

Ugi-formaldehyde reaction

Amine performance:

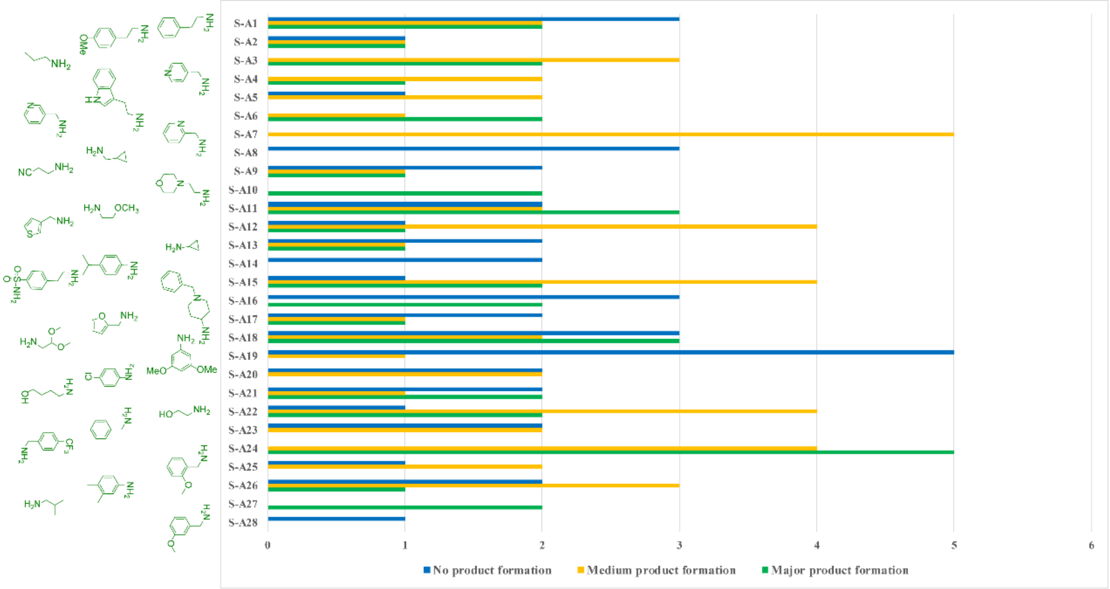

Fig 6: Performance of the amine components in the Ugi-formaldehyde reaction.

Acid performance:

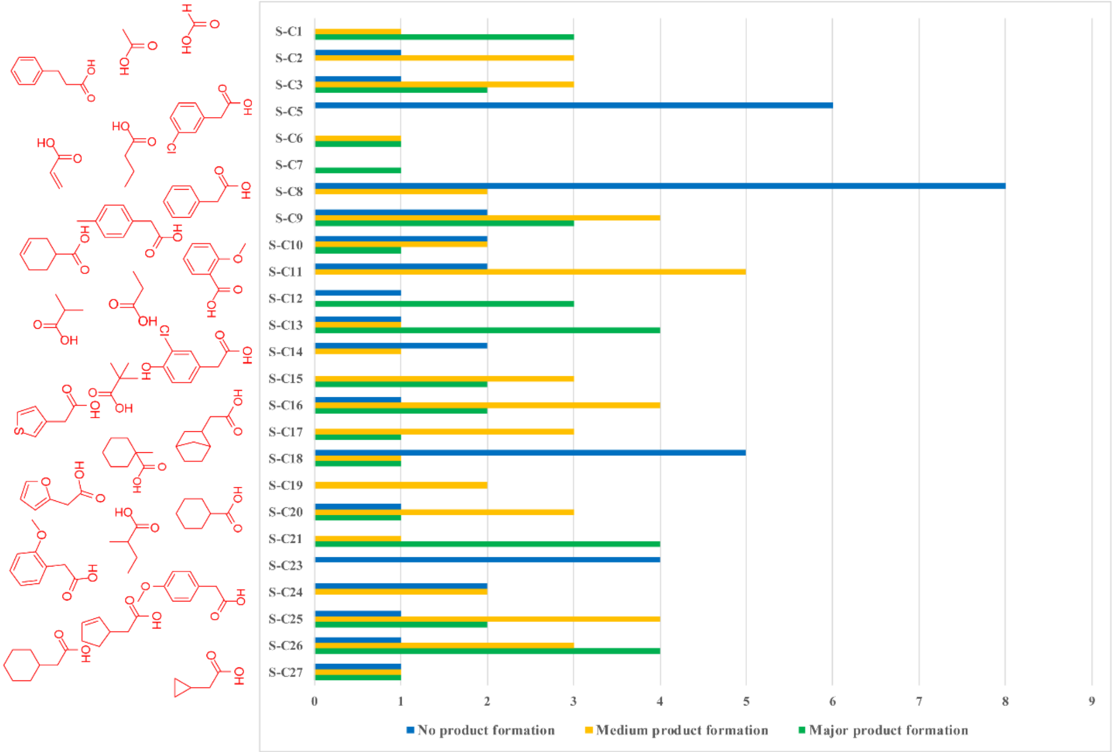

Fig 7: Performance of the acid components in the Ugi-formaldehyde reaction.

Ugi-tetrazole reaction

Aldehyde/Ketone performance:

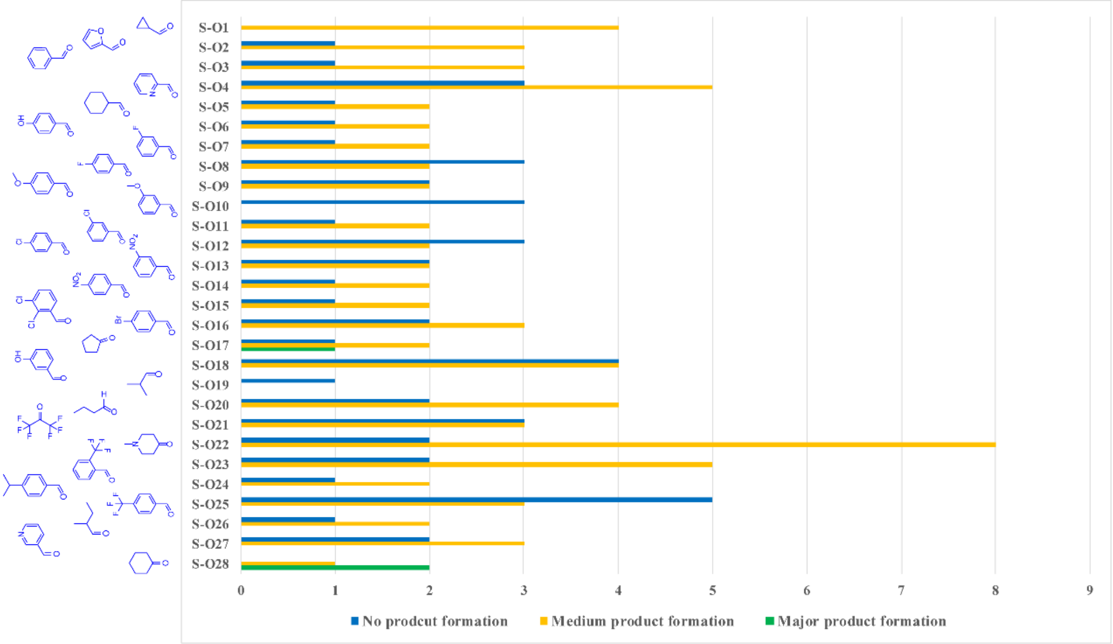

Fig 8: Performance of the aldehyde/ketone components in the Ugi-tetrazole reaction.

Amine performance:

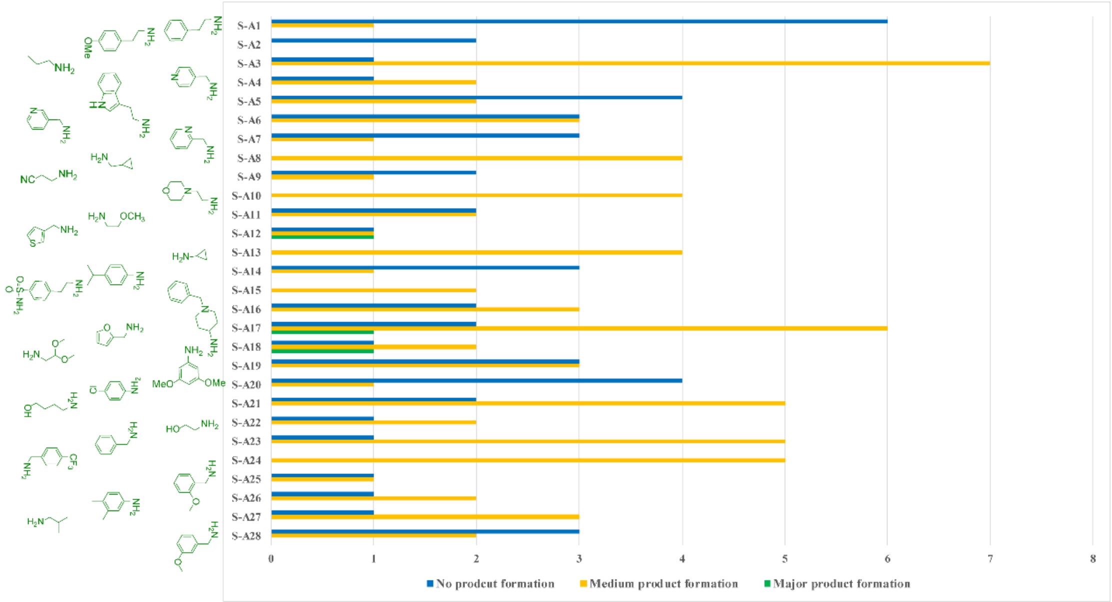

Fig 9: Performance of the amine components in the Ugi-tetrazole reaction.

6. Phenotypic screening of the destination plate

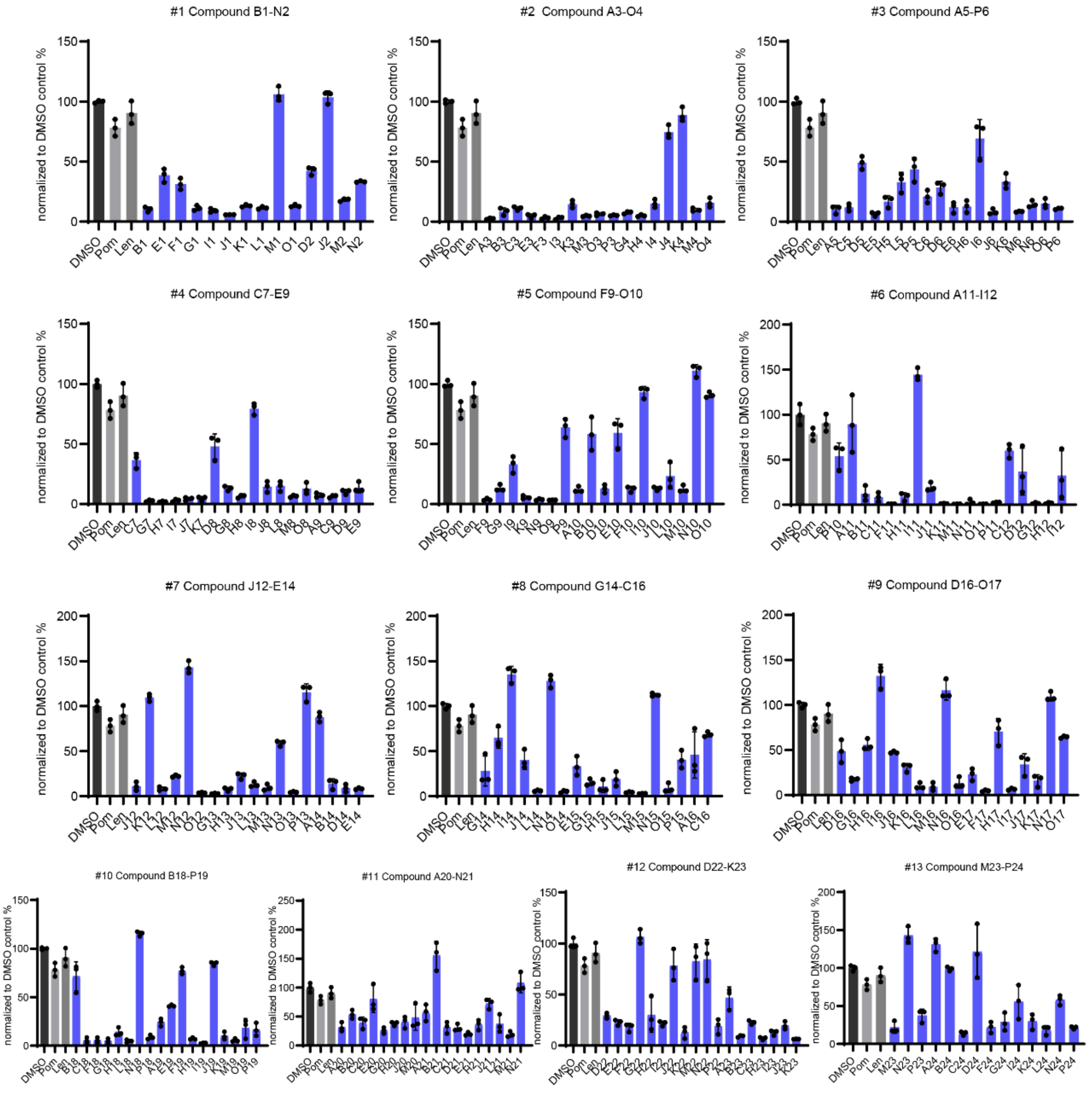

Fig 10: On-plate phenotypic screening result based on the MM.1S cell line viability assay at 1.0 µM for 96 hours. Graph bars represent mean values ± SD. n = 3 independent biological replicates.

7. CRBN KO RPMI/8226 cell viability assay

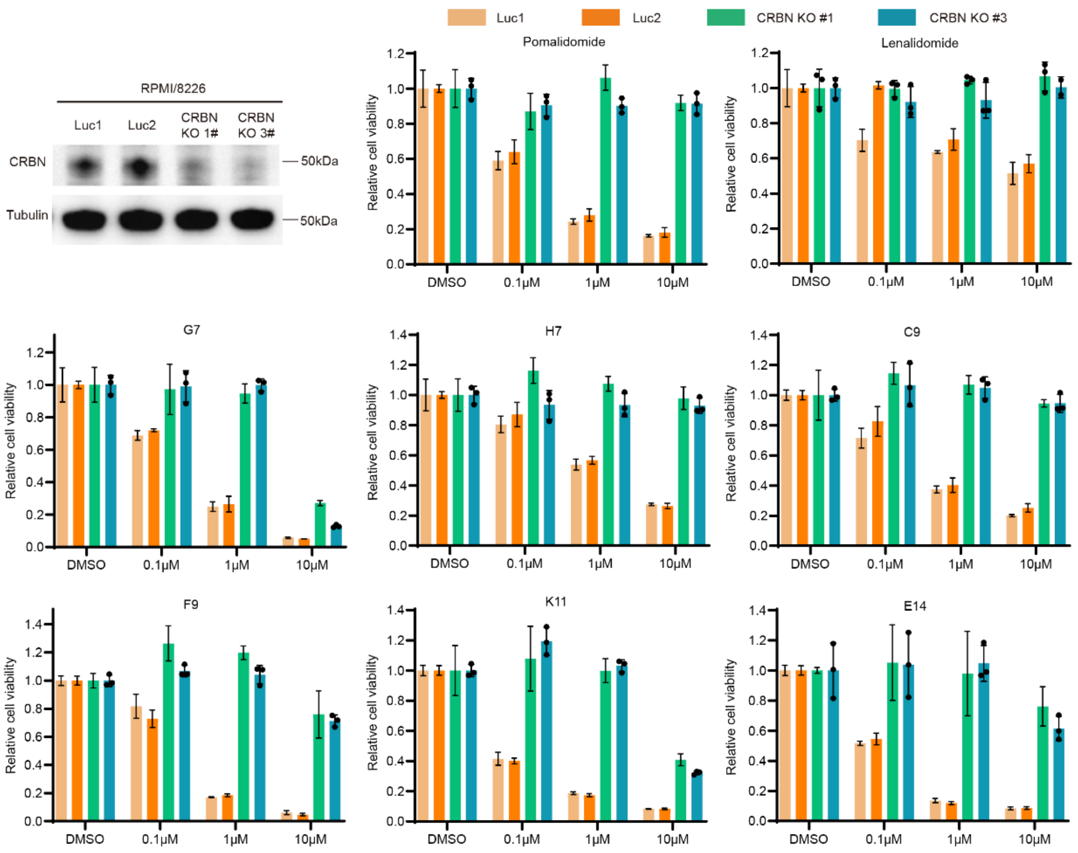

Fig 11: Luc1 and Luc2 cell lines are RPMI/8226 cell line without CRBN knockout. Cell viability assay in the PMI/8226 wild-type and CRBN knockout cell lines. Graph bars represent mean values ± SD. n = 3 independent biological replicates.

8. General experimental data for the mmol scale synthesis

8.1 General procedure for isocyanide synthesis

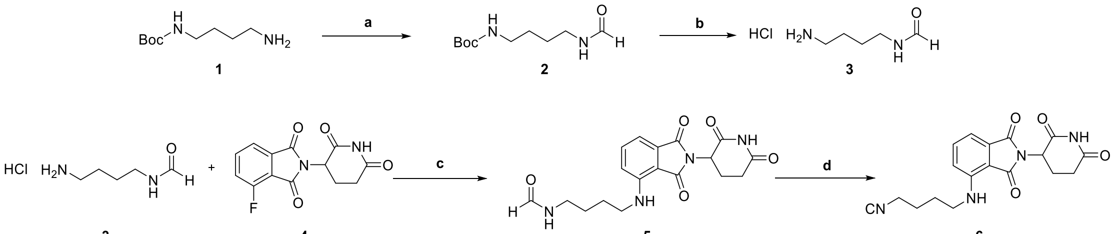

Scheme 1: a): Ethyl formate, reflux, overnight. b): 4N HCl in dioxane, DCM, rt, 4 hours. c): DIPEA (4.0 eq), anhydrous DMF, 90 °C, overnight. d): Phosphorus oxychloride (2.0 eq), Et_3N (5.0 eq), anhydrous THF, -78 °C addition, -78 °C to -20 °C reaction over 5 hours, 5 hours.

8.1.1 General synthesis procedure for tert-butyl (4-formamidobutyl)carbamate (2)

Procedure **a**: tert-butyl (4-aminobutyl)carbamate **1** (900 mg, 4.78 mmol) was dissolved in the ethyl formate (20 mL) and kept reflux for overnight. The solvent was removed under reduced pressure. The obtained crude formamide **2** was further used without further purification. White solid, quantative yield.

8.1.2 General synthesis procedure for N-(4-aminobutyl)formamide hydrogen chloride (3)

Procedure **b**: tert-butyl (4-formamidobutyl)carbamate **2** (870 mg, 4.0 mmol) was dissolved in the DCM (15 mL) and under stirring 4N HCl in dioxane (8 mL) was added to the reaction mixture. The reaction was kept for 4 hours at room temperature. Then the solvent was removed under reduced pressure to get the crude product. The product was washed by diethylether three times (3 x 15 mL) to get the white solid **3**. White solid, quantative yield.

8.1.3 General synthesis procedure for N-(4-((2-(2,6-dioxopiperidin-3-yl)-1,3-dioxoisoindolin-4-yl)amino)butyl)formamide (5)

Procedure **c**: N-(4-aminobutyl)formamide hydrogen chloride **3** (365mg, 2.4 mmol, 1.2 eq) was added to the stirred solution of 2-(2,6-dioxopiperidin-3-yl)-4-fluoroisoindoline-1,3-dione **4** (552 mg, 2 mmol, 1.0 eq) in anhydrous DMF (6.0 mL) and DIPEA (517 mg, 4.0 mmol, 4.0 eq). The reaction mixture was stirred at 90 ^{o}C for 12 hours. After the reaction mixture cooling down to room temperature, the brine (50 mL) was added to the reaction mixture and extracted three times with ethyl acetate (3 x 50 mL). The combined layer was dried over anhydrous Na_2SO_4, and purified by flash column chromatography to get the pure compound **5**: petroleum ether / ethyl acetate 30-100%. Yellow solid, yield 65%.

8.1.4 General synthesis procedure for 2-(2,6-dioxopiperidin-3-yl)-4-((4-isocyanobutyl)amino)isoindoline-1,3-dione (6)

Procedure **d**: Corresponding formamide **5** (372 mg, 1.0 mmol, 1.0 eq) was dissolved in the anhydrous THF (3 mL), then Et_3N (506 mg, 5.0 mmol, 5.0 eq) was added to the reaction mixture. Under -78 ^{o}C, phosphorus oxychloride (306 mg, 2.0 mmol, 2.0 eq) was dropwise added to the reaction mixture over 15 min. After the addition was finished, the reaction was stirred and warmed up to -20 ^{o}C over 5 hours. The reaction was monitored by TLC. When the reaction was finished, the reaction mixture was poured slowly in an ice-cold saturated solution of $NaHCO_3$ (30 mL) and after 30 min of stirring, the reaction mixture was extracted with DCM (3 x 50 mL). The combined organic phases were dried over anhydrous Na_2SO_4, filtered and the solvent was removed under reduced pressure. The residue was purified by flash column chromatography to get the pure compound **6**: petroleum ether / ethyl acetate 0-100%. Yellow solid, yield 65%.

8.2 General mmol synthesis procedure for Multiple Component Reaction

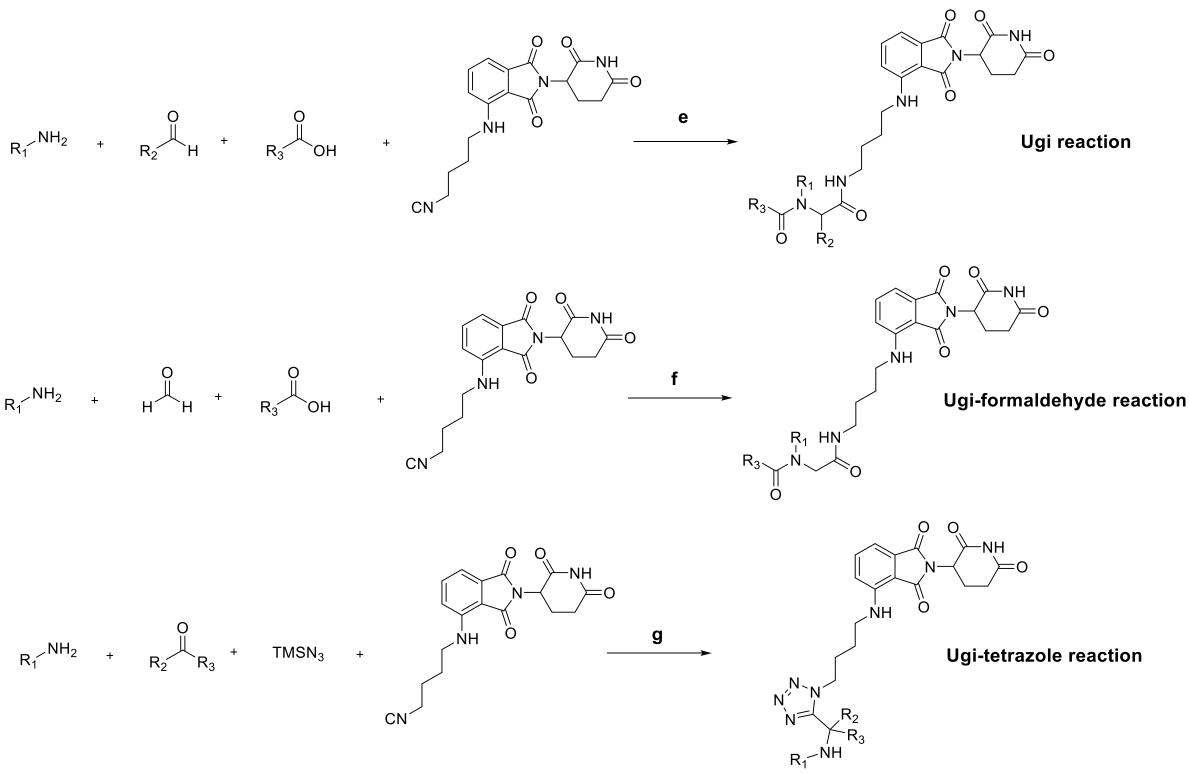

Scheme 2: synthesis route of the MCR.

8.2.1 General mmol synthesis procedure for Ugi reaction

Procedure **e**: Amine (1.0 mmol, 1.0 eq), aldehyde (1.0 mmol, 1.0 eq), acid (1.0 mmol, 1.0 eq) and isocyanide (354 mg, 1.0 mmol, 1.0 eq) were dissolved in the TFE (1 mL), and reaction mixture was stirred at room temperature for overnight. The reaction was monitored by TLC. When reaction was finished, the reaction mixture was purified by flash column chromatography to get the pure product: DCM/MeOH: 0-10%.

8.2.2 General mmol synthesis procedure for Ugi-formaldehyde reaction

Procedure **f**: Amine (1.0 mmol, 1.0 eq), paraformaldehyde (30 mg, 1.0 mmol, 1.0 eq), acid (1.0 mmol, 1.0 eq) and isocyanide (354 mg, 1.0 mmol, 1.0 eq) were dissolved in the TFE (1 mL), and reaction mixture was stirred at room temperature for overnight. The reaction was monitored by TLC. When reaction was finished, the reaction mixture was purified by flash column chromatography to get the pure product: DCM/MeOH: 0-10%.

8.2.3 General mmol synthesis procedure for Ugi-tetrazole reaction

Procedure **g**: Amine (1.0 mmol, 1.0 eq), aldehyde (1.0 mmol, 1.0 eq), $TMSN_3$ (115 mg, 1.0 mmol, 1.0 eq) and isocyanide (354 mg, 1.0 mmol, 1.0 eq) were dissolved in the TFE (1 mL), and

reaction mixture was stirred at room temperature for overnight. The reaction was monitored by TLC. When reaction was finished, the reaction mixture was purified by flash column chromatography to get the pure product: DCM/MeOH: 0-10%.

5: N-(4-((2-(2,6-dioxopiperidin-3-yl)-1,3-dioxoisoindolin-4-yl)amino)butyl)formamide:

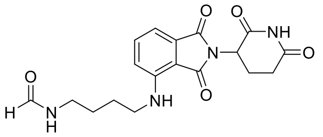

5

Yellow solid, procedure **c**, purified yield: 65%. ^{1}H NMR (500 MHz, Methanol-*d$_4$*) δ 8.02 (d, J = 6.5 Hz, 1H), 7.53 – 7.45 (m, 1H), 6.99 (d, J = 7.4 Hz, 2H), 5.08 – 5.00 (m, 1H), 3.28 – 3.15 (m, 3H), 3.04 (q, J = 6.1 Hz, 1H), 2.90 – 2.79 (m, 1H), 2.76 – 2.63 (m, 2H), 2.13 – 2.04 (m, 1H), 1.70 – 1.57 (m, 4H); ^{13}C NMR (126 MHz, Methanol-*d$_4$*) δ 173.34, 170.30, 169.36, 167.92, 162.46, 146.73, 135.88, 132.48, 116.62, 110.44, 109.62, 48.80, 41.60, 37.20, 30.87, 26.40, 26.26, 22.42.

6: 2-(2,6-dioxopiperidin-3-yl)-4-((4-isocyanobutyl)amino)isoindoline-1,3-dione:

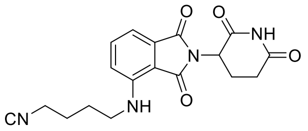

6

Yellow solid, procedure **d**, purified yield: 65%. ^{1}H NMR (500 MHz, DMSO-*d$_6$*) δ 11. 09 (s, 1H), 7.62 – 7.55 (m, 1H), 7.12 (d, J = 8.6 Hz, 1H), 7.03 (d, J = 7.0 Hz, 1H), 6.64 (d, J = 6.1 Hz, 1H), 5.10 – 4.99 (m, 1H), 3.59 – 3.52 (m, 2H), 3.39 – 3.33 (m, 2H), 2.95 – 2.81 (m, 1H), 2.63 – 2.51 (m, 2H), 2.06 – 2.00 (m, 1H), 1.66 (d, J = 5.7 Hz, 4H); ^{13}C NMR (126 MHz, DMSO-*d$_6$*) δ 173.29, 170.57, 169.33, 167.77, 159.10, 156.05, 155.96, 146.77, 132.73, 117.69, 110.93, 109.64, 49.01, 41.37, 41.35, 41.33, 31.45, 29.46, 26.38, 26.04, 22.63.

G7: 2-(bicyclo[2.2.1]heptan-2-yl)-N-(4-chlorophenyl)-N-(2-((4-((2-(2,6-dioxopiperidin-3-yl)-1,3-dioxoisoindolin-4-yl)amino)butyl)amino)-2-oxoethyl)acetamide:

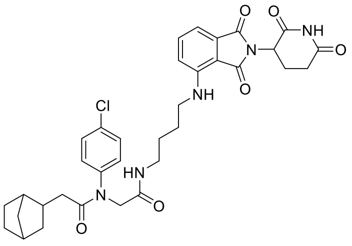

G7

Yellow solid, procedure **f**, purified yield: 22%. ^{1}H NMR (500 MHz, Chloroform-*d*) δ 8.20 (s, 1H), 7.49 (t, J = 8.5, 7.1 Hz, 1H), 7.40 – 7.35 (m, 2H), 7.23 – 7.17 (m, 2H), 7.09 (d, J = 7.1 Hz, 1H), 6.89 (d, J = 8.6 Hz, 1H), 6.46 (t, J = 5.8 Hz, 1H), 6.23 (t, J = 5.8 Hz, 1H), 4.97 – 4.85 (m, 1H), 4.24 – 4.13 (m, 2H), 3.37 – 3.24 (m, 4H), 2.93 – 2.68 (m, 3H), 2.18 – 2.03 (m, 3H), 1.97 – 1.84 (m, 3H), 1.71 – 1.60 (m, 7H), 1.48 – 1.38 (m, 3H), 1.21 – 1.14 (m, 1H), 1.12 – 1.05 (m, 1H); ^{13}C NMR (126 MHz, $CDCl_3$) δ 173.65, 171.02, 169.52, 168.93, 168.37, 167.60, 146.87, 141.48, 136.23, 134.21, 132.49, 130.09, 129.30, 116.71, 111.61, 110.01, 54.22, 48.90, 42.20, 40.94, 40.62, 39.01, 38.70, 37.77, 36.64, 35.20, 31.44, 29.78, 28.52, 27.06, 26.56, 22.82; HRMS calcd for $C_{34}H_{39}ClN_5O_6$: $[M+H]^+$ 648.2511, found $[M+H]^+$ 648.2582.

H7: N-(cyanomethyl)-N-(2-((4-((2-(2,6-dioxopiperidin-3-yl)-1,3-dioxoisoindolin-4-yl)amino)butyl)amino)-2-oxoethyl)-3-phenylpropanamide:

Yellow solid, procedure **f**, purified yield: 35%. ^{1}H NMR (500 MHz, DMSO-d_6) δ 11.12 (s, 1H), 8.00 (t, J = 104.8, 5.7 Hz, 1H), 7.61 – 7.54 (m, 1H), 7.28 – 7.22 (m, 3H), 7.20 – 7.07 (m, 3H), 7.03 (d, J = 7.0 Hz, 1H), 6.60 – 6.50 (m, 1H), 5.10 – 5.02 (m, 1H), 4.04 (s, 1H), 3.90 (s, 1H), 3.65 (t, J = 6.7 Hz, 1H), 3.52 (t, J = 6.9 Hz, 1H), 3.32 – 3.25 (m, 2H), 3.16 – 3.07 (m, 2H), 2.94 – 2.84 (m, 1H), 2.82 – 2.72 (m, 4H), 2.68 (t, J = 6.8 Hz, 1H), 2.63 – 2.53 (m, 2H), 2.48 (d, J = 3.1 Hz, 1H), 2.07 – 1.98 (m, 1H), 1.61 – 1.44 (m, 4H); ^{13}C NMR (126 MHz, DMSO) δ 173.32, 172.96, 170.61, 169.40, 168.49, 167.78, 146.84, 141.74, 132.65, 128.86, 128.70, 126.31, 119.76, 119.65, 110.88, 109.47, 109.44, 48.99, 41.90, 38.74, 34.45, 34.02, 31.45, 31.11, 30.89, 26.88, 26.57, 17.21, 16.14; HRMS calcd for $C_{31}H_{35}N_6O_6$: $[M+H]^+$ 587.2540, found $[M+H]^+$ 587.2609.

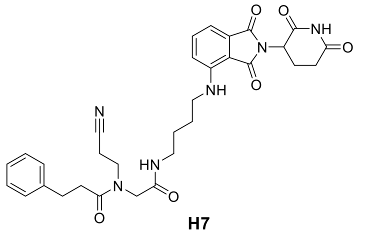

H7

J7: 2-cyclohexyl-N-(2-((4-((2-(2,6-dioxopiperidin-3-yl)-1,3-dioxoisoindolin-4-yl)amino)butyl)amino)-2-oxoethyl)-N-(2-methoxyethyl)acetamide:

Yellow solid, procedure **f**, purified yield: 43%. ^{1}H NMR (500 MHz, Chloroform-*d*) δ 8.51 (d, J = 27.2 Hz, 1H), 7.51 – 7.45 (m, 1H), 7.08 (dd, J = 7.1, 5.6 Hz, 1H), 6.87 (d, J = 8.5 Hz, 1H), 6.78 (t, J = 5.9 Hz, 1H), 6.26 – 6.16 (m, 1H), 4.96 – 4.86 (m, 1H), 3.96 (s, 2H), 3.66 – 3.54 (m, 3H), 3.49 (t, J = 5.4 Hz, 1H), 3.36 – 3.23 (m, 7H), 2.91 – 2.68 (m, 3H), 2.28 (d, J = 6.9 Hz, 1H), 2.15 – 2.04 (m, 2H), 1.89 – 1.75 (m, 2H), 1.72 – 1.57 (m, 9H), 1.32 – 1.20 (m, 3H), 1.17 – 1.03 (m, 1H); ^{13}C NMR (126 MHz, $CDCl_3$) δ 174.16, 171.22, 169.90, 169.46, 168.50, 167.63, 146.86, 136.19, 132.47, 116.70, 111.50, 109.94, 69.95, 58.99, 58.55, 54.31, 51.76, 49.57, 48.88, 42.19, 40.96, 40.43, 38.92, 34.94, 33.31, 31.44, 26.95, 26.49, 26.19, 22.81; HRMS calcd for $C_{30}H_{42}N_5O_7$: $[M+H]^+$ 584.3006, found $[M+H]^+$ 584.3074.

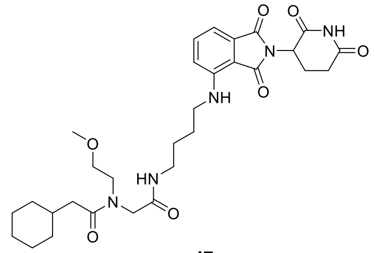

J7

K7: 2-(bicyclo[2.2.1]heptan-2-yl)-N-(2-((4-((2-(2,6-dioxopiperidin-3-yl)-1,3-dioxoisoindolin-4-yl)amino)butyl)amino)-2-oxoethyl)-N-(4-sulfamoylphenethyl)acetamide:

Yellow solid, procedure **f**, purified yield: 38%. ^{1}H NMR (500 MHz, DMSO-d_6) δ 11.11 (s, 1H), 8.06 (t, J = 5.7 Hz, 1H), 7.80 – 7.72 (m, 2H), 7.60 – 7.54 (m, 1H), 7.44 (d, J = 7.9 Hz, 1H), 7.39 (d, J = 7.9 Hz, 1H), 7.30 (s, 2H), 7.14 – 7.08 (m, 1H), 7.03 (d, J = 7.0 Hz, 1H), 6.55 (t, J = 5.8 Hz, 1H), 5.12 – 5.01 (m, 1H), 3.95 – 3.81 (m, 2H), 3.58 – 3.50 (m, 1H), 3.50 – 3.44 (m, 1H), 3.31 (q, J = 6.5 Hz, 2H), 3.18 – 3.07 (m, 2H), 2.95 – 2.84 (m, 2H), 2.80 (t, J = 7.7 Hz, 1H), 2.64 – 2.54 (m, 2H), 2.20 – 2.08 (m, 2H), 2.07 – 1.90 (m, 2H), 1.84 (d, J = 18.1 Hz, 1H), 1.80 – 1.68 (m, 1H), 1.58 (t, J = 7.3 Hz, 2H), 1.49 (q, J = 7.9 Hz, 2H), 1.44 – 1.29 (m, 3H), 1.25 – 1.14 (m, 1H), 1.13 – 1.02 (m, 2H), 1.01 – 0.80 (m, 2H); ^{13}C NMR (126 MHz, DMSO) δ 173.30, 172.51, 172.16, 170.58, 170.56, 169.41, 168.78, 167.78, 146.87, 144.04, 143.48, 142.75, 142.46, 132.65, 126.31, 126.08, 117.69, 110.88, 109.47, 49.02, 49.00, 42.01, 38.74, 38.57, 38.29, 38.04, 36.61, 36.55, 35.38, 34.35, 33.46, 31.45, 29.95, 28.74, 26.95, 22.58.; HRMS calcd for $C_{36}H_{45}N_6O_8S$: $[M+H]^+$ 721.2941, found

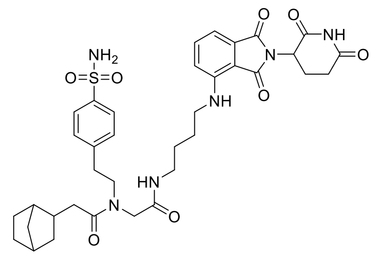

K7

$[M+H]^+$ 721.3011.

C9: N-(2-((4-((2-(2,6-dioxopiperidin-3-yl)-1,3-dioxoisoindolin-4-yl)amino)butyl)amino)-2-oxo-1-(4-(trifluoromethyl)phenyl)ethyl)-N-propylbutyramide:

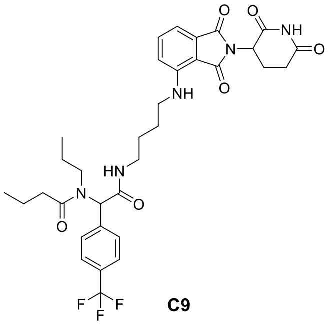

C9

Yellow solid, procedure **e**, purified yield: 60%. ^{1}H NMR (500 MHz, Chloroform-*d*) δ 8.94 (d, J = 8.5 Hz, 1H), 7.58 (d, J = 8.1 Hz, 2H), 7.53 – 7.38 (m, 3H), 7.04 (d, J = 7.0 Hz, 1H), 6.83 (d, J = 8.6 Hz, 1H), 6.75 (t, J = 5.8 Hz, 1H), 6.19 (t, J = 5.8 Hz, 1H), 5.79 (s, 1H), 5.05 – 4.76 (m, 1H), 3.37 – 3.13 (m, 6H), 2.85 – 2.77 (m, 1H), 2.75 – 2.70 (m, 1H), 2.44 – 2.26 (m, 2H), 2.20 – 1.89 (m, 2H), 1.72 – 1.54 (m, 6H), 1.53 – 1.42 (m, 1H), 1.21 – 1.08 (m, 1H), 0.94 (t, J = 7.4 Hz, 3H), 0.73 (t, J = 7.4 Hz, 3H); ^{13}C NMR (126 MHz, $CDCl_3$) δ 174.44, 171.57, 169.69, 169.52, 168.84, 167.64, 146.80, 139.80, 136.14, 132.45, 130.26 (q, J=32.7 Hz), 129.07, 125.54 (d, J = 4.0 Hz), 123.94 (q, J = 272.3 Hz), 116.6 (d, J = 3.7 Hz), 111.46, 109.93, 62.67, 49.44, 48.89, 42.15, 39.13, 35.33, 31.43, 26.73, 26.53, 23.08, 22.76, 18.76, 13.91, 11.18; HRMS calcd for $C_{33}H_{39}F_3N_5O_6$: $[M+H]^+$ 658.2774, found $[M+H]^+$ 658.2844.

F9: N-(2-((4-((2-(2,6-dioxopiperidin-3-yl)-1,3-dioxoisoindolin-4-yl)amino)butyl)amino)-2-oxoethyl)-N-(4-methoxyphenethyl)-1-methylcyclohexane-1-carboxamide:

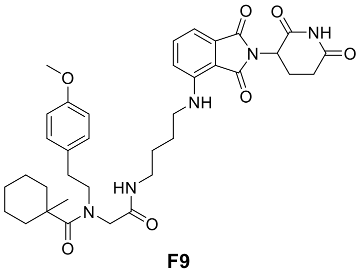

F9

Yellow solid, procedure **f**, purified yield: 53%. ^{1}H NMR (500 MHz, Chloroform-*d*) δ 8.98 (s, 1H), 7.48 – 7.42 (m, 1H), 7.12 – 7.04 (m, 3H), 6.88 – 6.82 (m, 3H), 6.73 (s, 1H), 6.23 (q, J = 5.8, 4.9 Hz, 1H), 4.96 – 4.88 (m, 1H), 4.00 (s, 2H), 3.79 (s, 3H), 3.69 – 3.57 (m, 2H), 3.31 – 3.22 (m, 4H), 2.87 – 2.82 (m, 2H), 2.80 – 2.72 (m, 2H), 2.18 – 1.96 (m, 4H), 1.70 – 1.52 (m, 6H), 1.51 – 1.43 (m, 3H), 1.40 – 1.30 (m, 3H), 1.22 (s, 3H); ^{13}C NMR (126 MHz, $CDCl_3$) δ 178.16, 171.55, 169.48, 168.80, 167.64, 158.37, 146.79, 136.16, 132.45, 129.71, 116.67, 114.13, 114.10, 111.46, 109.92, 55.28, 53.06, 52.08, 48.89, 43.20, 42.14, 38.81, 37.14, 31.46, 26.99, 26.52, 25.85, 23.26, 22.77; HRMS calcd for $C_{36}H_{46}N_5O_7$: $[M+H]^+$ 660.3319, found $[M+H]^+$ 660.3389.

N9: 4-((4-(5-(((3,4-dimethylphenyl)amino)(pyridin-3-yl)methyl)-1H-tetrazol-1-yl)butyl)amino)-2-(2,6-dioxopiperidin-3-yl)isoindoline-1,3-dione:

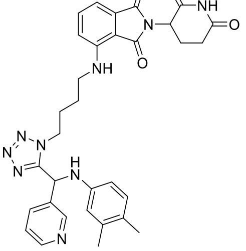

N9

Yellow solid, procedure **g**, purified yield 67%. ^{1}H NMR (500 MHz, Chloroform-*d*) δ 9.23 (d, J = 12.0 Hz, 1H), 8.70 (s, 1H), 8.60 – 8.45 (m, 1H), 7.80 – 7.71 (m, 1H), 7.43 (t, J = 7.8 Hz, 1H), 7.06 (d, J = 7.1 Hz, 1H), 6.87 (dd, J = 8.2, 1.8 Hz, 1H), 6.73 (d, J = 8.6 Hz, 1H), 6.49 (d, J = 2.5 Hz, 1H), 6.40 (dd, J = 8.1, 2.5 Hz, 1H), 6.20 (t, J = 5.8 Hz, 1H), 5.95 (d, J = 7.3 Hz, 1H), 4.90 (q, J = 5.8, 5.2 Hz, 2H), 4.37 (t, J = 7.3 Hz, 2H), 3.21 – 3.08 (m, 2H), 2.88 – 2.62 (m, 3H), 2.10 (d, J = 8.0 Hz, 7H), 1.94 (s, 1H),

1.92 – 1.80 (m, 2H), 1.59 – 1.49 (m, 2H); ^{13}C NMR (126 MHz, $CDCl_3$) δ 171.57, 169.45, 168.86, 167.50, 154.78, 149.91, 148.52, 146.41, 143.00, 142.98, 137.76, 136.15, 135.15, 133.50, 132.39, 130.43, 127.91, 124.19, 116.42, 116.03, 111.62, 111.33, 110.08, 77.26, 77.00, 76.75, 51.40, 48.88, 47.37, 41.62, 31.34, 26.73, 25.89, 22.71, 19.95, 18.68; HRMS calcd for $C_{32}H_{34}N_9O_4$: $[M+H]^+$ 608.2656, found $[M+H]^+$ 608.2722.

O9: 4-((4-(5-(4-((3,4-dimethylphenyl)amino)-1-methylpiperidin-4-yl)-1H-tetrazol-1-yl)butyl)amino)-2-(2,6-dioxopiperidin-3-yl)isoindoline-1,3-dione:

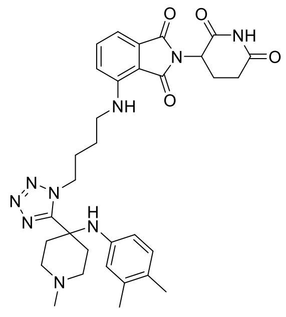

O9

Yellow solid, procedure **g**, purified yield 70%. ^{1}H NMR (500 MHz, Chloroform-*d*) δ 7.47 (dd, J = 8.5, 7.1 Hz, 1H), 7.10 (d, J = 7.0 Hz, 1H), 6.76 (t, J = 8.4 Hz, 2H), 6.17 (t, J = 5.8 Hz, 1H), 6.12 (d, J = 2.5 Hz, 1H), 5.91 (dd, J = 8.1, 2.5 Hz, 1H), 4.92 (dd, J = 12.1, 5.4 Hz, 1H), 4.64 – 4.50 (m, 2H), 4.09 (s, 1H), 3.14 (q, J = 6.5 Hz, 2H), 2.90 – 2.66 (m, 5H), 2.54 – 2.44 (m, 2H), 2.44 – 2.34 (m, 2H), 2.29 (s, 3H), 2.27 – 2.19 (m, 2H), 2.18 – 1.99 (m, 8H), 1.99 – 1.88 (m, 2H), 1.67 – 1.50 (m, 2H); ^{13}C NMR (126 MHz, $CDCl_3$) δ 171.52, 169.55, 168.85, 167.49, 158.32, 146.55, 141.62, 137.64, 136.15, 132.43, 130.36, 127.58, 116.98, 116.42, 112.41, 111.65, 110.02, 77.26, 77.00, 76.74, 51.86, 50.53, 48.91, 48.16, 45.73, 41.77, 34.20, 34.05, 31.41, 26.90, 25.92, 22.80, 19.92, 18.60; HRMS calcd for $C_{32}H_{40}N_9O_4$: $[M+H]^+$ 614.3125, found $[M+H]^+$ 614.3192.

F11: 2-(bicyclo[2.2.1]heptan-2-yl)-N-(2-((4-((2-(2,6-dioxopiperidin-3-yl)-1,3-dioxoisoindolin-4-yl)amino)butyl)amino)-2-oxoethyl)-N-propylacetamide:

Yellow solid, procedure **f**, purified yield: 32%. ^{1}H NMR (500 MHz, Chloroform-*d*) δ 8.38 (s, 1H), 7.56 – 7.47 (m, 1H), 7.11 (d, J = 7.1 Hz, 1H), 6.90 (d, J = 8.5 Hz, 1H), 6.85 (t, J = 6.0 Hz, 1H), 6.24 (t, J = 5.7 Hz, 1H), 4.98 – 4.89 (m, 1H), 3.99 – 3.90 (m, 2H), 3.36 – 3.24 (m, 6H), 2.94 – 2.71 (m, 3H), 2.40 – 2.30 (m, 1H), 2.25 – 2.12 (m, 3H), 2.01 – 1.92 (m, 2H), 1.74 – 1.46 (m, 11H), 1.18 – 1.11 (m, 2H), 1.08 – 1.01 (m, 1H), 0.93 (t, J = 7.4 Hz, 3H); ^{13}C NMR (126 MHz, $CDCl_3$) δ 173.84, 171.13, 170.12, 169.47, 168.43, 167.63, 146.85, 136.20, 132.48, 116.69, 111.54, 109.95, 51.56, 48.88, 42.20, 41.09, 39.58, 38.76, 38.58, 38.02, 36.77, 35.39, 31.45, 29.85, 29.78, 28.54, 26.96, 26.55, 22.81, 21.97, 11.13; HRMS calcd for $C_{31}H_{42}N_5O_6$: $[M+H]^+$ 580.3057, found $[M+H]^+$ 580.3125.

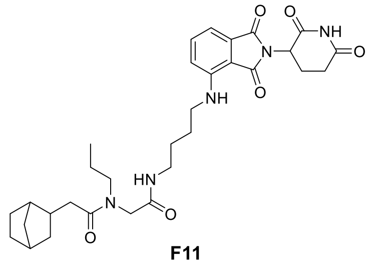

F11

K11: 2-cyclohexyl-N-(2-((4-((2-(2,6-dioxopiperidin-3-yl)-1,3-dioxoisoindolin-4-yl)amino)butyl)amino)-2-oxoethyl)-N-(4-(trifluoromethyl)benzyl)acetamide:

Yellow solid, procedure **f**, purified yield: 53%. ^{1}H NMR (500 MHz, Chloroform-$_{d}$) δ 8.71 (d, J = 79.1 Hz, 1H), 7.67 – 7.55 (m, 2H), 7.51 – 7.45 (m, 1H), 7.38 – 7.27 (m, 2H), 7.08 (t, J = 7.1 Hz, 1H), 6.90 – 6.83 (m, 1H), 6.62 (t, J = 6.0 Hz, 1H), 6.23 – 6.17 (m, 1H), 4.96 – 4.88 (m, 1H), 4.78 – 4.60 (m, 2H), 3.91 (s, 2H), 3.35 – 3.12 (m, 4H), 2.92 – 2.67 (m, 3H), 2.25 (d, J = 6.9 Hz, 1H), 2.21 – 2.16 (m, 1H), 2.14 – 2.08 (m, 1H), 1.92 – 1.82 (m, 1H), 1.76 – 1.43 (m, 11H), 1.16 – 1.05 (m, 1H), 1.01 – 0.89 (m, 2H); ^{13}C NMR (126 MHz, $CDCl_3$) δ 174.14, 171.32, 169.52, 169.09, 168.65, 167.61, 146.82, 140.16, 136.23 (d, J = 6.0 Hz), 132.48, 130.27 (q, J = 32.9 Hz), 128.70, 126.84, 125.95 (d, J = 3.7 Hz), 123.22 (q, J= 273.4 Hz), 116.70, 111.60, 110.03, 52.21, 51.41, 50.57, 50.11, 48.92, 42.12, 40.57, 39.02, 34.94, 33.31, 33.00, 31.44, 26.89, 26.46, 26.08, 22.80; HRMS calcd for $C_{35}H_{41}F_3N_5O_6$: $[M+H]^+$ 684.2931, found $[M+H]^+$ 684.3002.

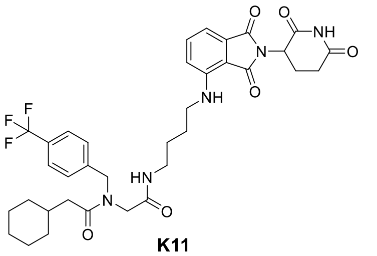

K11

M11: 4-((4-(5-(((cyclopropylmethyl)amino)(phenyl)methyl)-1H-tetrazol-1-yl)butyl)amino)-2-(2,6-dioxopiperidin-3-yl)isoindoline-1,3-dione:

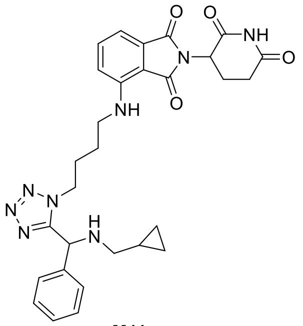

M11

Yellow solid, procedure **g**, purified yield 54%. ^{1}H NMR (500 MHz, Chloroform-*d*) δ 8.42 (s, 1H), 7.47 (dd, J = 8.5, 7.1 Hz, 1H), 7.33 (d, J = 4.4 Hz, 4H), 7.31 – 7.27 (m, 1H), 7.09 (d, J = 7.0 Hz, 1H), 6.76 (d, J = 8.5 Hz, 1H), 6.14 (t, J = 5.8 Hz, 1H), 5.31 (d, J = 0.9 Hz, 1H), 4.91 (dd, J = 12.3, 5.4 Hz, 1H), 4.36 – 4.16 (m, 2H), 3.13 (q, J = 6.7 Hz, 2H), 2.90 – 2.68 (m, 3H), 2.48 – 2.36 (m, 2H), 2.15 – 2.08 (m, 1H), 1.78 – 1.64 (m, 3H), 1.51 – 1.41 (m, 2H), 0.99 – 0.90 (m, 1H), 0.46 (dd, J = 8.1, 4.1 Hz, 2H), 0.12 – 0.07 (m, 1H), 0.07 – 0.01 (m, 1H); ^{13}C NMR (126 MHz, $CDCl_3$) δ 171.08, 169.39, 168.38, 167.44, 155.46, 146.50, 137.72, 136.15, 132.43, 129.10, 128.60, 127.16, 116.40, 111.70, 110.12, 57.18, 52.70, 48.86, 46.96, 41.73, 31.36, 26.41, 25.96, 22.73, 10.90, 3.50, 3.31; HRMS calcd for $C_{29}H_{33}N_8O_4$: $[M+H]^+$ 557.2541, found $[M+H]^+$ 557.2613.

H12: N-(2-((4-((2-(2,6-dioxopiperidin-3-yl)-1,3-dioxoisoindolin-4-yl)amino)butyl)amino)-2-oxoethyl)-N-propylpropionamide:

Yellow solid, procedure **f**, purified yield: 42%. ^{1}H NMR (500 MHz, Chloroform-*d*) δ 8.54 (s, 1H), 7.50 – 7.45 (m, 1H), 7.07 (d, J = 7.1 Hz, 1H), 6.87 (d, J = 8.6 Hz, 1H), 6.77 (t, J = 6.0 Hz, 1H), 6.21 (t, J = 5.8 Hz, 1H), 4.96 – 4.88 (m, 1H), 3.93 (s, 2H), 3.33 – 3.22 (m, 6H), 2.91 – 2.84 (m, 1H), 2.81 – 2.69 (m, 2H), 2.38 (t, J = 7.4 Hz, 1H), 2.16 – 2.08 (m, 1H), 1.83 – 1.56 (m, 7H), 1.14 (t, J = 7.4 Hz, 3H), 0.89 (t, J = 7.4 Hz, 3H); ^{13}C NMR (126 MHz, $CDCl_3$) δ 175.02, 171.22, 169.99, 169.49, 168.54, 167.62, 146.86, 136.17, 132.49, 116.69, 111.50, 109.97, 51.47, 51.41, 48.90, 42.19, 38.79, 31.45, 26.94, 26.54, 26.14, 22.80, 21.84, 11.09, 9.48; HRMS calcd for $C_{25}H_{34}N_5O_6$: $[M+H]^+$ 500.2431, found $[M+H]^+$ 500.2501.

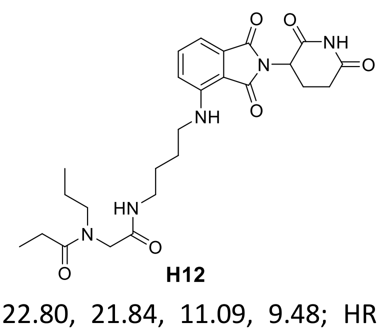

H12

G13: 2-(cyclopent-2-en-1-yl)-N-(2-((4-((2-(2,6-dioxopiperidin-3-yl)-1,3-dioxoisoindolin-4-yl)amino)butyl)amino)-2-oxoethyl)-N-(2-hydroxyethyl)acetamide:

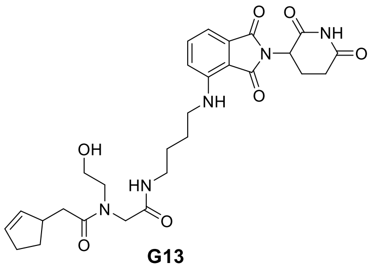

G13

Yellow solid, procedure **f**, purified yield: 35%. ^{1}H NMR (500 MHz, Chloroform-*d*) δ 8.86 (d, J = 43.5 Hz, 1H), 7.50 – 7.45 (m, 1H), 7.06 (d, J = 7.0 Hz, 1H), 6.91 – 6.82 (m, 1H), 6.75 (t, J = 5.9 Hz, 1H), 6.27 – 6.16 (m, 1H), 5.74 – 5.69 (m, 1H), 5.67 – 5.61 (m, 1H), 5.10 (t, J = 7.0 Hz, 1H), 4.99 – 4.85 (m, 1H), 4.02 – 3.84 (m, 2H), 3.79 – 3.69 (m, 2H), 3.61 – 3.43 (m, 2H), 3.32 – 3.23 (m, 4H), 3.18 – 3.08 (m, 1H), 2.89 – 2.80 (m, 1H), 2.79 – 2.69 (m, 2H), 2.50 – 2.38 (m, 1H), 2.34 – 2.22 (m, 2H), 2.15 – 2.06 (m, 2H), 1.85 (s, 1H), 1.71 – 1.55 (m, 4H), 1.46 – 1.33 (m, 1H); ^{13}C NMR (126 MHz, $CDCl_3$) δ 173.92, 171.48, 171.08, 169.56, 168.83, 167.65, 146.88, 136.23, 134.18, 132.45, 131.32, 116.80, 111.52, 109.90, 60.22, 52.93, 51.68, 48.90, 42.16, 41.92, 39.21, 31.78, 31.44, 29.91, 26.72, 26.47, 26.43, 22.81; HRMS calcd for $C_{28}H_{36}N_5O_7$: $[M+H]^+$ 554.2536, found $[M+H]^+$ 554.2604.

L13: 2-(2,6-dioxopiperidin-3-yl)-4-((4-(5-(1-((thiophen-3-ylmethyl)amino)cyclohexyl)-1H-tetrazol-1-yl)butyl)amino)isoindoline-1,3-dione:

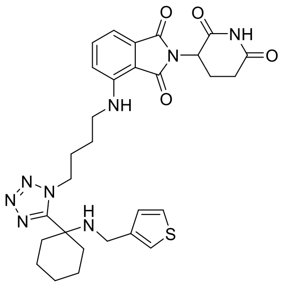

L13

Yellow solid, procedure **g**, purified yield 19%. ^{1}H NMR (500 MHz, Chloroform-*d*) δ 8.31 (s, 1H), 7.49 (dd, J = 8.5, 7.1 Hz, 1H), 7.26 (d, J = 3.0 Hz, 1H), 7.10 (d, J = 7.1 Hz, 1H), 7.06 (dd, J = 2.9, 1.3 Hz, 1H), 6.91 (dd, J = 4.9, 1.3 Hz, 1H), 6.83 (d, J = 8.6 Hz, 1H), 6.23 (t, J = 5.8 Hz, 1H), 4.92 (dd, J = 12.2, 5.4 Hz, 1H), 4.74 (t, J = 7.6 Hz, 2H), 3.43 (s, 2H), 3.25 (q, J = 6.7 Hz, 2H), 2.95 – 2.69 (m, 3H), 2.20 – 2.09 (m, 3H), 2.08 – 1.96 (m, 4H), 1.88 – 1.64 (m, 6H), 1.63 – 1.50 (m, 3H), 1.48 – 1.33 (m, 1H); ^{13}C NMR (126 MHz, $CDCl_3$) δ 171.08, 169.40, 168.35, 167.47, 157.90, 146.59, 140.53, 136.20, 132.44, 126.97, 126.18, 120.86, 116.46, 111.69, 110.08, 55.53, 48.82, 48.23, 41.99, 41.91, 34.59, 31.35, 27.29, 26.45, 25.01, 22.72, 21.39; HRMS calcd for $C_{29}H_{35}N_8O_4S$: $[M+H]^+$ 591.2424, found $[M+H]^+$ 591.2493.

E14: N-(4-((2-(2,6-dioxopiperidin-3-yl)-1,3-dioxoisoindolin-4-yl)amino)butyl)-2-(N-isobutylformamido)-2-(3-methoxyphenyl)acetamide:

Yellow solid, procedure **e**, purified yield: 37%. ^{1}H NMR (500 MHz, Chloroform-*d*) δ 8.44 (d, J = 8.6 Hz, 1H), 8.16 (d, J = 24.3 Hz, 1H), 7.48 – 7.43 (m, 1H), 7.28 (d, J = 6.6 Hz, 1H), 7.07 (t, J = 7.8 Hz, 1H), 6.99 – 6.93 (m, 2H), 6.90 – 6.83 (m, 2H), 6.29 (t, J = 5.9 Hz, 1H), 6.20 (t, J = 5.7 Hz, 1H), 5.43 (s, 1H), 4.98 – 4.88 (m, 1H), 3.77 (s, 3H), 3.36 – 3.22 (m, 4H), 3.19 – 3.10 (m, 1H), 3.04 – 2.97 (m, 1H), 2.88 – 2.83 (m, 1H), 2.77 – 2.71 (m, 1H), 2.14 – 2.07 (m, 1H), 1.75 (s, 1H), 1.66 – 1.57 (m, 4H), 1.53 – 1.43 (m, 1H), 0.93 – 0.71 (m, 6H); ^{13}C NMR (126 MHz, $CDCl_3$) δ 171.23, 169.49, 169.21, 168.53, 167.63, 163.98, 159.92, 146.86, 136.21, 135.89, 132.46, 129.95, 121.33, 116.73, 114.80, 114.20, 111.58, 109.94, 62.29, 55.73, 55.35, 48.88, 42.18, 39.24, 31.42, 27.37, 26.76, 26.51, 22.80, 19.82, 19.66; HRMS calcd for $C_{31}H_{38}N_5O_7$: $[M+H]^+$ 592.2693, found $[M+H]^+$ 592.2759.

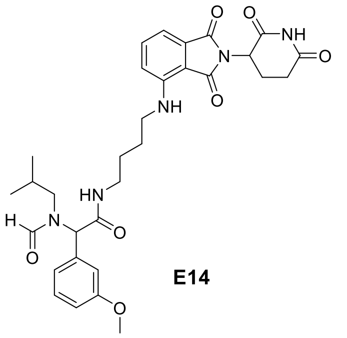

E14

L18: 4-((4-(5-(1-((2,2-dimethoxyethyl)amino)cyclohexyl)-1H-tetrazol-1-yl)butyl)amino)-2-(2,6-dioxopiperidin-3-yl)isoindoline-1,3-dione:

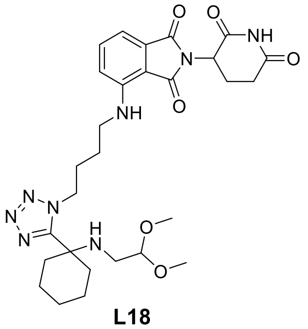

L18

Yellow solid, procedure **g**, purified yield 37%. ^{1}H NMR (500 MHz, Chloroform-*d*) δ 8.31 (s, 1H), 7.51 (dd, J = 8.5, 7.1 Hz, 1H), 7.11 (d, J = 7.1 Hz, 1H), 6.89 (d, J = 8.5 Hz, 1H), 6.28 (t, J = 5.8 Hz, 1H), 4.92 (dd, J = 12.3, 5.4 Hz, 1H), 4.74 (t, J = 7.4 Hz, 2H), 4.29 (t, J = 5.0 Hz, 1H), 3.39 – 3.33 (m, 2H), 3.31 (s, 6H), 2.91 – 2.80 (m, 1H), 2.79 – 2.73 (m, 1H), 2.34 (d, J = 5.0 Hz, 2H), 2.18 – 2.03 (m, 5H), 1.92 (dd, J = 13.5, 5.2 Hz, 2H), 1.81 (q, J = 7.4 Hz, 4H), 1.67 – 1.50 (m, 5H), 1.46 – 1.33 (m, 1H); ^{13}C NMR (126 MHz, $CDCl_3$) δ 171.04, 169.41, 168.33, 167.48, 157.96, 146.63, 136.21, 132.47, 116.48, 111.71, 110.16, 103.79, 55.08, 53.88, 48.85, 48.09, 43.84, 42.06, 34.63, 31.37, 27.17, 26.55, 25.04, 22.73, 21.39; HRMS calcd for $C_{28}H_{39}N_8O_6$: $[M+H]^+$ 583.2914, found $[M+H]^+$ 583.2982.

I19: N-(2-((4-((2-(2,6-dioxopiperidin-3-yl)-1,3-dioxoisoindolin-4-yl)amino)butyl)amino)-2-oxoethyl)-N-(2-hydroxyethyl)-2-methoxybenzamide:

Yellow solid, procedure **f**, purified yield: 28%. ^{1}H NMR (500 MHz, Chloroform-*d*) δ 8.53 (s, 1H), 7.53 – 7.45 (m, 1H), 7.40 – 7.34 (m, 1H), 7.28 – 7.21 (m, 1H), 7.14 – 7.07 (m, 1H), 7.04 – 6.98 (m, 1H), 6.96 – 6.87 (m, 3H), 6.29 – 6.21 (m, 1H), 4.98 – 4.90 (m, 1H), 4.57 (s, 1H), 4.26 – 4.03 (m, 2H), 3.84 (s, 3H), 3.64 (t, J = 24.9 Hz, 2H), 3.49 (d, J = 18.4 Hz, 1H), 3.37 (t, J = 6.2 Hz, 1H), 3.31 (p, J = 6.1 Hz, 2H), 2.91 – 2.69 (m, 3H), 2.18 – 2.08 (m, 1H), 1.82 – 1.56 (m, 6H); ^{13}C NMR (126 MHz, $CDCl_3$) δ 171.29, 170.85, 170.36, 169.54, 168.62, 167.64, 154.89, 146.86, 136.25, 132.43, 130.77, 128.34, 125.00, 121.18, 116.82, 111.55, 110.97, 109.88, 60.43, 55.68, 53.56, 50.72, 48.88, 42.16, 39.21, 31.42, 26.82, 26.42, 22.80; HRMS calcd for $C_{29}H_{34}N_5O_8$: $[M+H]^+$ 580.2329, found $[M+H]^+$ 580.2397.

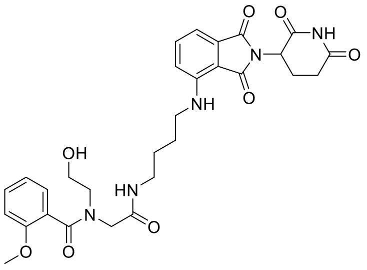

I19

H23: 2-(cyclopent-2-en-1-yl)-N-(2-((4-((2-(2,6-dioxopiperidin-3-yl)-1,3-dioxoisoindolin-4-yl)amino)butyl)amino)-2-oxoethyl)-N-(4-sulfamoylphenethyl)acetamide:

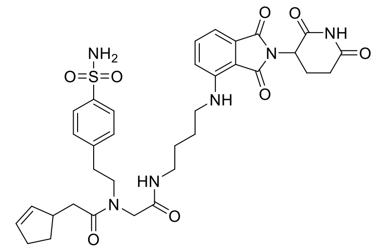

H23

Yellow solid, procedure **f**, purified yield: 36%. ^{1}H NMR (500 MHz, Acetone-d_6) δ 9.98 (s, 1H), 7.86 – 7.80 (m, 2H), 7.61 – 7.55 (m, 1H), 7.50 – 7.46 (m, 1H), 7.43 (d, J = 8.1 Hz, 1H), 7.12 (t, J = 9.2 Hz, 1H), 7.04 (dd, J = 7.1, 3.7 Hz, 1H), 6.58 (s, 1H), 6.43 (q, J = 6.5 Hz, 1H), 5.69 (d, J = 8.4 Hz, 2H), 5.12 – 5.06 (m, 1H), 4.06 – 3.97 (m, 2H), 3.71 (td, J = 7.3, 2.2 Hz, 1H), 3.61 (q, J = 7.6 Hz, 1H), 3.41 (q, J = 6.7 Hz, 2H), 3.35 – 3.25 (m, 2H), 3.04 (t, J = 7.5 Hz, 2H), 2.99 – 2.92 (m, 2H), 2.87 (s, 1H), 2.83 – 2.78 (m, 1H), 2.76 (t, J = 1.9 Hz, 1H), 2.39 – 2.18 (m, 5H), 1.76 – 1.69 (m, 2H), 1.68 – 1.58 (m, 2H), 1.42 – 1.30 (m, 1H); ^{13}C NMR (126 MHz, Acetone) δ 172.08, 171.87, 169.44, 168.69, 168.30, 167.41, 146.89, 144.18, 143.40, 142.36, 136.11, 134.90, 132.72, 130.28, 129.48, 129.24, 126.23, 116.80, 110.40, 109.90, 51.26, 50.15, 48.98, 41.94, 38.91, 38.53, 38.33, 34.42, 33.42, 31.41, 31.13, 26.89, 26.39, 22.55; HRMS calcd for $C_{34}H_{41}N_6O_8S$: $[M+H]^+$ 693.2628, found $[M+H]^+$ 693.2697.

P24: 2-(2,6-dioxopiperidin-3-yl)-4-((4-(5-(1-((furan-2-ylmethyl)amino)cyclopentyl)-1H-tetrazol-1-yl)butyl)amino)isoindoline-1,3-dione:

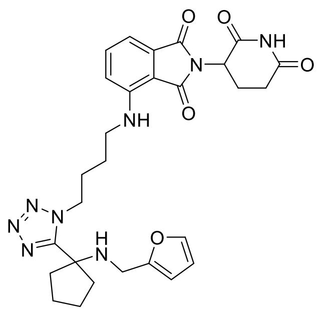

P24

Yellow solid, procedure **g**, purified yield 70%. ^{1}H NMR (500 MHz, Chloroform-d) δ 8.46 (s, 1H), 7.47 (dd, J = 8.5, 7.1 Hz, 1H), 7.25 (d, J = 1.0 Hz, 1H), 7.08 (d, J = 7.1 Hz, 1H), 6.84 (d, J = 8.5 Hz, 1H), 6.25 (t, J = 5.8 Hz, 1H), 6.22 (dd, J = 3.2, 1.9 Hz, 1H), 5.98 (dd, J = 3.2, 0.8 Hz, 1H), 4.96 – 4.80 (m, 1H), 4.63 (t, J = 7.5 Hz, 2H), 3.43 (s, 2H), 3.30 (q, J = 6.8 Hz, 2H), 2.88 – 2.66 (m, 3H), 2.38 – 2.26 (m, 2H), 2.14 – 1.98 (m, 5H), 1.83 – 1.66 (m, 7H); ^{13}C NMR (126 MHz, $CDCl_3$) δ 171.18, 169.41, 168.43, 167.46, 157.67, 152.86, 146.63, 141.85, 136.13, 132.47, 116.46, 111.61, 110.26, 110.11, 106.73, 63.73, 48.85, 47.92, 41.97, 41.09, 37.30, 31.33, 26.88, 26.38, 23.24, 22.70; HRMS calcd for $C_{28}H_{33}N_8O_5$: $[M+H]^+$ 561.2496, found $[M+H]^+$ 561.2562.

^{1}H, ^{13}C NMR spectra of mmol scale reaction

5: N-(4-((2-(2,6-dioxopiperidin-3-yl)-1,3-dioxoisoindolin-4-yl)amino)butyl)formamide:

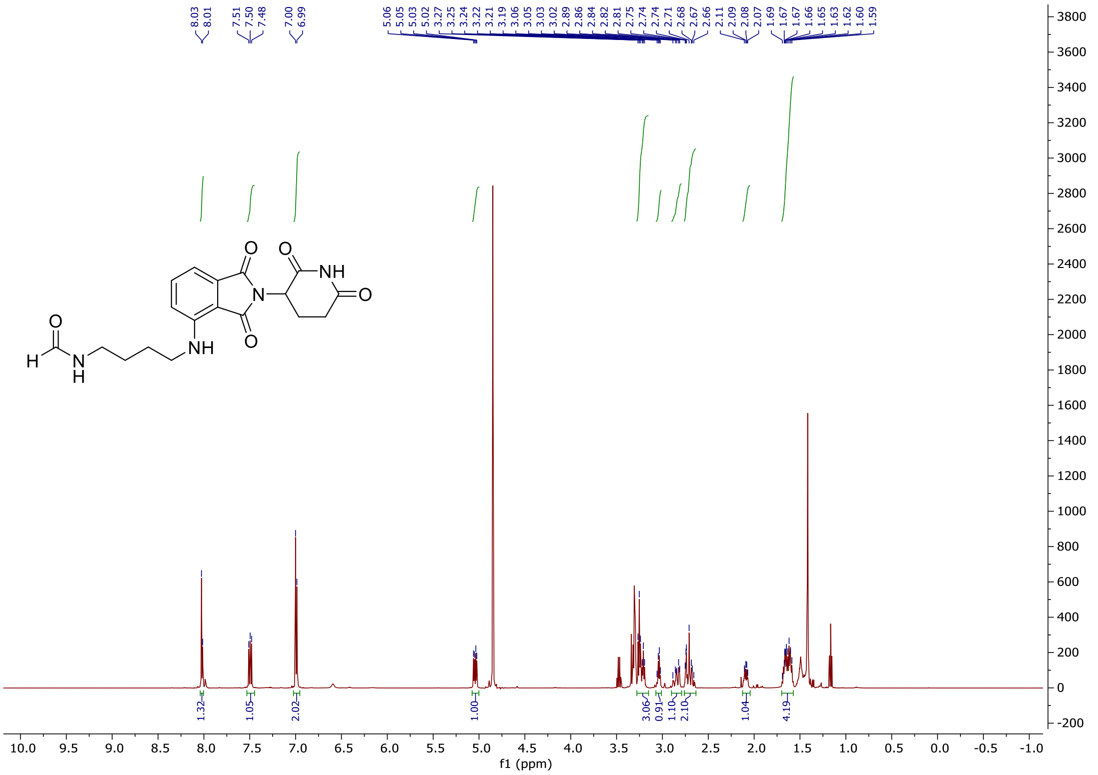

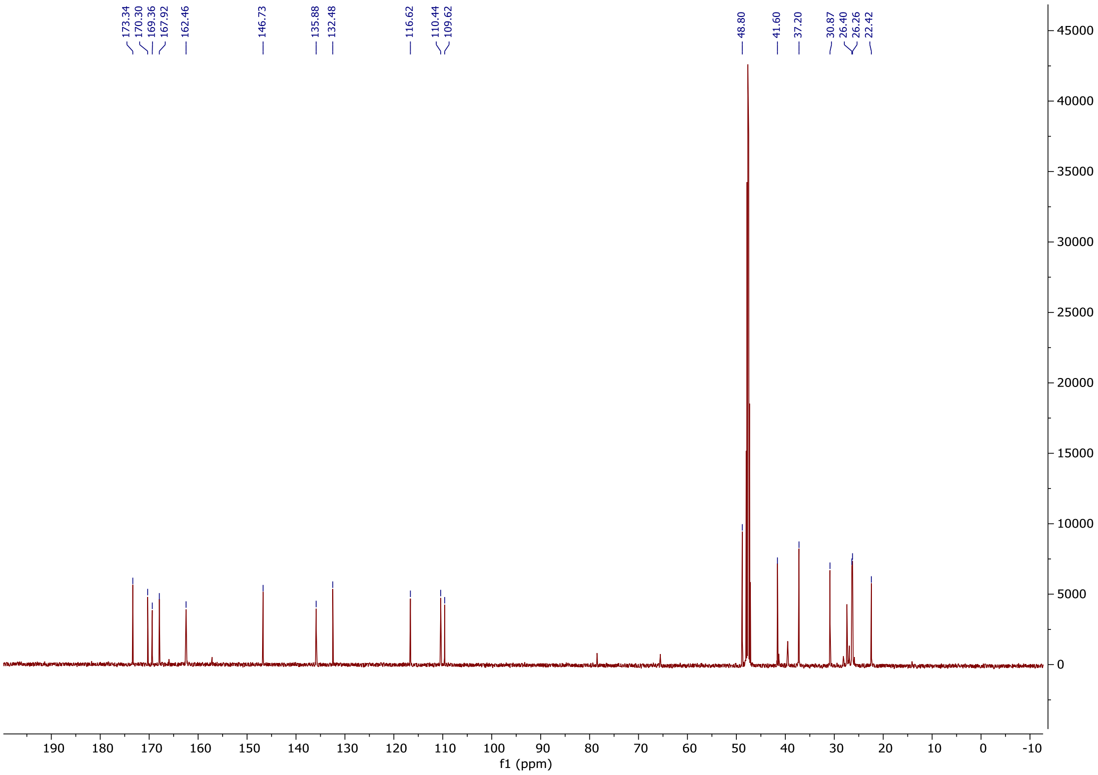

6: 2-(2,6-dioxopiperidin-3-yl)-4-((4-isocyanobutyl)amino)isoindoline-1,3-dione:

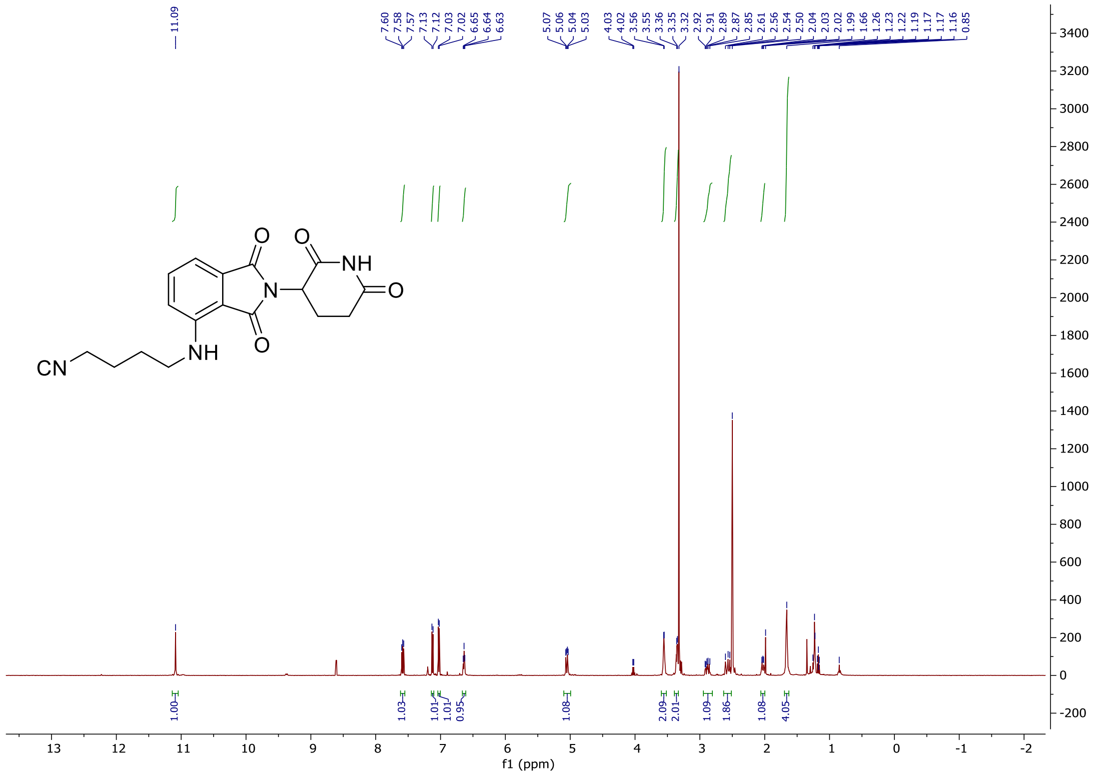

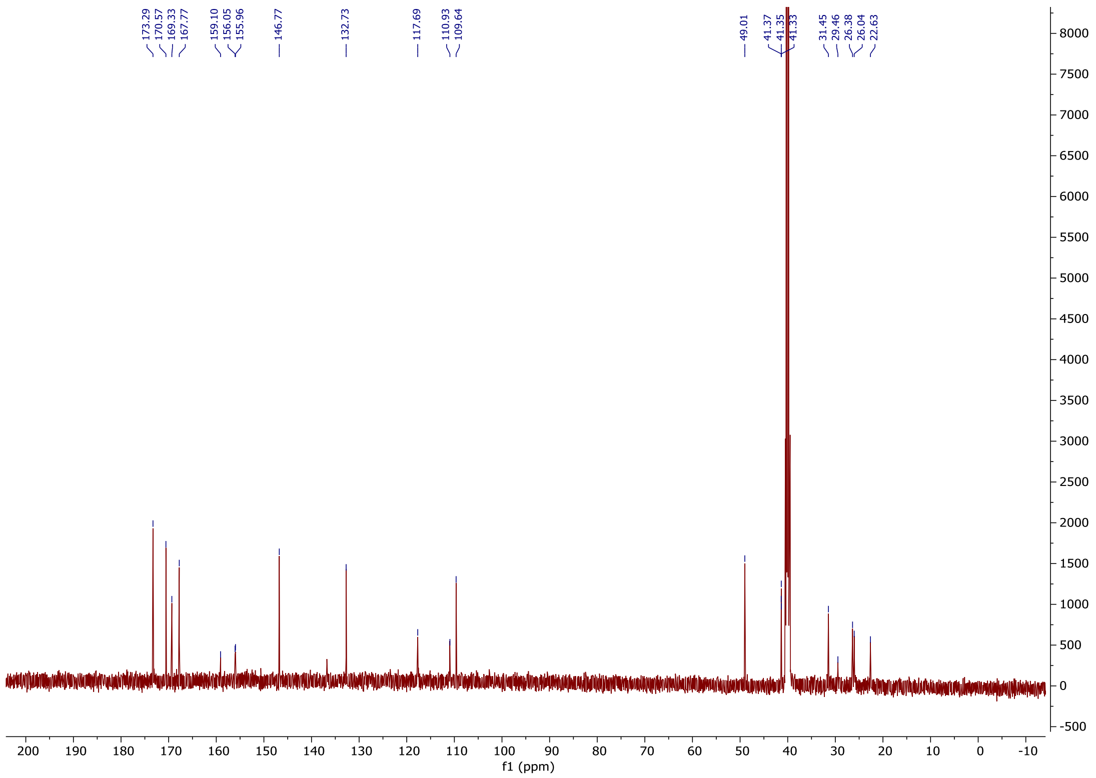

G7: 2-(bicyclo[2.2.1]heptan-2-yl)-N-(4-chlorophenyl)-N-(2-((4-((2-(2,6-dioxopiperidin-3-yl)-1,3-dioxoisoindolin-4-yl)amino)butyl)amino)-2-oxoethyl)acetamide:

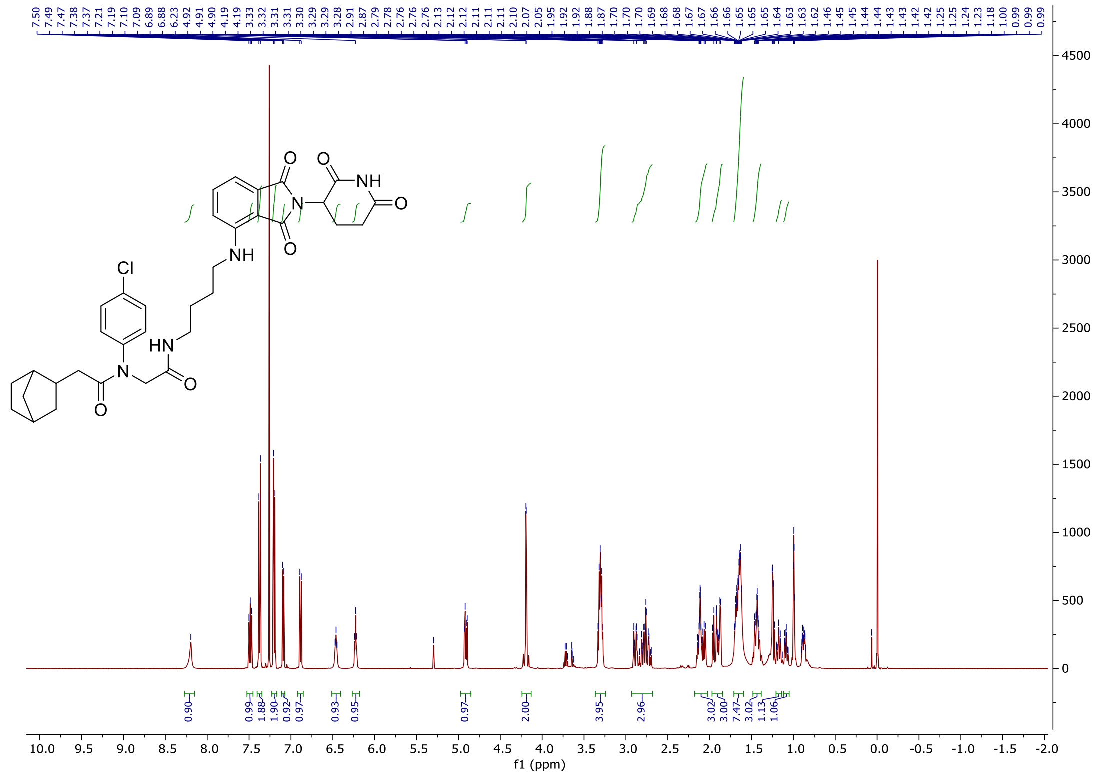

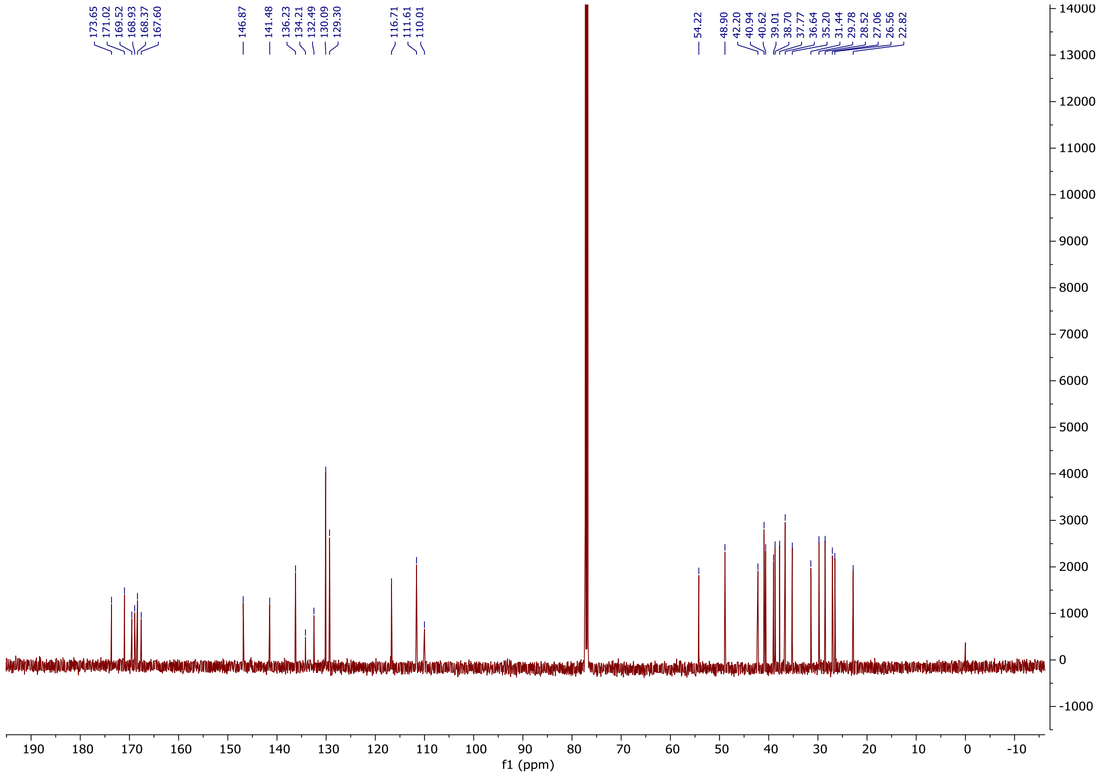

H7: N-(cyanomethyl)-N-(2-((4-((2-(2,6-dioxopiperidin-3-yl)-1,3-dioxoisoindolin-4-yl)amino)butyl)amino)-2-oxoethyl)-3-phenylpropanamide:

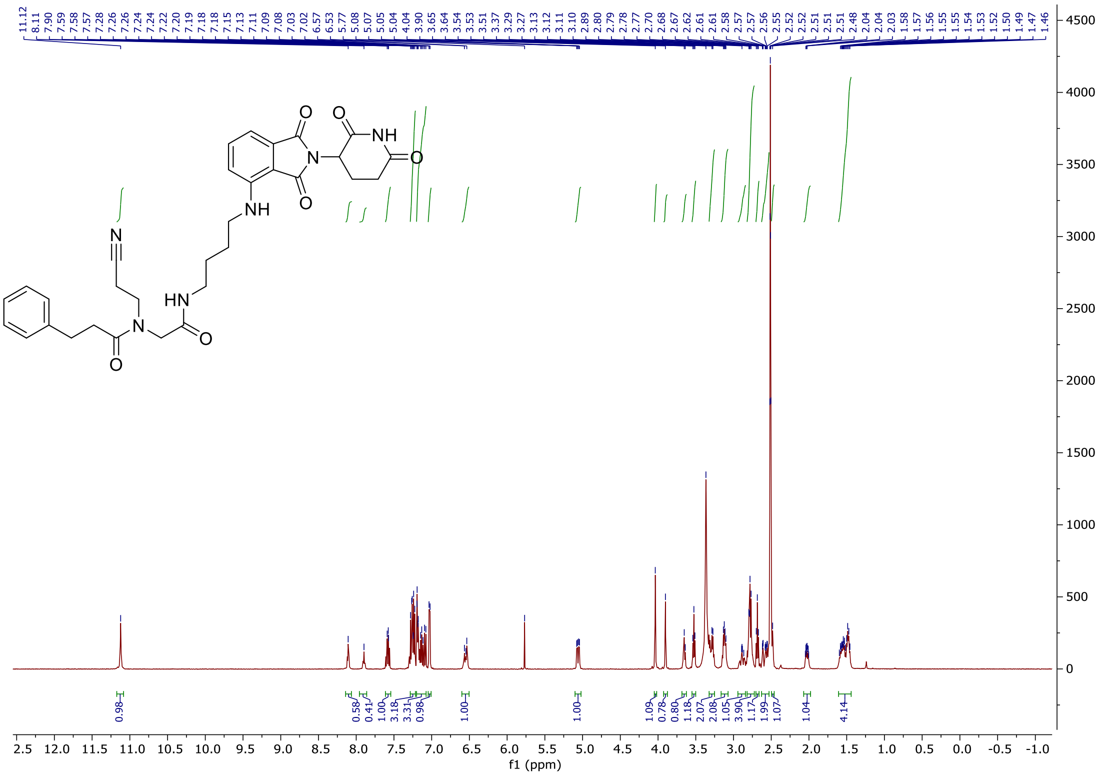

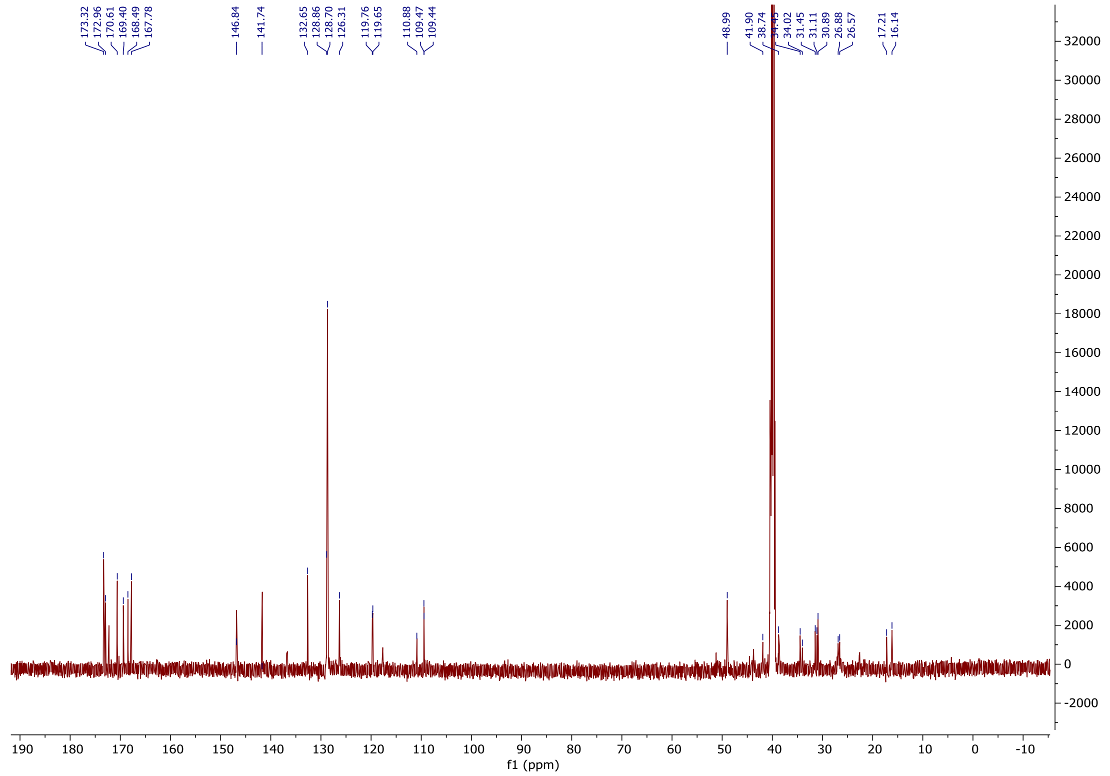

J7: 2-cyclohexyl-N-(2-((4-((2-(2,6-dioxopiperidin-3-yl)-1,3-dioxoisoindolin-4-yl)amino)butyl)amino)-2-oxoethyl)-N-(2-methoxyethyl)acetamide:

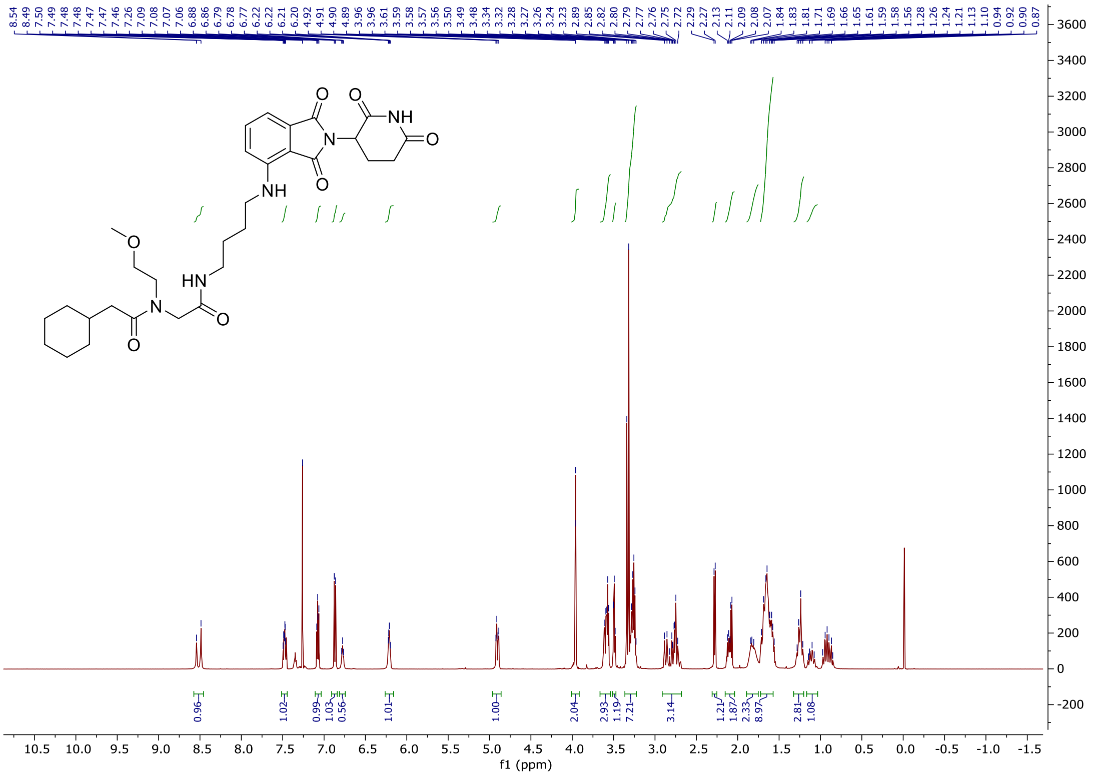

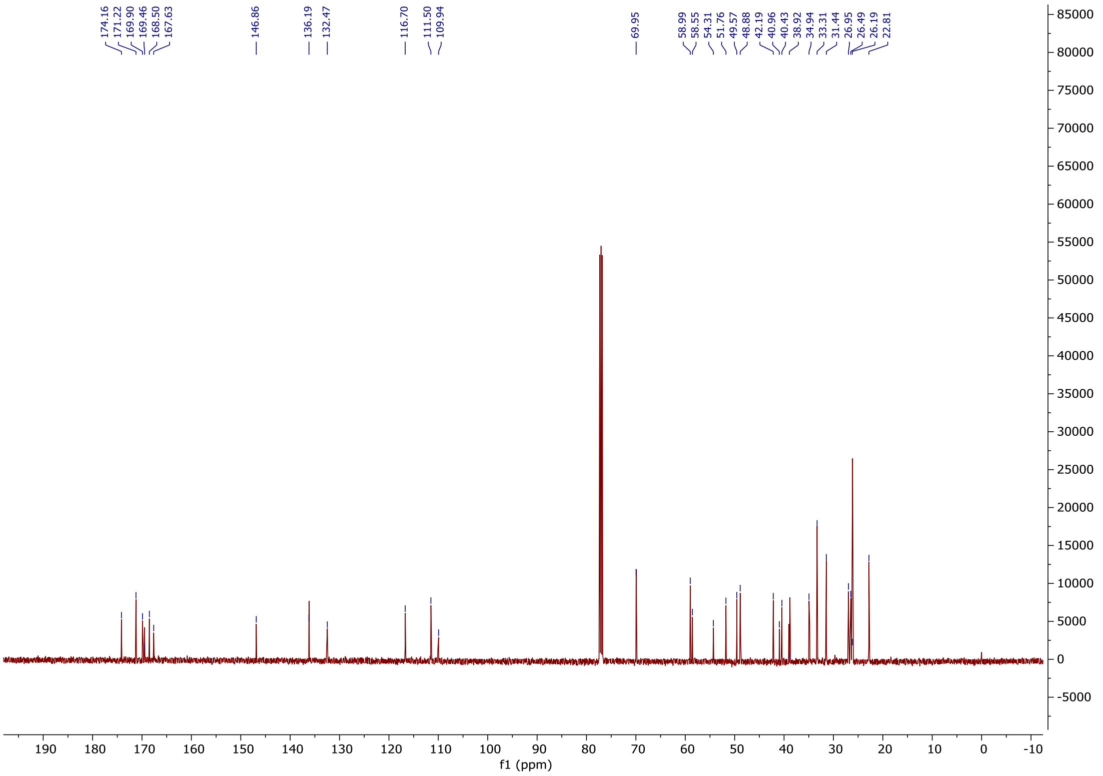

K7: 2-(bicyclo[2.2.1]heptan-2-yl)-N-(2-((4-((2-(2,6-dioxopiperidin-3-yl)-1,3-dioxoisoindolin-4-yl)amino)butyl)amino)-2-oxoethyl)-N-(4-sulfamoylphenethyl)acetamide:

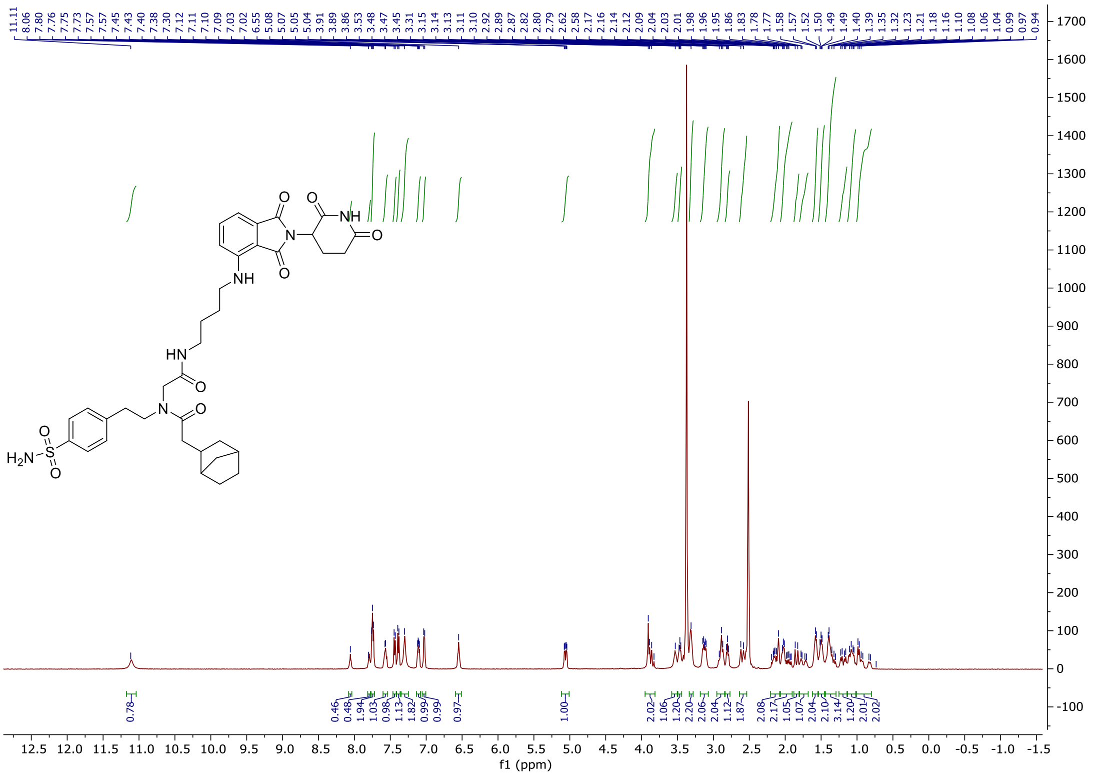

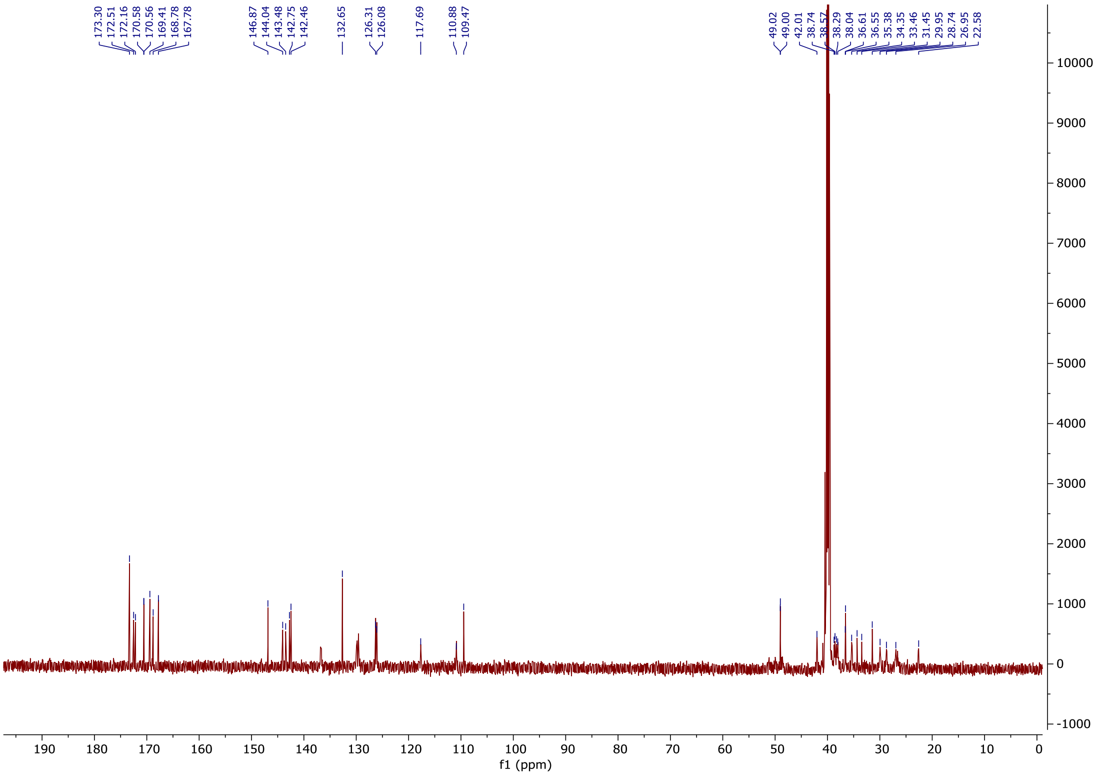

C9: N-(2-((4-((2-(2,6-dioxopiperidin-3-yl)-1,3-dioxoisoindolin-4-yl)amino)butyl)amino)-2-oxo-1-(4-(trifluoromethyl)phenyl)ethyl)-N-propylbutyramide:

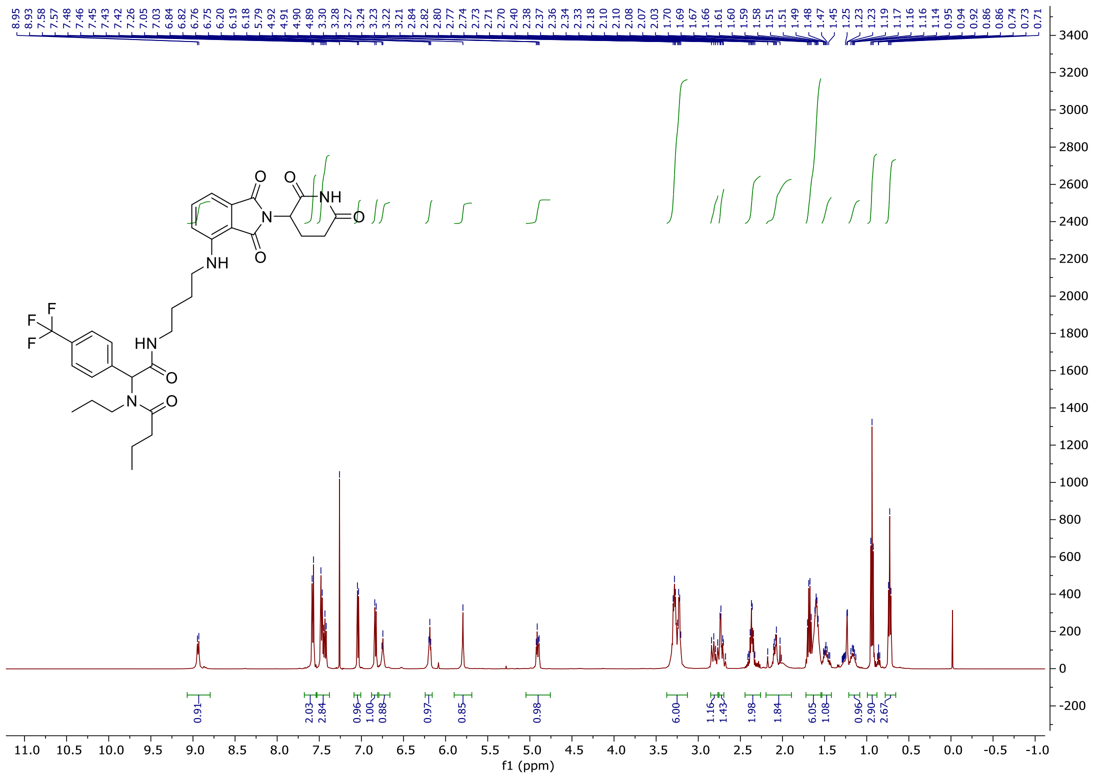

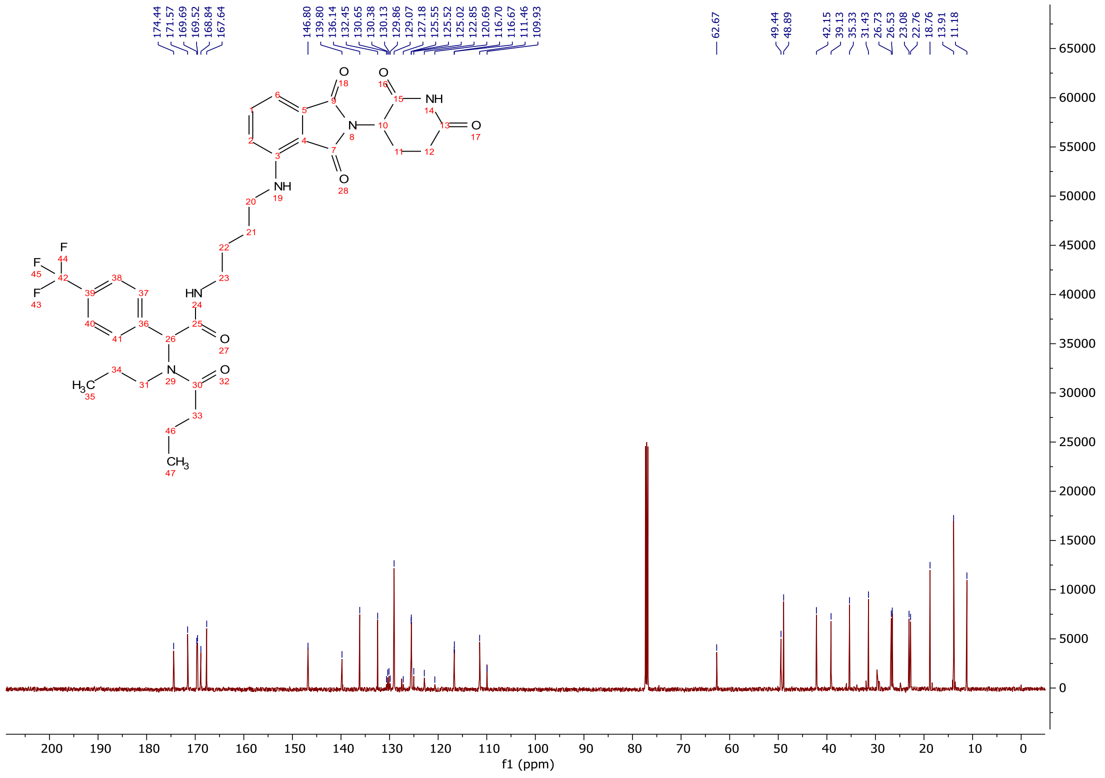

F9: N-(2-((4-((2-(2,6-dioxopiperidin-3-yl)-1,3-dioxoisoindolin-4-yl)amino)butyl)amino)-2-oxoethyl)-N-(4-methoxyphenethyl)-1-methylcyclohexane-1-carboxamide:

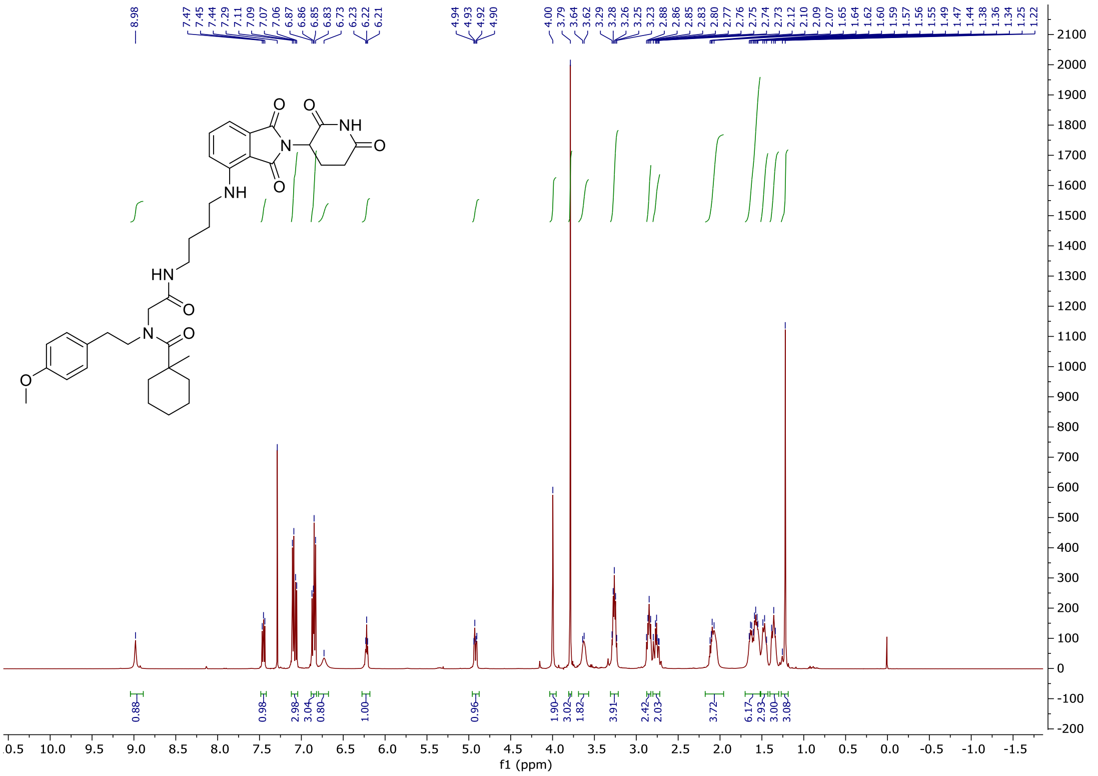

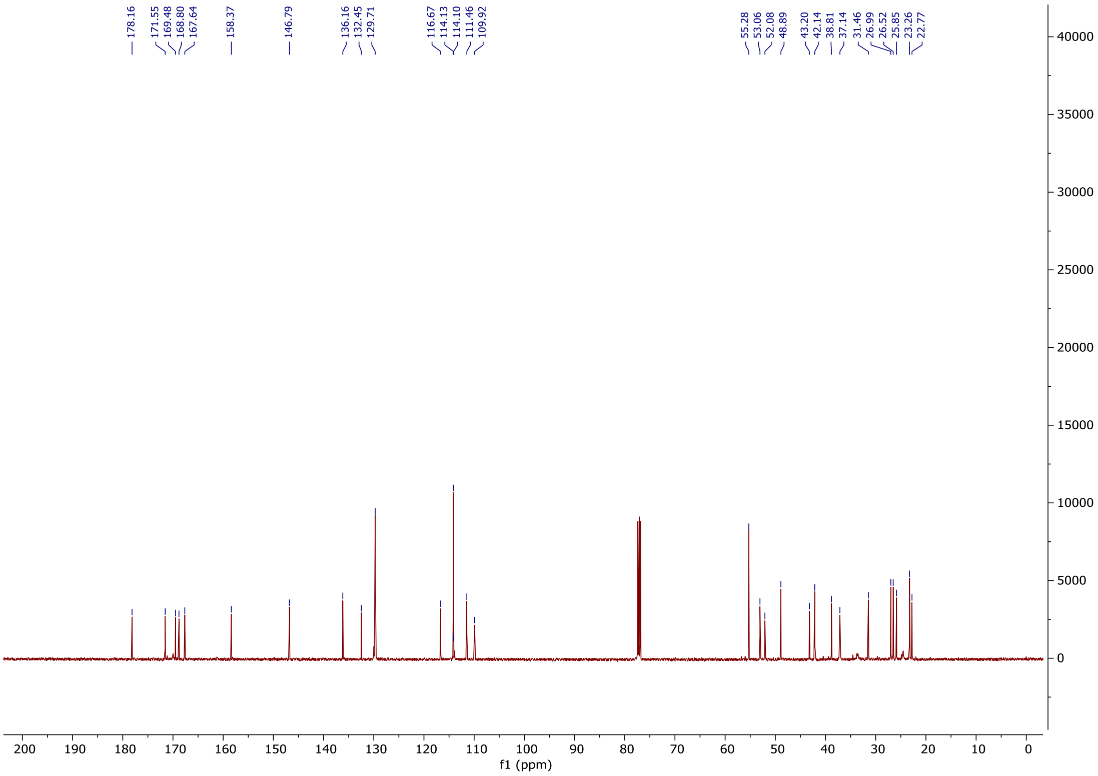

N9: 4-((4-(5-(((3,4-dimethylphenyl)amino)(pyridin-3-yl)methyl)-1H-tetrazol-1-yl)butyl)amino)-2-(2,6-dioxopiperidin-3-yl)isoindoline-1,3-dione:

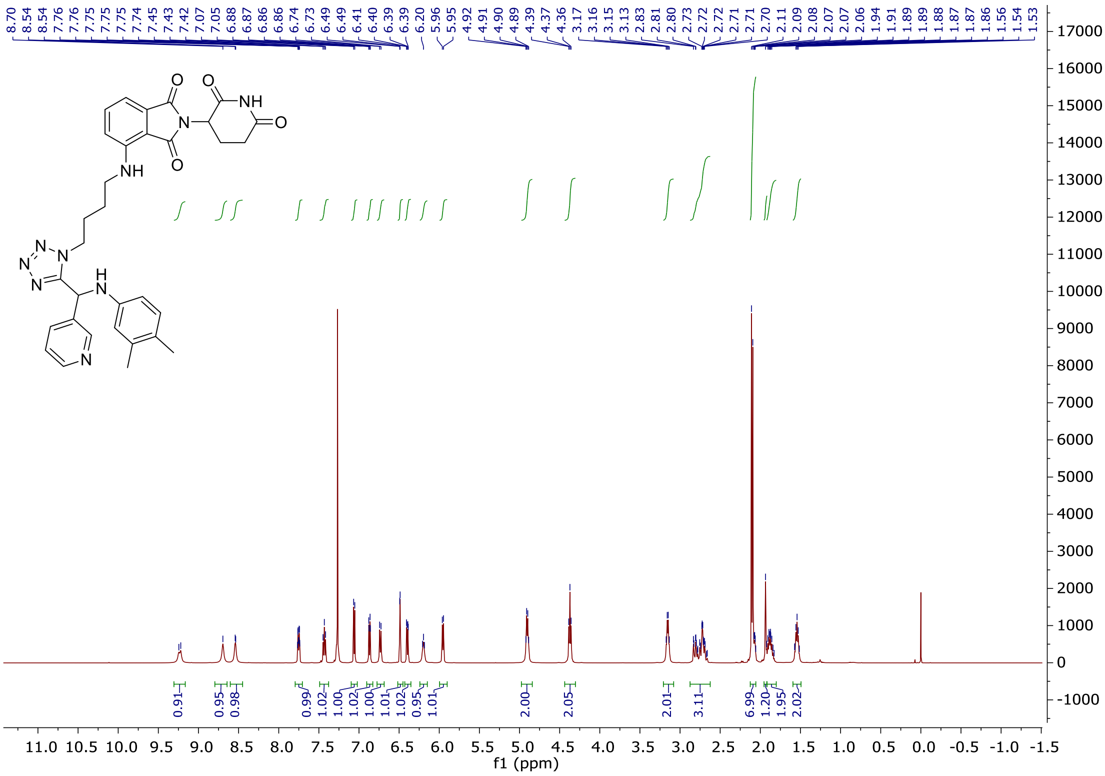

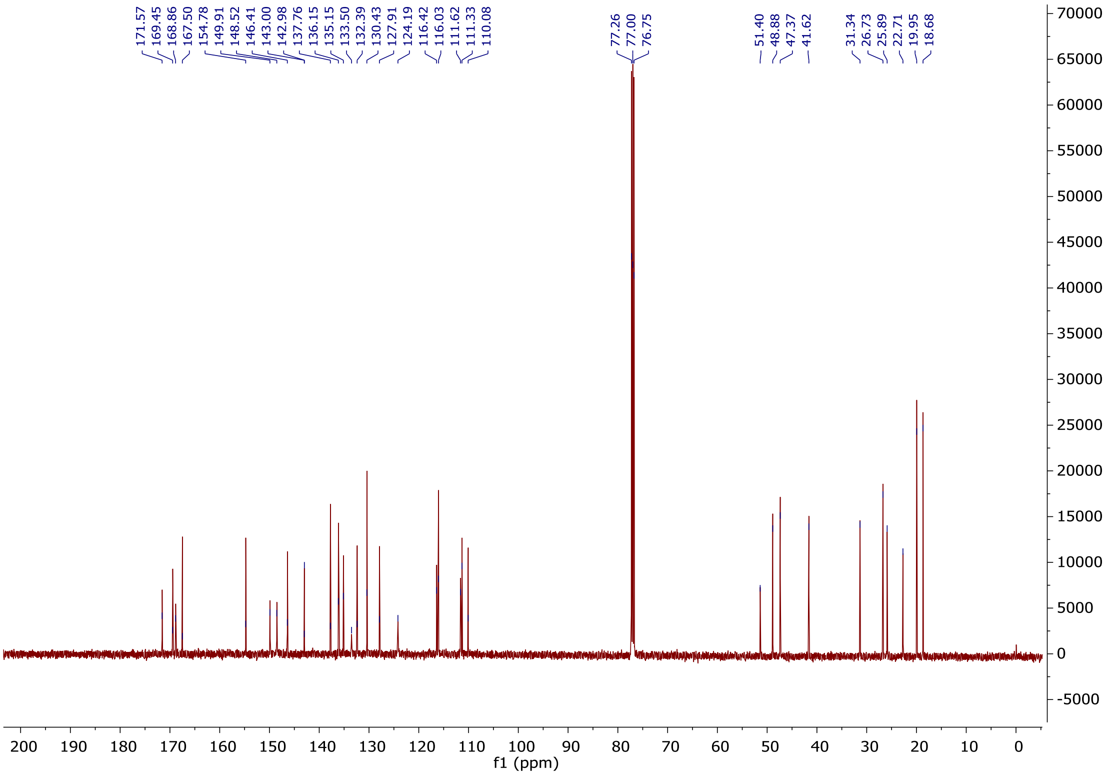

O9: 4-((4-(5-(4-((3,4-dimethylphenyl)amino)-1-methylpiperidin-4-yl)-1H-tetrazol-1-yl)butyl)amino)-2-(2,6-dioxopiperidin-3-yl)isoindoline-1,3-dione:

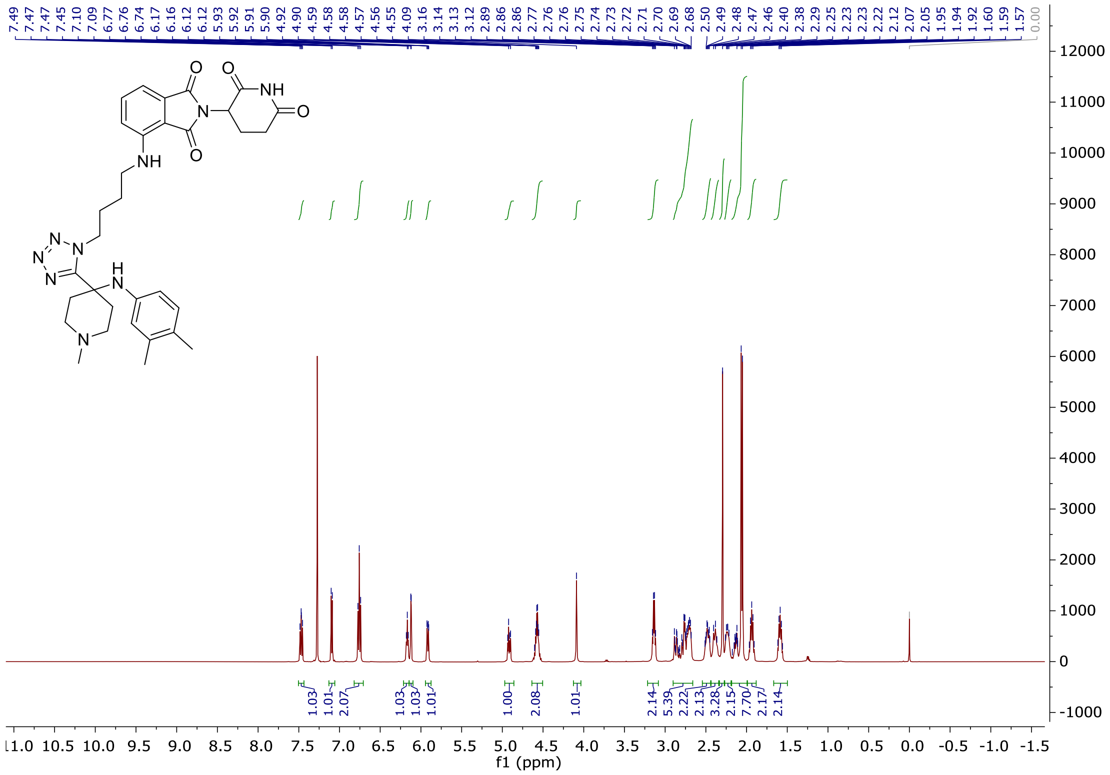

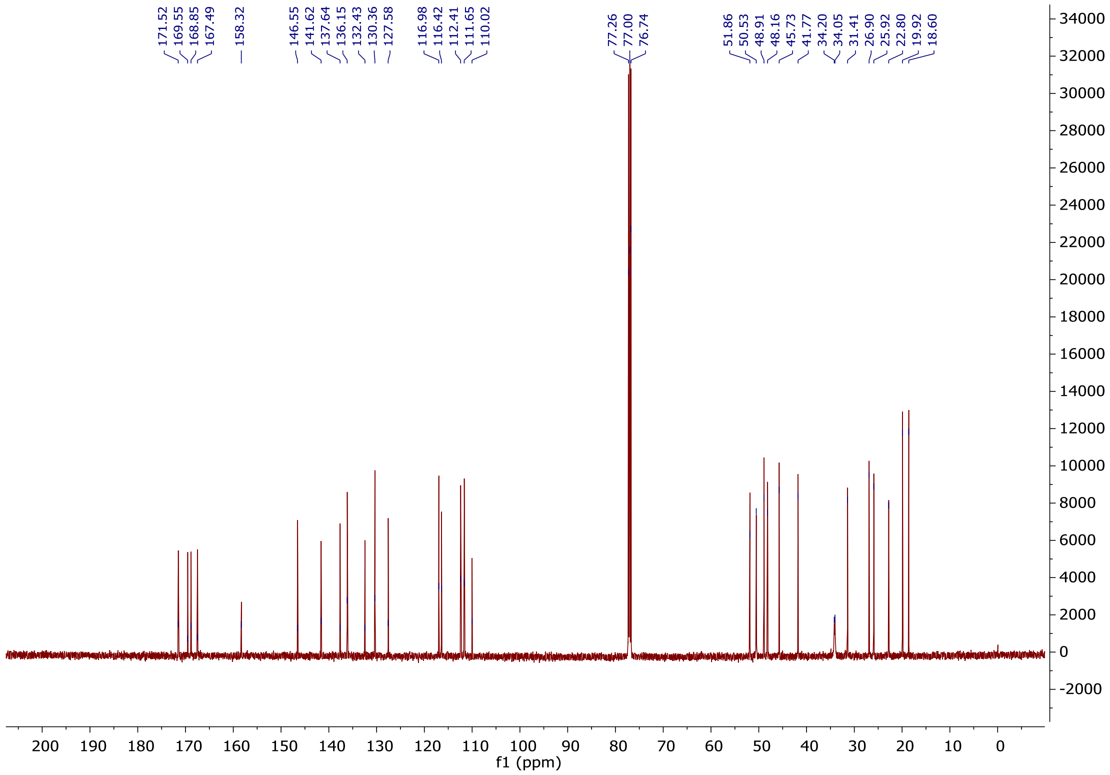

F11: 2-(bicyclo[2.2.1]heptan-2-yl)-N-(2-((4-((2-(2,6-dioxopiperidin-3-yl)-1,3-dioxoisoindolin-4-yl)amino)butyl)amino)-2-oxoethyl)-N-propylacetamide:

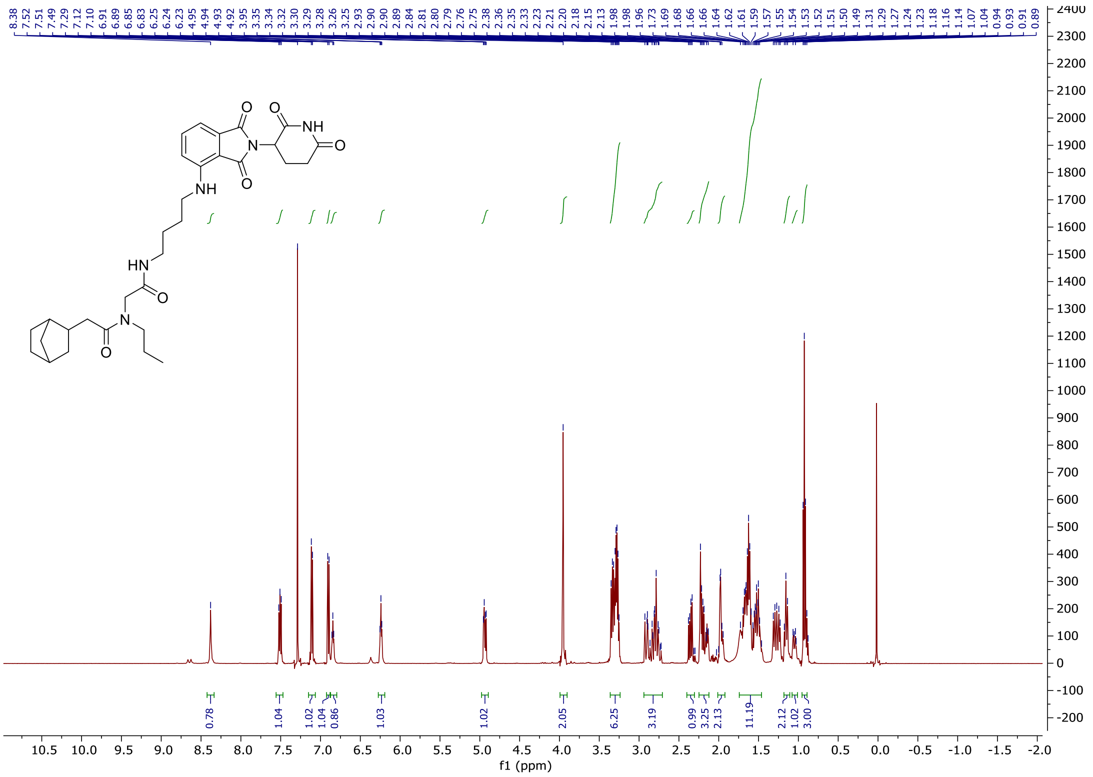

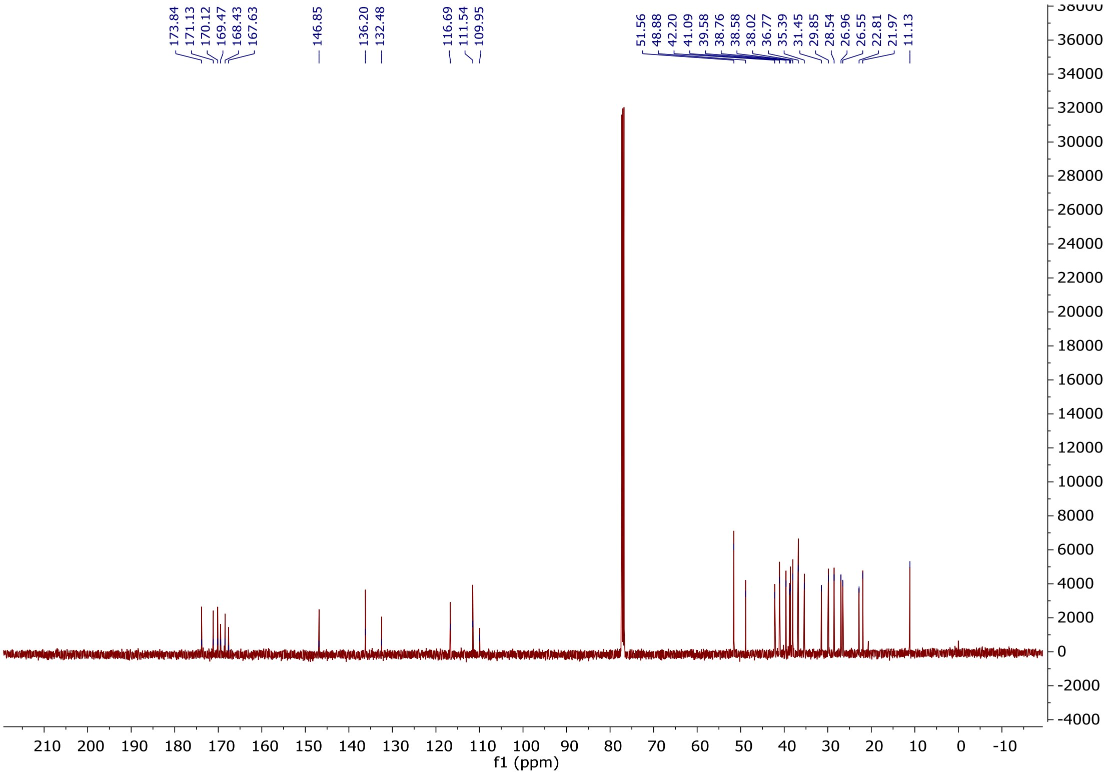

K11: 2-cyclohexyl-N-(2-((4-((2-(2,6-dioxopiperidin-3-yl)-1,3-dioxoisoindolin-4-yl)amino)butyl)amino)-2-oxoethyl)-N-(4-(trifluoromethyl)benzyl)acetamide:

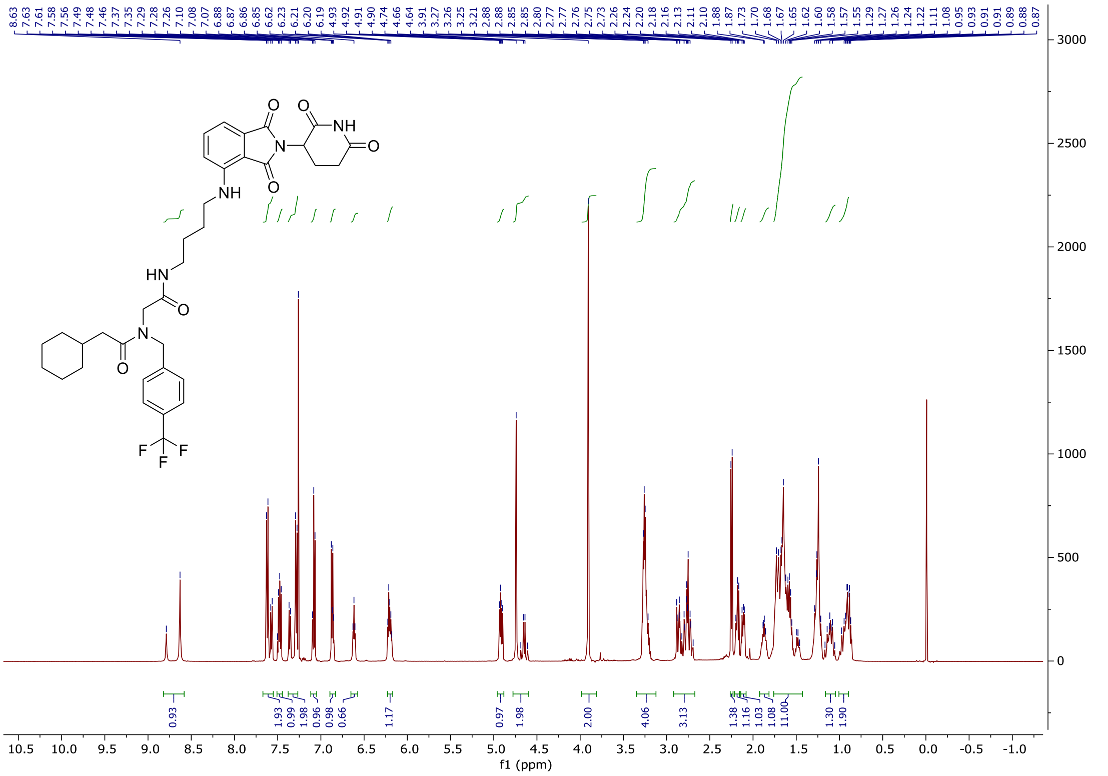

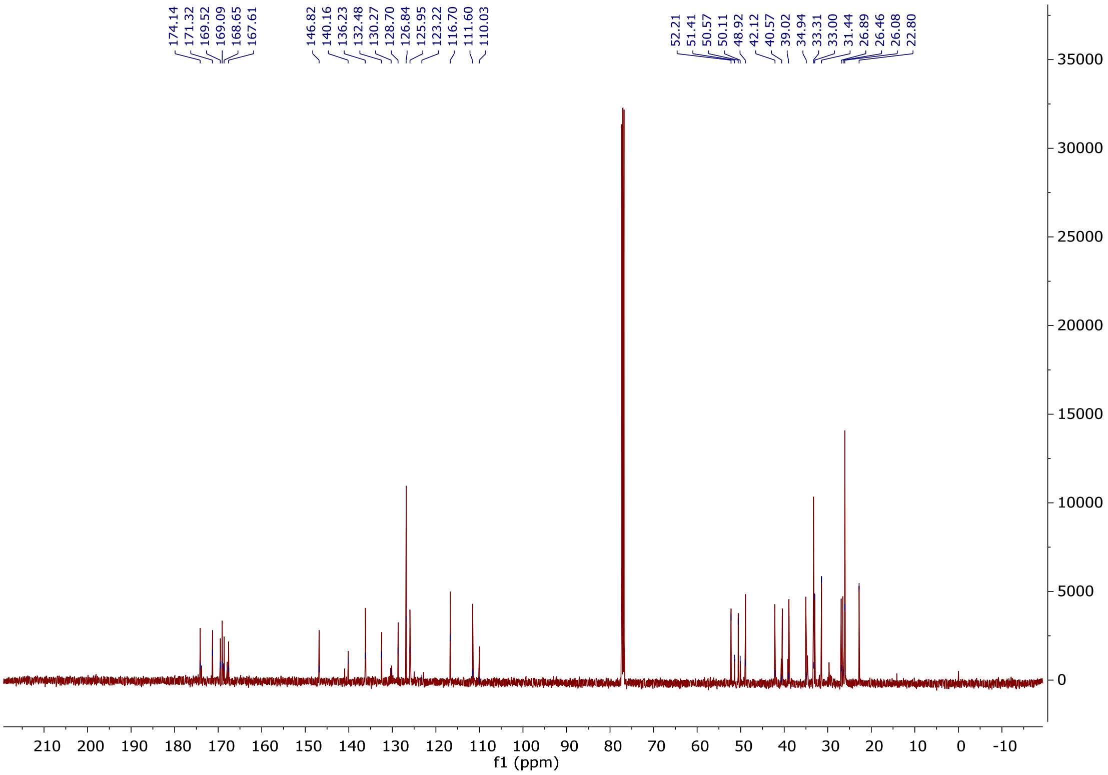

M11: 4-((4-(5-(((cyclopropylmethyl)amino)(phenyl)methyl)-1H-tetrazol-1-yl)butyl)amino)-2-(2,6-dioxopiperidin-3-yl)isoindoline-1,3-dione:

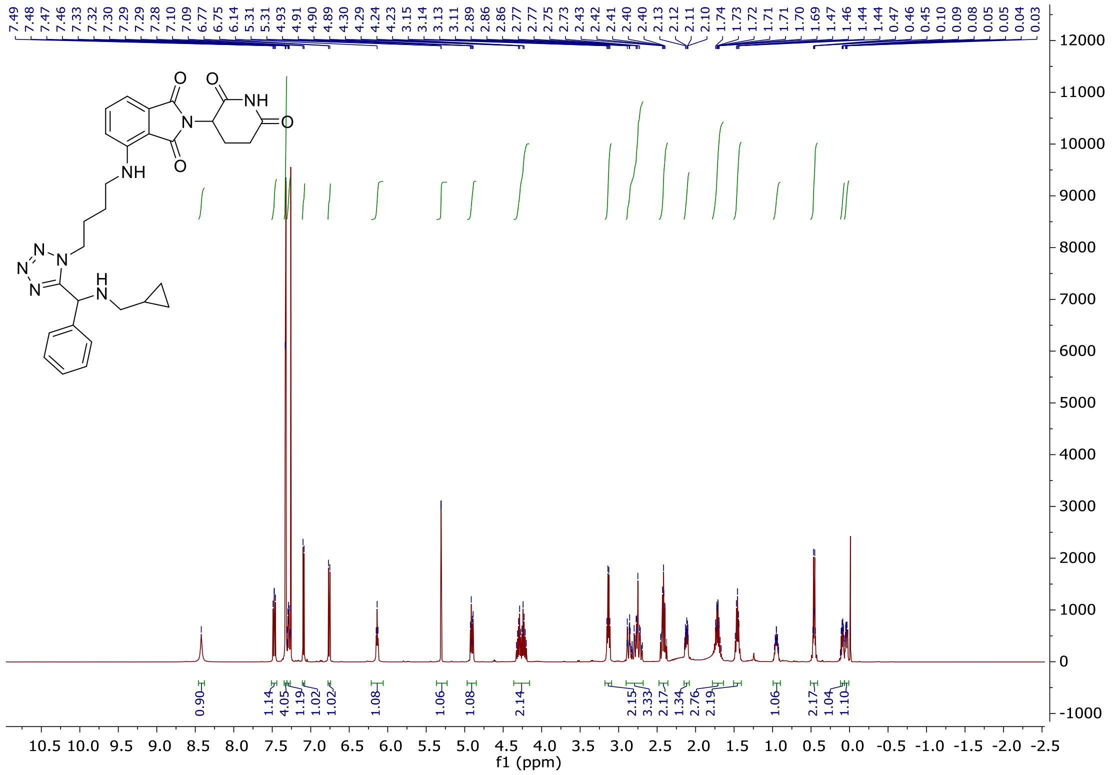

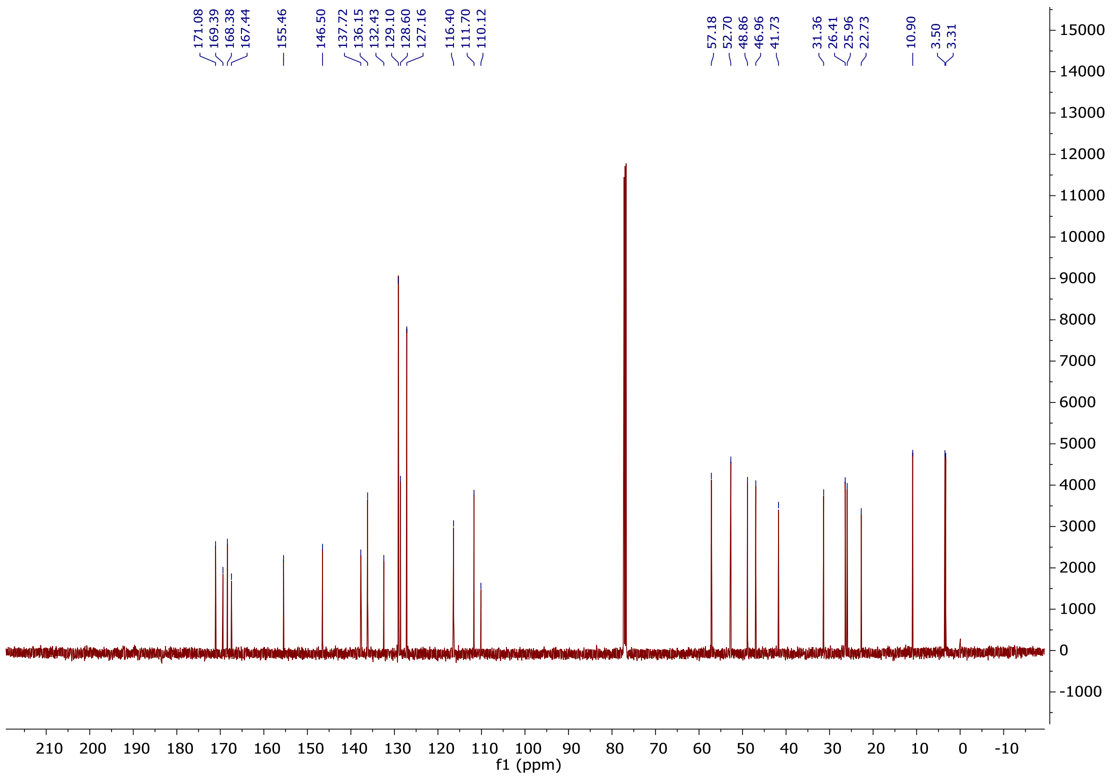

H12: N-(2-((4-((2-(2,6-dioxopiperidin-3-yl)-1,3-dioxoisoindolin-4-yl)amino)butyl)amino)-2-oxoethyl)-N-propylpropionamide:

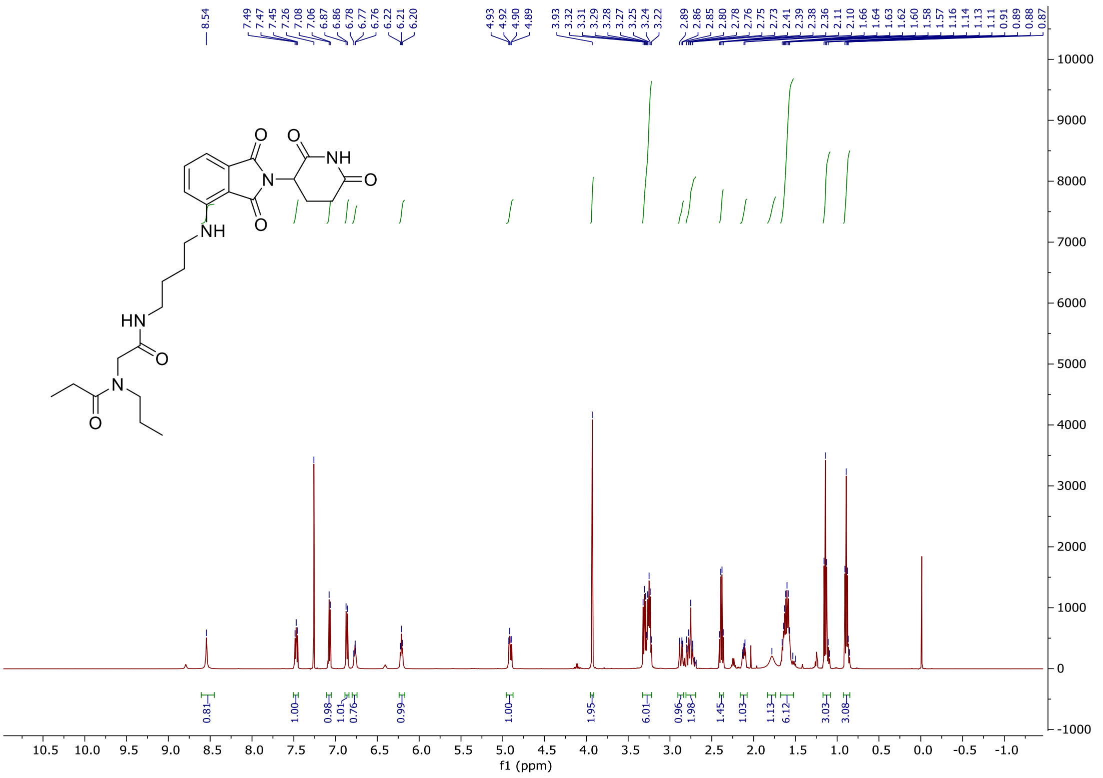

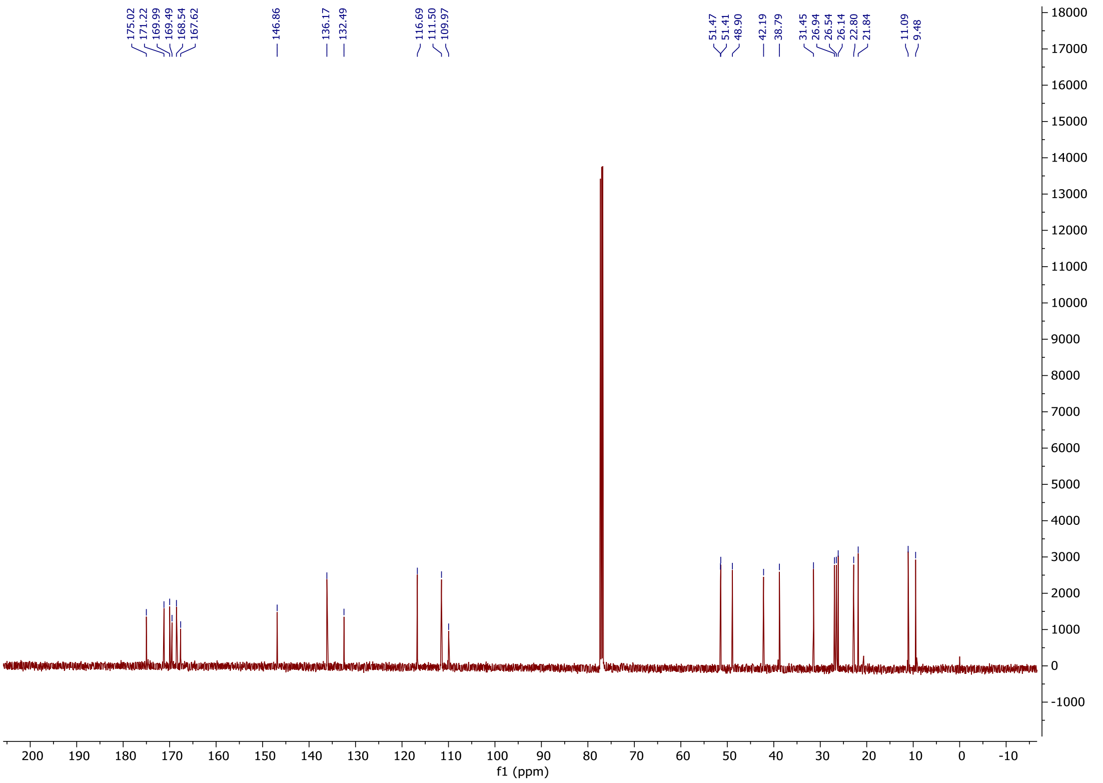

G13: 2-(cyclopent-2-en-1-yl)-N-(2-((4-((2-(2,6-dioxopiperidin-3-yl)-1,3-dioxoisoindolin-4-yl)amino)butyl)amino)-2-oxoethyl)-N-(2-hydroxyethyl)acetamide:

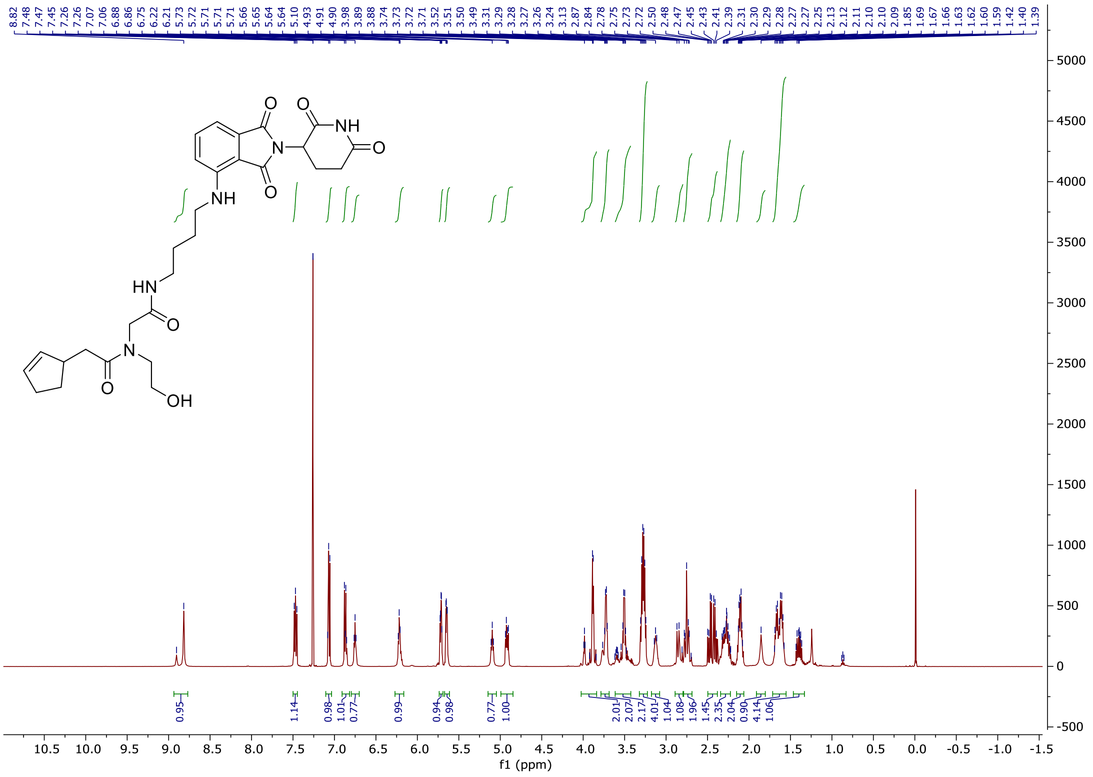

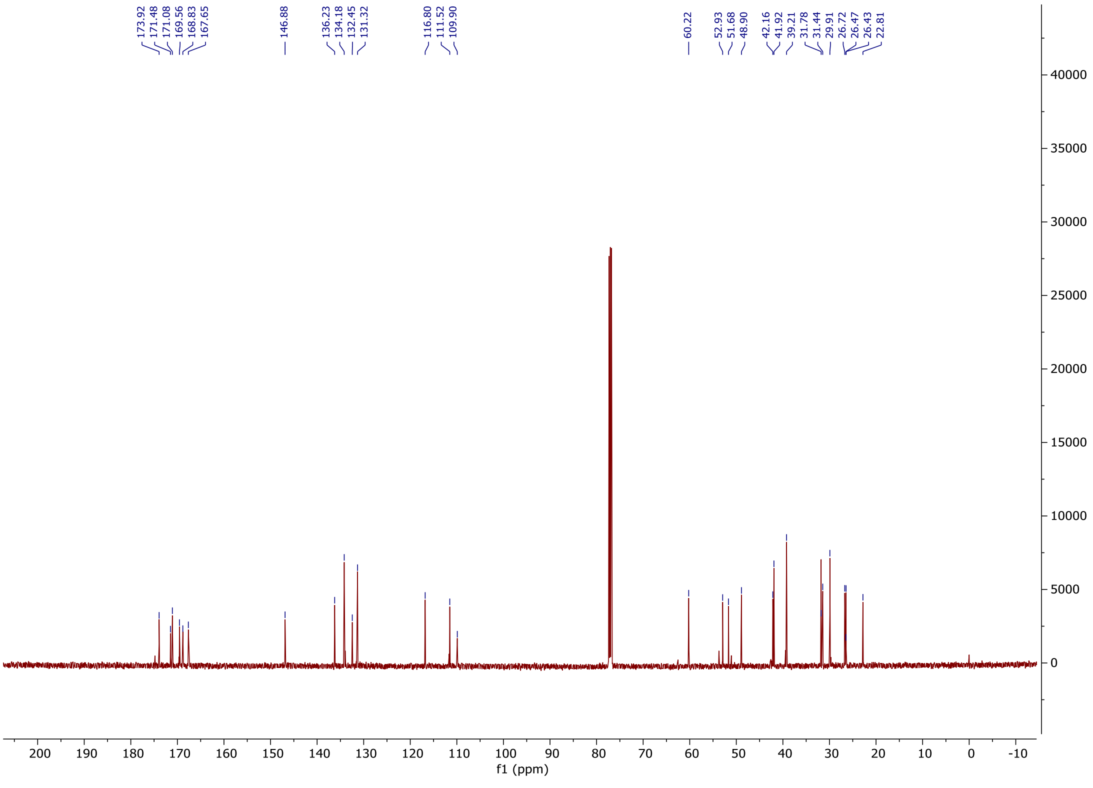

L13: 2-(2,6-dioxopiperidin-3-yl)-4-((4-(5-(1-((thiophen-3-ylmethyl)amino)cyclohexyl)-1H-tetrazol-1-yl)butyl)amino)isoindoline-1,3-dione:

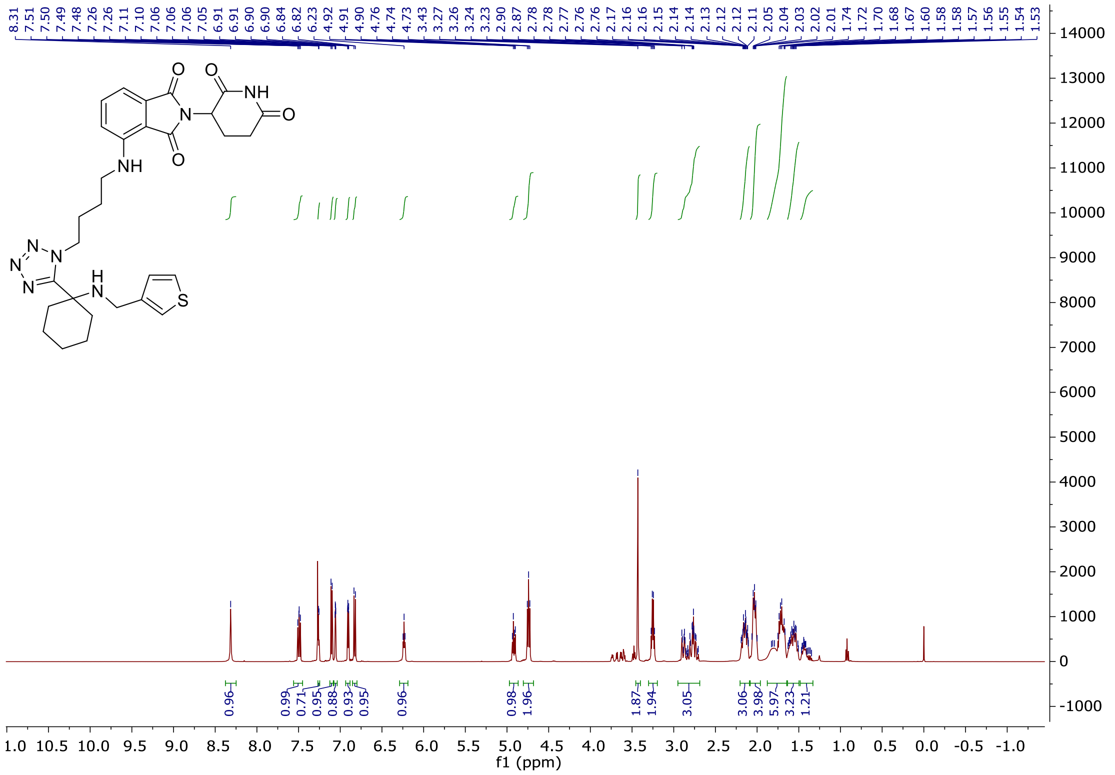

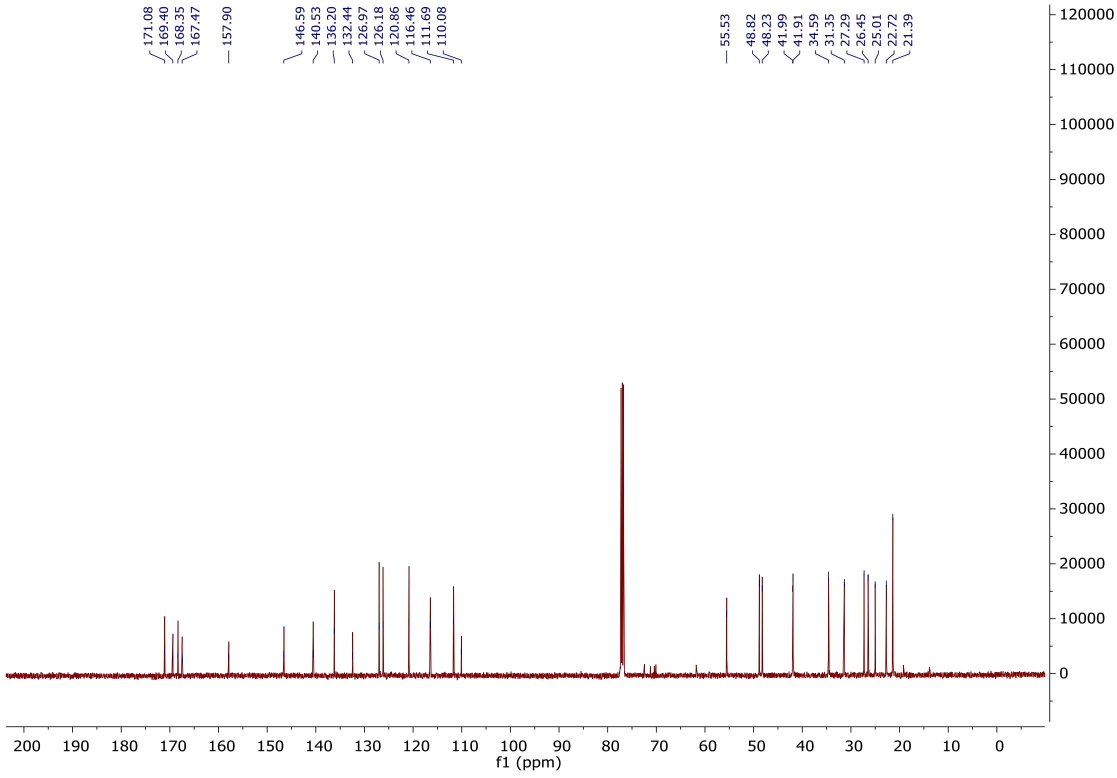

E14: N-(4-((2-(2,6-dioxopiperidin-3-yl)-1,3-dioxoisoindolin-4-yl)amino)butyl)-2-(N-isobutylformamido)-2-(3-methoxyphenyl)acetamide:

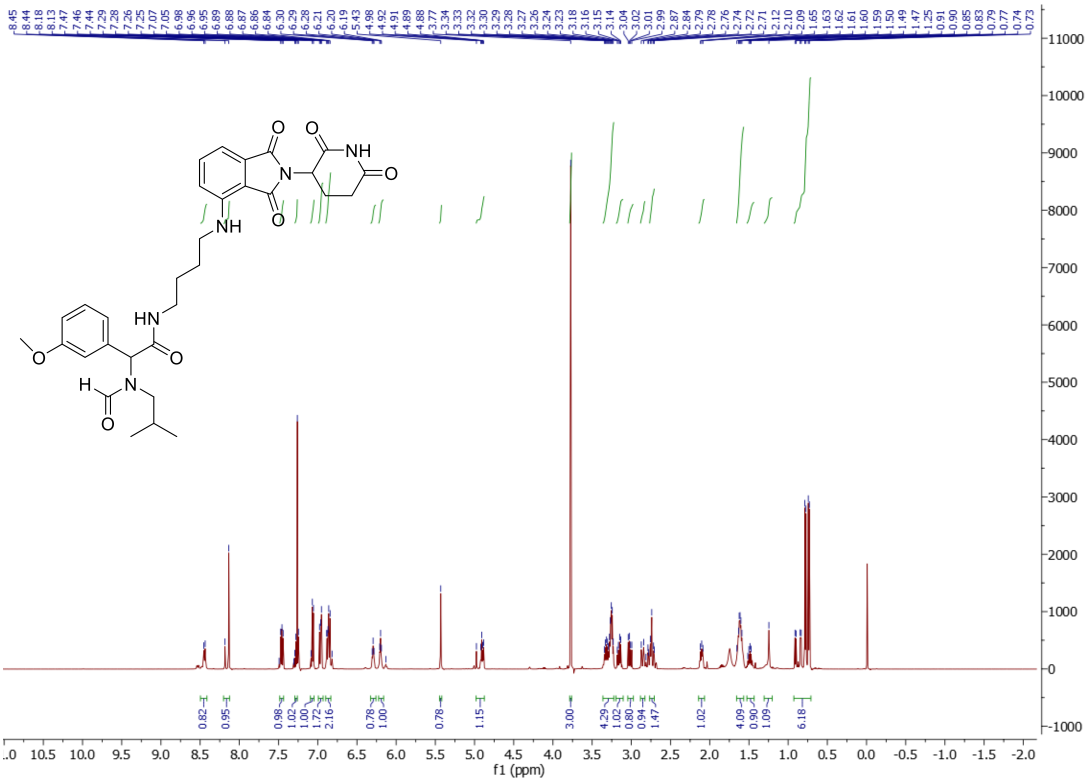

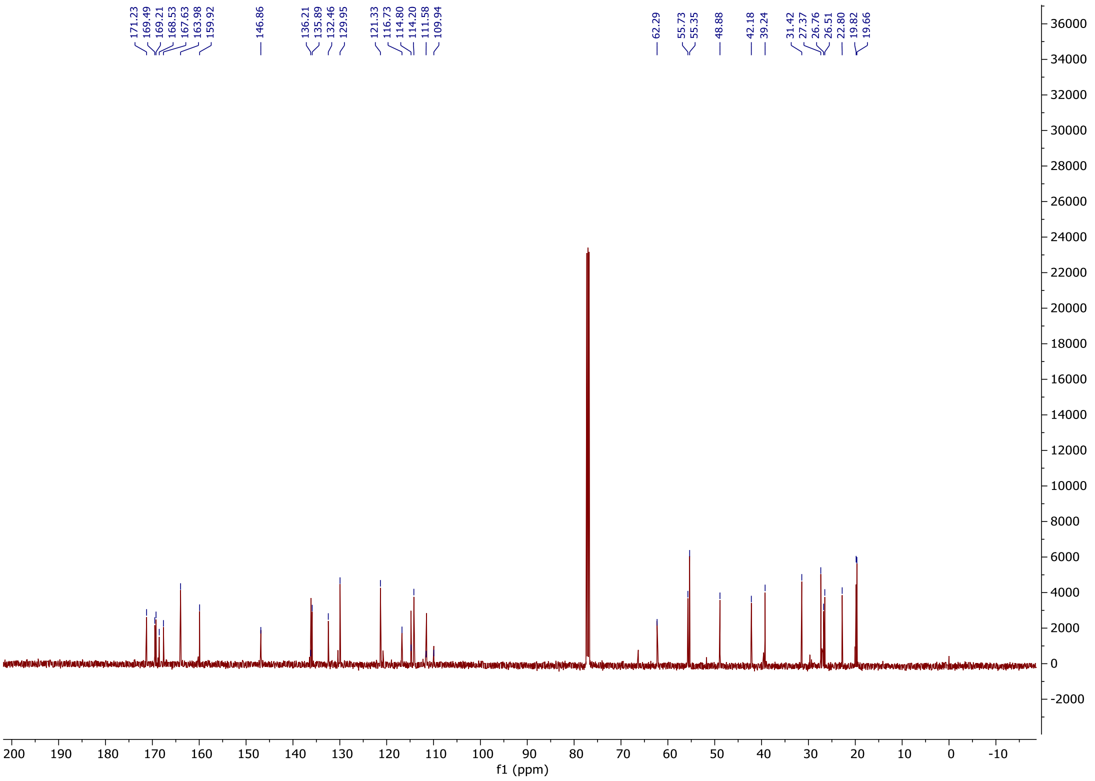

L18: 4-((4-(5-(1-((2,2-dimethoxyethyl)amino)cyclohexyl)-1H-tetrazol-1-yl)butyl)amino)-2-(2,6-dioxopiperidin-3-yl)isoindoline-1,3-dione:

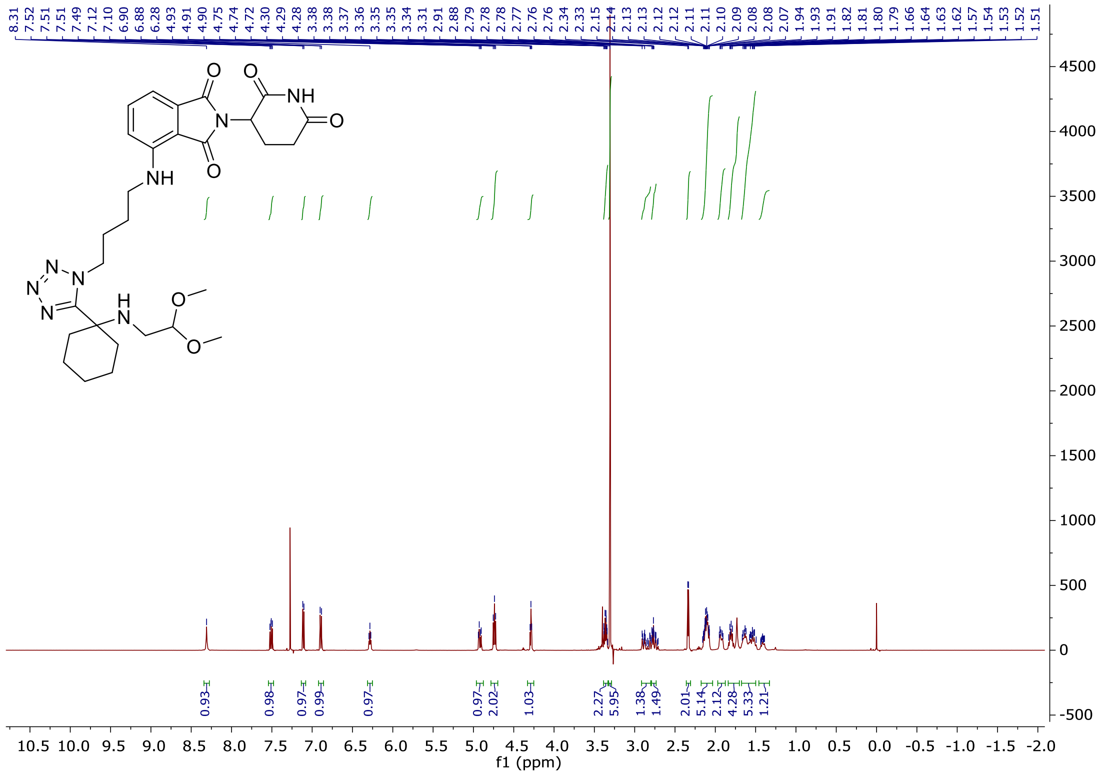

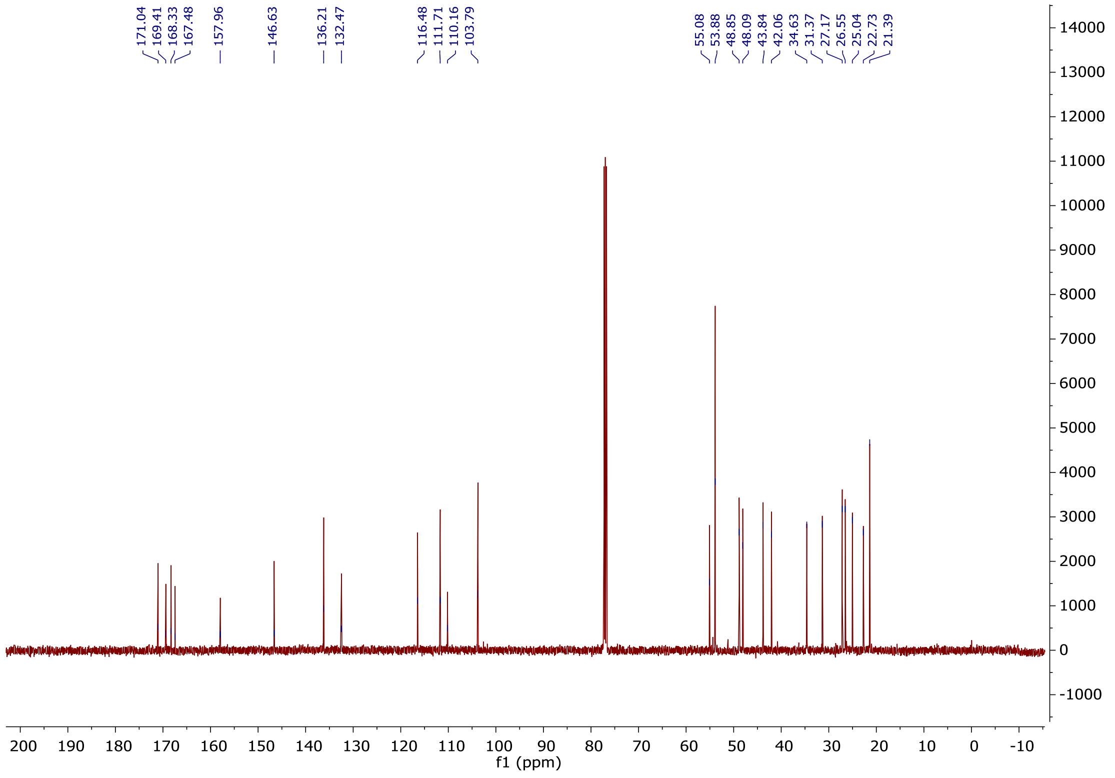

I19: N-(2-((4-((2-(2,6-dioxopiperidin-3-yl)-1,3-dioxoisoindolin-4-yl)amino)butyl)amino)-2-oxoethyl)-N-(2-hydroxyethyl)-2-methoxybenzamide:

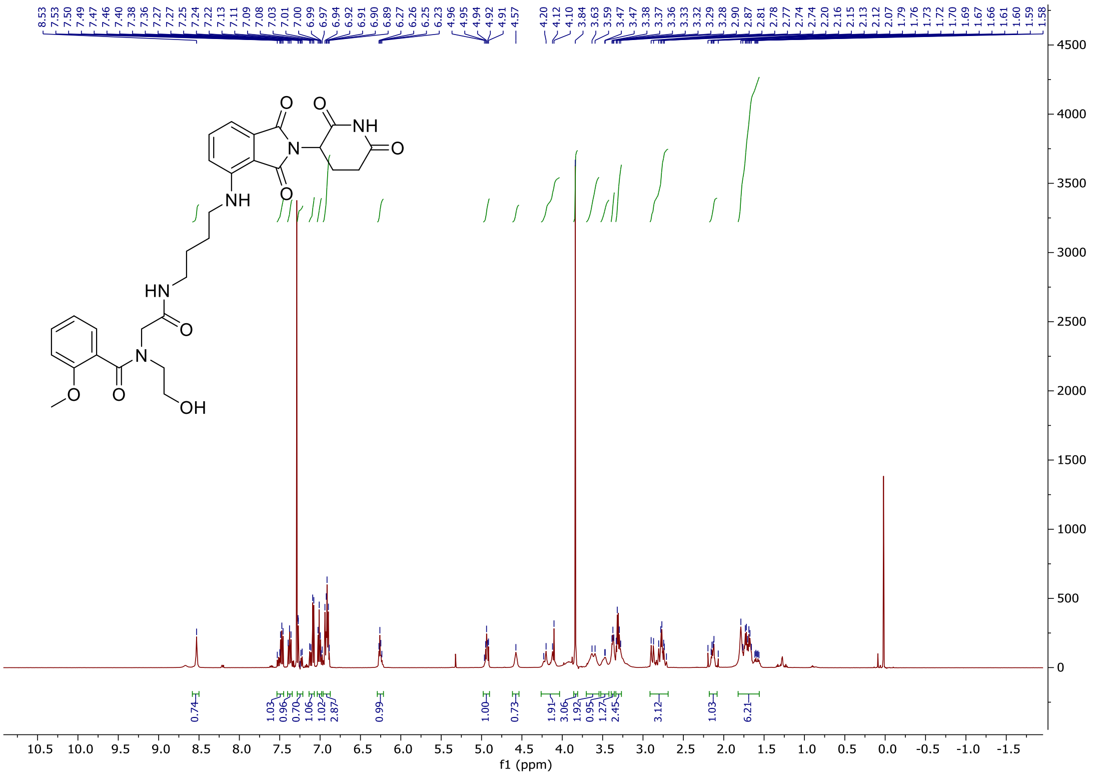

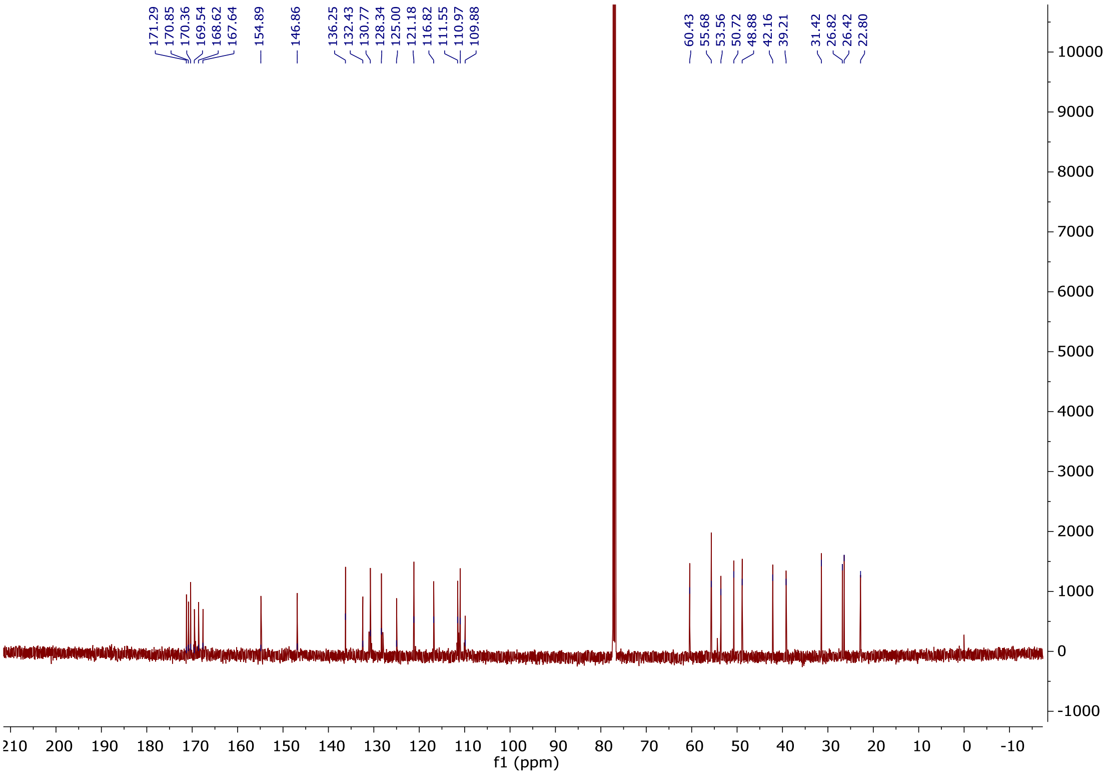

H23: 2-(cyclopent-2-en-1-yl)-N-(2-((4-((2-(2,6-dioxopiperidin-3-yl)-1,3-dioxoisoindolin-4-yl)amino)butyl)amino)-2-oxoethyl)-N-(4-sulfamoylphenethyl)acetamide:

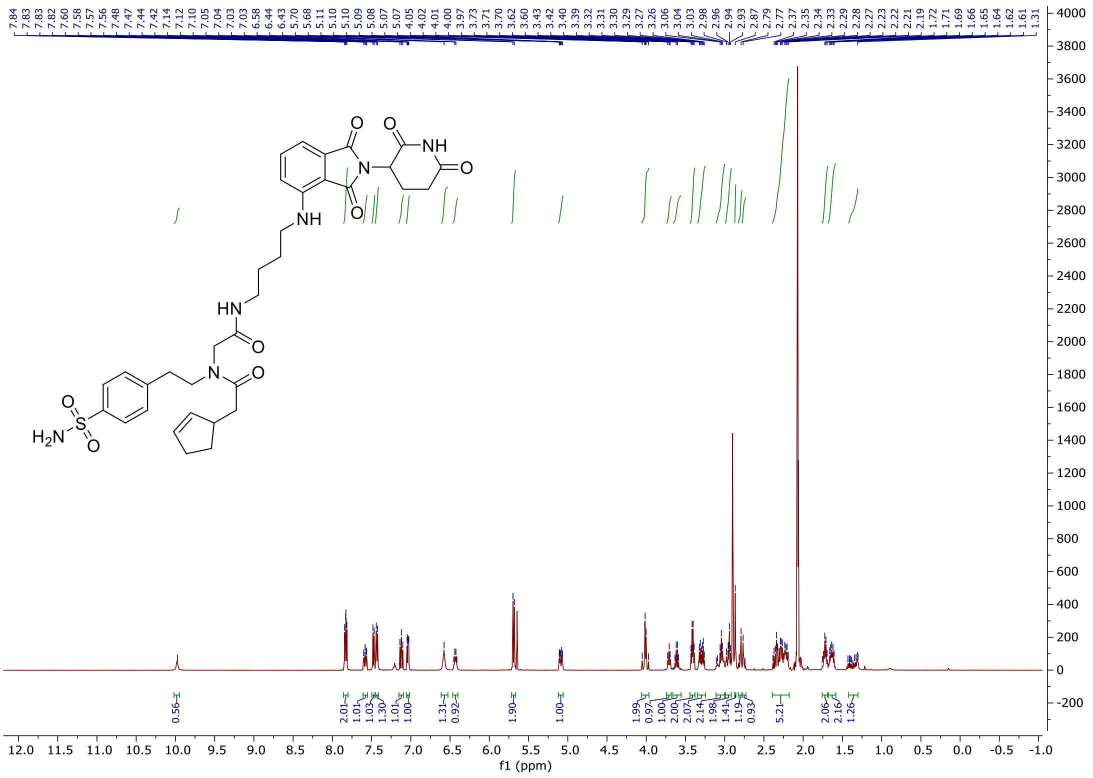

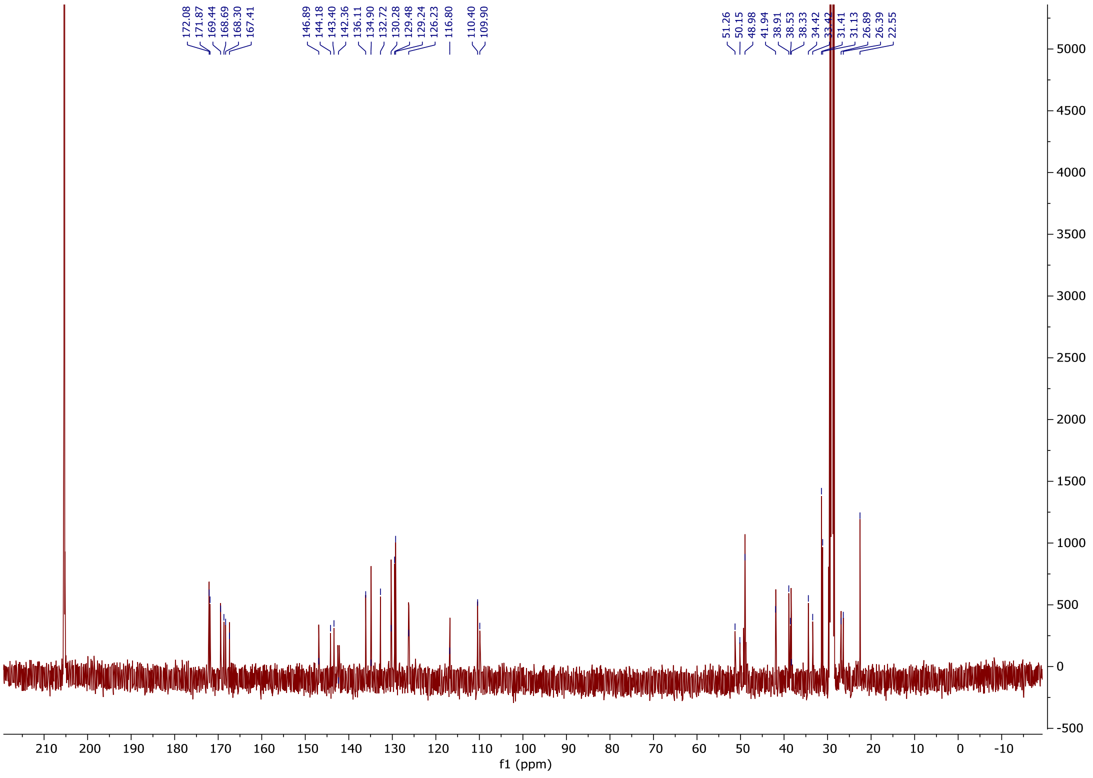

P24: 2-(2,6-dioxopiperidin-3-yl)-4-((4-(5-(1-((furan-2-ylmethyl)amino)cyclopentyl)-1H-tetrazol-1-yl)butyl)amino)isoindoline-1,3-dione:

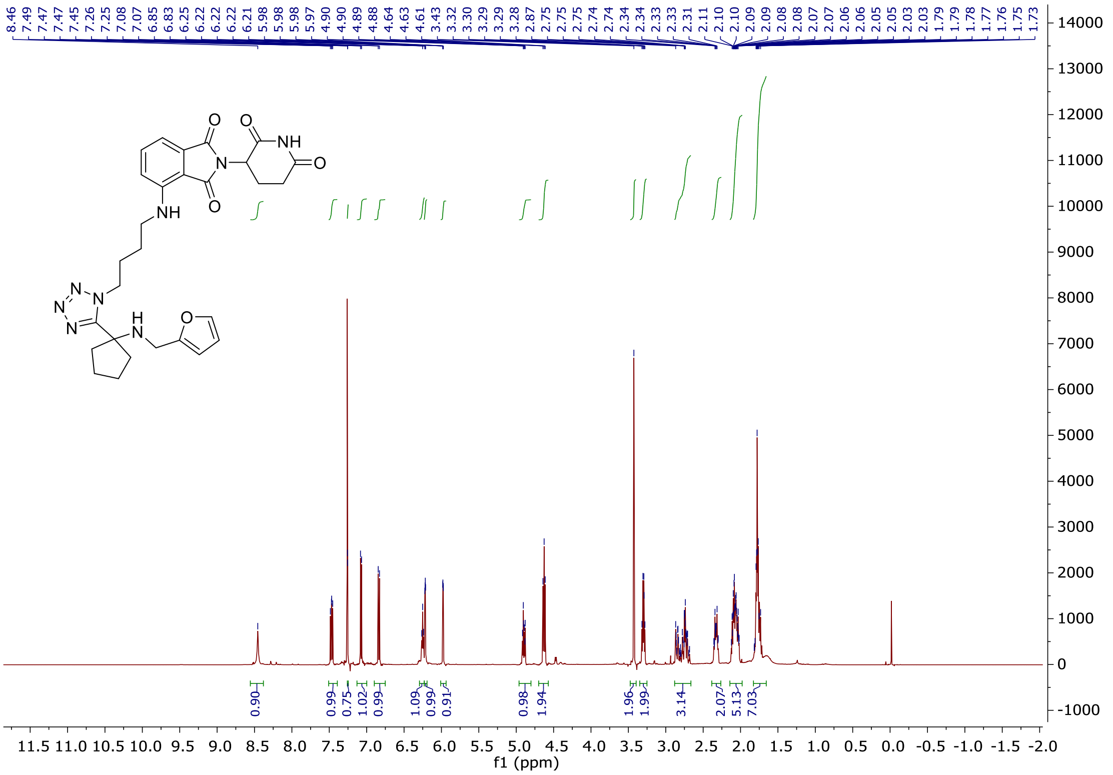

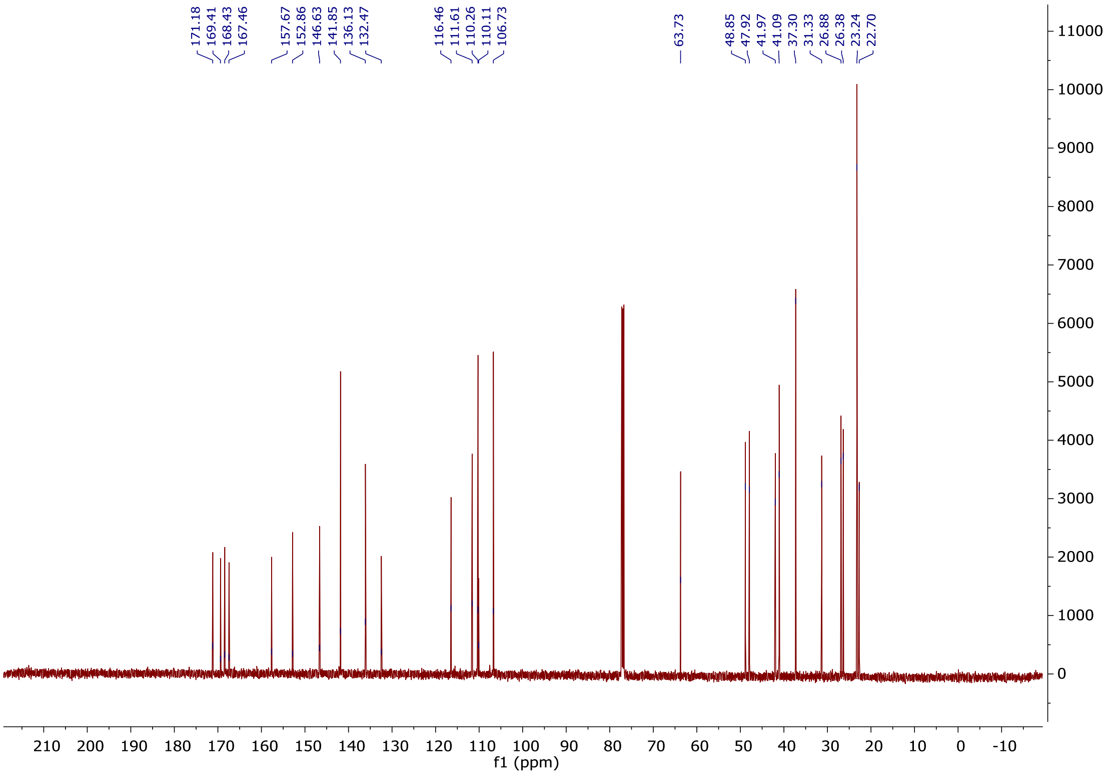

References

1) Y. Huang, S. Wolf, M. Bista, L. Meireles, C. Camacho, T. A. Holak and A. Dömling, *Chem. Biol. Drug. Des.*, 2010, **76**, 116-129.
2) Osipyan, A.; Shaabani, S.; Warmerdam, R.; Shishkina, S. V.; Boltz, H.;, Automated, accelerated nanoscale synthesis of iminopyrrolidines. *Angew. Chem. Int. Ed*., 2020, **59**, 12423-12427.
